# What do people think about genetic engineering? A systematic review of questionnaire surveys before and after the introduction of CRISPR

**DOI:** 10.3389/fgeed.2023.1284547

**Published:** 2023-12-19

**Authors:** Pedro Dias Ramos, Maria Strecht Almeida, Ingrid Anna Sofia Olsson

**Affiliations:** ^1^ i3S–Instituto de Investigação e Inovação em Saúde, Universidade do Porto, Porto, Portugal; ^2^ ICBAS–Instituto de Ciências Biomédicas Abel Salazar, Universidade do Porto, Porto, Portugal

**Keywords:** systematic review, public attitude, genetic engineering, genome editing, CRISPR, questionnaires, surveys

## Abstract

The advent of CRISPR-Cas9 in 2012 started revolutionizing the field of genetics by broadening the access to a method for precise modification of the human genome. It also brought renewed attention to the ethical issues of genetic modification and the societal acceptance of technology for this purpose. So far, many surveys assessing public attitudes toward genetic modification have been conducted worldwide. Here, we present the results of a systematic review of primary publications of surveys addressing public attitudes toward genetic modification as well as the awareness and knowledge about the technology required for genetic modification. A total of 53 primary publications (1987–2020) focusing on applications in humans and non-human animals were identified, covering countries in four continents. Of the 53 studies, 30 studies from until and including 2012 (pre-CRISPR) address gene therapy in humans and genetic modification of animals for food production and biomedical research. The remaining 23 studies from after 2013 (CRISPR) address gene editing in humans and animals. Across countries, respondents see gene therapy for disease treatment or prevention in humans as desirable and highly acceptable, whereas enhancement is generally met with opposition. When the study distinguishes between somatic and germline applications, somatic gene editing is generally accepted, whereas germline applications are met with ambivalence. The purpose of the application is also important for assessing attitudes toward genetically modified animals: modification in food production is much less accepted than for biomedical application in pre-CRISPR studies. A relationship between knowledge/awareness and attitude toward genetic modification is often present. A critical appraisal of methodology quality in the primary publications with regards to sampling and questionnaire design, development, and administration shows that there is considerable scope for improvement in the reporting of methodological detail. Lack of information is more common in earlier studies, which probably reflects the changing practice in the field.

## Introduction

The advent of CRISPR-Cas9 in 2012 started revolutionizing the field of genetics by democratizing the access to a method for precise modification of the mammalian genome (Camporesi and Cavaliere, 2016; Barrangou and Horvath, 2017). The finding that the technique is straightforward and of low cost—while being precise and efficient—underlies the wide uptake of CRISPR-Cas9 by research groups and industries (Camporesi and Cavaliere, 2016; Nordberg et al., 2018). This has resulted in an explosion of laboratories engaging in research using genetic modification of organisms, including applications in clinical practice, biomedical research, food production, and for environmental purposes (Nordberg et al., 2018; Brokowski and Adli, 2019). The possibility of CRISPR-Cas9 application to human embryos has nonetheless raised concern among scientists and in society and led to revisit previous regulations on human genetic manipulation, such as Article 13 of the Oviedo Convention, the Universal Declaration on the Human Genome and Human Rights, and the EU Charter of Fundamental Rights (Nordberg et al., 2018). The first years of CRISPR-Cas9 were marked by uncertainty, and an international moratorium on human germline manipulation was adopted by a range of countries (Isasi et al., 2016; Boggio et al., 2019; Brokowski and Adli, 2019). However, in 2018, media announced the first case of human embryo manipulation that resulted in the birth of the first gene-edited twin babies and the expected arrival of another gene-edited baby in the summer of 2019 (Hirsch et al., 2019; Meagher et al., 2020). This story initiated a frenzy of media articles, generally characterized by strong and general disapproval, conveying concern that scientists were “crossing the line” and almost unanimous rejection by members of the scientific community (Nordberg et al., 2018; Morrison and de Saille, 2019). The discussion around CRISPR-Cas9 has also reignited concerns about gene editing of animals, including those used for food, and their potential release into the environment and the food supply chain (Caplan et al., 2015).

By the time the CRISPR-Cas9 technique became available, the question of genetic modification of living organisms had already been discussed for more than 3 decades. Following the first study by Thomas and Capecchi in 1987, where recombinant DNA could be transferred as a tool to mammalian cells, the first international conference in 1975 led to the creation of the Recombinant Advisory Committee (RAC) to discuss ethical and societal issues related to the application of this new biotechnology tool (Hurlbut et al., 2015; Rufo and Ficorilli, 2019). Subsequent landmark events where genetic engineering was applied to humans, such as the first clinical introduction of retrovirus in gene-modified cells by Rosenberg in 1989 (Hanna et al., 2017), the death of Jesse Gelsinger in 1999 after gene therapy intervention to treat a metabolic disorder (Caplan, 2019), and the death of X-SCID patients in a gene therapy trial in 2002 (Couzin and Kaiser, 2005), were reflected in public distrust and a delay in the development of gene therapy over the first decade of the 21st century. Other major scientific milestones include the first genome-edited embryos (Liang et al., 2015), human clinical trials with genome editing therapies (ClinicalTrials.gov, 2016a; ClinicalTrials.gov, 2016b; ClinicalTrials.gov, 2018), the genome-edited human babies referred to above, and the attribution of the 2020 Nobel Prize in Chemistry to Jennifer Doudna and Emmanuelle Charpentier for their work leading to the CRISPR technology (Royal Swedish Academy of Science, 2020).

As it makes gene editing much easier and more widely applicable, CRISPR-Cas9 comes across as a technology perceived as both promising and threatening and, as such, is particularly interesting in the context of initiatives such as RRI (Responsible Research and Innovation), which aim to open up research to society (Shelley-Egan et al., 2020). The underlying objective is to align the research and development of new technologies with societal values and priorities. Understanding public knowledge and awareness of a new technology is an important part of the process, as is the measurement of citizens’ attitudes toward such development, for two main reasons. First, in representative democracies, questionnaires are important sources of information about how citizens position themselves in specific issues. Second, it is important to understand how receptive citizens are to adopting new technologies in their daily lives.

Opinion surveys measure the views of society within a given context in relation to a certain topic, often with a cross-sectional approach that measures opinions at a specific time-point and allows for comparison, such as between countries or regions but not over time (Stockemer, 2019a; Stockemer, 2019b). When used as research instruments, surveys of public opinion are designed to provide quantitative information that allows researchers to answer underlying research questions by assessing the attitudes of surveyed people (Haddock and Maio, 2008). A critical appraisal of the study methodology is an important complement to a systematic review of study outcomes. Despite being most common in reviews of randomized clinical trials, critical appraisal is relevant for many types of studies, including quantitative, qualitative, mixed-methods, and surveys (National Health and Medical Research Council, 2000a; National Health and Medical Research Council, 2000b; Moher et al., 2009; Crowe and Sheppard, 2011; Nolan et al., 2012; Pace et al., 2012; Oluka et al., 2014). An important aspect of methodological quality is the survey instrument, that is, the set of questions and the accompanying measurement scales such as Likert and semantic differential scales, which are constructs that need to be evaluated in terms of validity and reliability before the survey is administered (Haddock and Maio, 2008; Boateng et al., 2018; Hair et al., 2019). In systematic reviews of quantitative questionnaire studies, critical appraisal also includes the validity and how representative the sample is of the population under study, how the variables have been defined, whether potential biases are considered, and other factors that may interfere with result interpretation (COGEM, 2018).

The aim of the present systematic review is to map the existing body of evidence concerning public attitudes toward genetic modification since the first survey on the topic was applied nearly 35 years ago. The review includes 53 primary publications covering countries in Asia, Europe, North America, South America, and Oceania, integrating public attitudes and awareness and knowledge about genetic modification. Our approach is comprehensive as it includes cross-sectional surveys measuring public opinions on matters of biotechnology and genetic engineering when applied to humans and other animals and introduces critical appraisal as a means to assess the methodology quality surrounding questionnaire design, development, and administration together with population sampling and the main limitations and successes drawn from studies in this type of analysis. This systematic review will complement existing narrative reviews and perspective papers on the topic, such as Lassen et al. (2006); Condit (2010); Howell et al. (2020).

## Methodology

### Search

Web of Science (WOS) was selected as the primary source for scholarly publications, focusing the search to identify surveys done with citizens on three different themes: *gene therapy*, *genetically modified animals (GM animals)*, and *genome editing*. The search was conducted between July and November 2019 and reviewed again in February 2020 and August 2022. This database search was complemented with Google search engine to look specifically for the gray literature that could not be found through WOS, namely, governmental reports and other studies not published in academic journals. Although not peer-reviewed by academic scholars, their relevance for policy advising means this type of literature is worth considering (Haddaway et al., 2015; Piasecki et al., 2018). For the WOS search, the themes *gene therapy* and *GM animals* included only publications until 2012 since this was the year of the advent of CRISPR-Cas9 biotechnology, which changed the terminology of scientific articles from “genetic modification” to “genome editing.” Conversely and likewise, for the *genome editing* theme, only studies from 2013 onward were included. All WOS databases were investigated: WOS Core Collection, Current Contents Connect, Derwent Innovations Index, KCI—Korean Journal Database, MEDLINE^®^, Russian Science Citation Index, and SciELO Citation Index. Pilot studies were searched using different combinations of keywords until the identification of the final Boolean strings to be used for the searching process was completed (see Supplementary Material). For this, the numbers of publications retrieved from WOS for a specific combination of strings were analyzed, and only the ones with the highest numbers were considered. For *GM animals*, the different combinations of strings yielded the highest number of all, while for *gene therapy* and *genome editing* themes, it was irrelevant to add more string terms since it would always yield equal or lower numbers of publications. These allowed us to conduct the search in a broadened way, finding the most publications possible for each theme and discarding unintended ones. As for the Google search, the terms used included the theme name, adding “public” plus “attitude” terms, and the search results were screened thoroughly until the titles of the links showed redundancy in the upcoming search pages. After the identification of websites conveying multiple surveys, these were also used as a source to search for additional gray literature studies.

### Selection

**FIGURE 1:**
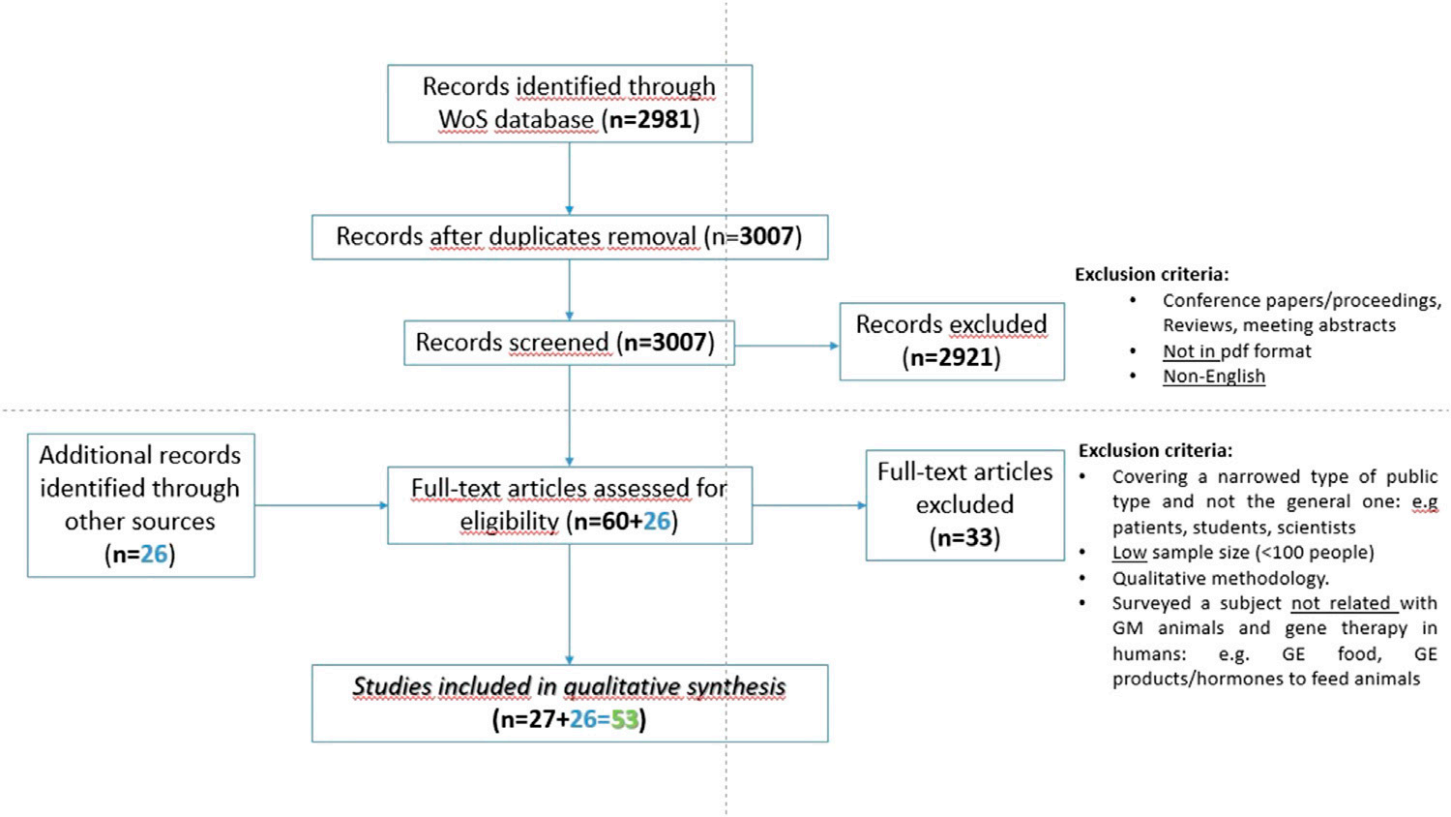
PRISMA flowchart and exclusion criteria used for the search and selection of primary publications in the systematic review.

The screening process is described in the PRISMA flowchart presented in Figure 1. All WOS publications that featured surveys with the general public regarding genetic modification of humans or animals were included in an Endnote library. All publications only addressing genetic modification of plants or crops were excluded from the library, and so were publications in the format of reviews and meeting or conference abstracts. All publications without access to its full-text or PDF document or not written in English were equally excluded. From the initial set of 2,981 publications, following duplicate removal and implementation of the exclusion criteria, 60 publications were left. After a careful reading of these, 33 publications reporting qualitative rather than quantitative studies and/or with low sample sizes (lower than 100 respondents) were excluded. To the WOS final list of 27 publications, 26 from the gray literature not meeting the exclusion criteria were added, equaling a total of 53 primary publications eligible for the systematic review.

### Survey parameters

The systematic review followed the PICOS guidelines (population, intervention, comparator, outcome, and study design) for the evaluation of studies, resulting from the initial search, except for the intervention index since we were not performing any statistical or meta-analysis (Centre for Reviews and Dissemination, 2009a; Centre for Reviews and Dissemination, 2009b). Population concerned the number of participants featured in the surveys and the country where the surveys took place. Comparison concerned the differences and similarities of public attitudes toward genetic modification procedures among citizens of different countries, comparison between years, and comparison of the type of questions and terminology used by surveyors. Outcomes analyzed were as follows: percentage of agreement with genetic modification in broad terms and for specific applications in humans and animals, the reasoning behind those attitudes, and respondents’ level of knowledge and/or the level of familiarity with biotechnology and/or genetic engineering topics. For more details, please see Supplementary Table S1.

### Critical appraisal of primary publications

All included primary publications were evaluated with regard to the methodological quality of the studies they reported. This was done by assessing if certain indicators were present or absent and by evaluating how well-described and appropriate they were for the studies in question (Supplementary Table S2).

The critical appraisal addressed the following: *content of questionnaires*—whether authors generated their own items or adapted them from previous surveys; *validity—*cross-checking between authors and/or external advisers and testing with the target population for both clarity and efficacy of measuring concepts; *reliability*—trustworthiness of the same items and constructs used within the surveys; *sampling*—representativeness and randomness; *risk of bias*—potential response, non-response, and selection bias; and *ethical practices*—details on informed consent obtained, if there were incentives given to respondents, and disclosure of any ethical statements by authors either related to ethical approval of studies or the potential conflict of interests experienced.

The search, selection, and first analysis were performed by the first author. Feedback was obtained by the other two authors. The critical appraisal was performed by PDR and IASO, while MSA performed the co-authorship network analysis (see Supplementary Material).

## Results

Of the 53 primary publications identified in this review, the 30 studies conducted prior to the advent of CRISPR-Cas9 technology in 2012 represent the pre-CRISPR period (Supplementary Tables S3, S4), whereas the 23 studies conducted from 2013 onward represent the CRISPR period (Supplementary Table S5). Pre-CRISPR studies were conducted between 1987 and 2010 and comprised 25 surveys with questions assessing attitudes toward the genetic modification of animals (GM animals) and 14 surveys assessing attitudes toward the genetic modification of humans. In the CRISPR period, eight survey studies addressed the genetic modification of animals, and 15 addressed the genetic modification of humans.

Generally speaking, the surveys conducted in the pre-CRISPR period focused on the opinion of the general public toward the genetic modification of animals for use in medical applications, food products derived from such animals (meat and milk), and the genetic modification of humans as gene therapy applications for the cure, prevention, and reduction of the risk of diseases. Some of these surveys also included additional aspects of human genetic modification, such as adults and children, prevention and therapy, and modification to change characteristics not related to diseases.

Table 1 summarizes the number of approvers of the genome editing technology in both periods in a proportion of 10 citizens, considering the previously mentioned applications and the region where the surveys took place.

**TABLE 1:** Number of approvers of the genetic modification of humans and animals in pre-CRISPR (1987–2012) and CRISPR (2013–2022) periods. The number of approvers in both periods is given for a total of 10 respondents for each primary publication included in the systematic review. Studies are listed according to their year of publication and include information about authors, country(ies) of survey administration, and the genetic modification of animals and humans’ features. For the pre-CRISPR period, studies with approvers of GM animals for transplants, meat, and milk in a total of 10 respondents and approvers of the genetic modification of humans for somatic and germline applications, and disease and enhancement settings are both represented. For the CRISPR period, studies with approvers of GE animals for transplants/medicines, milk, and welfare purposes in a total of 10 respondents and with approvers of GE humans for somatic and germline applications, and disease and enhancement settings are both represented.

			Approvers (in a total of 10 respondents)												
			Genetic modification of animals					Genetic modification of humans							
Authors (year)	Country(ies)		Transplants and/or medicines	Meat (pork, sheep, and cow)		Milk (cow and sheep)		Somatic (disease)		Somatic (enhancement)			Germline (disease)	Germline (enhancement)	
Office of Technology Assessment (1987)	US			7 (farm animals)				8 ***Prevent–8		5			***Prevent–8	4 (intelligence) and 4 (physical)	
								9–children					8 (non-fatal)		
Macer (1992)	Japan		-	-		-		5		-			-	-	
								7–children							
Macer et al. (1995)	Asia/Oceania							10 (TH), 9 (NZ, AU, and IS), 8 (J and RU), and 7 (IN) ***Prevent		More ethical			10 (TH), 9 (AU and IN), and 8 (NZ, J, RU, and IS)	Physical	
								8 (NZ, AU, IN, TH, RU, and IS) and 7 (J)		8 (TH), 6 (IN), 3 (NZ, AU, RU, and IS), and 2 (J)			Non-fatal	8 (TH) and 6 (IN)	
								Children					9 (TH), 8 (AU and NZ), 7 (RU and IS), and 6 (J and IN)	4 (RU), 3 (AU and J), and 2 (NZ and IS)	
								8 (AU, TH, IN, NZ, and IS), 7 (J), and 6 (RU)						Intelligence	
														7 (TH and IN), 3 (AU, J, and RU), and 2 (NZ and IS)	
Commission of the European Communities (1991)	EC12		9	4		4		7		-			-	-	
Commission of the European Community (1993)	EC12		-	4		4		7		-			-	-	
European Commission Directorate-General ScienceResearch and Development XII (1996)	EU15		4 - mice and pigs	-		-		-		-			-	-	
European Commission Directorate General for Research (2002)	EU15		4	-		-		-		-			-	-	
European Commission Directorate-General for Research (2006)	EU23		-	3		-		5		-			-	-	
Sato et al. (2006)	Japan		-	-		-		Opinion formation increased greatly after the first gene therapy success (only 56% formed an opinion)							
Barnett et al. (2007)	United Kingdom		-	-		-		Trust in the government and people in charge reveal favoring to allow gene therapy							
								Belief in public involvement, awareness, interest, and levels of education do not favor to allow gene therapy							
European Commission Directorate-General for Research (2010)	EU27		6	-		-		6		5			4	-	
Ng et al. (2000)	Japan		5 (mice)	5		4		7 ***Prevent - 6		-			7	5	
			3 (pigs)											3 (physical)	
														2 (intelligence)	
Macer et al. (2000)	Asia/Oceania		-	8 (TH), 7 (IN), 6 (AU and J), 5 (NZ), 4 (IS), and 3 (RU)		8 (TH), 7 (IN), 4 (NZ, AU, J, and IS), and 2 (RU)		10 (TH), 9 (NZ, AU, and IS), 8 (J and RU), and 7 (IN)		More ethical			10 (TH), 9 (AU and IN), and 8 (NZ, J, RU, and IS)	Physical	
								***Prevent		8 (TH), 6 (IN), 3 (NZ, AU, RU, and IS), and 2 (J)			Non-fatal	8 (TH), 6 (IN), 4 (RU), 3 (AU and J), 2 (NZ and IS)	
								8 (NZ, AU, IN, TH, RU, and IS) and 7 (J)					9 (TH), 8 (AU and NZ), 7 (RU and IS), and 6 (J and IN)	Intelligence	
														7 (TH and IN), 3 (RU, AU, and J), and 2 (NZ and IS)	
Evans et al. (2005)	Australia		-	-		-		-		-			4 (serious defect)	1 - cosmetic	
													3 (minor defect)		
													2 (aggression and violence)		
Cook AJ et al. (2004)	New Zealand		5	-		-		4		-			-	-	
Human Genetics Commission (2001)	United Kingdom		-	-		-		9		-			-	-	
								8–children							
Sturgis et al. (2005)	United Kingdom		-	-		-		9–cystic fibrosis, 8–heart disease, and 6–baldness		4–average height			6–8—cystic fibrosis and 5–6—heart disease	1–sex of the unborn baby	
								7–schizophrenia		2–height, intelligence, and sexual option			2–4—baldness		
								6–less aggressive *** Prevent							
								7–heart disease							
								2–baldness							
Marteau et al. (1995)	United Kingdom		-	-		-		2–aggressive behavior and alcoholism		1–adults (intelligence/specific skills)			-	-	
										1–children (appearance/behavior)					
Hampel et al. (2000)	Germany		4–laboratory: medical	2–farm: agricultural		-		7		-			-	-	
Norton et al. (1998)	Australia		-	3–sheep and pork		-		-		-			-	-	
Magnusson and Hursti (2002)	Sweden		-	2–pork and salmon		-		-		-			-	-	
Macer (1997)	Asia/Oceania		-	8 (TH), 7 (J and IN), 5 (AU and NZ), 4 (IS), and 3 (RU)		8 (TH and IN), 4 (J, AU, NZ, and IS), and 2 (RU)		-		-			-	-	
Macer and Ng (2000)	Japan		3	5		4		-		-			-	-	
Inaba and Macer (2003a)	Japan		5–mosquitoes	5		4		-		-			-	-	
Small et al. (2005)	New Zealand		2	-		2		-		-			-	-	
Nayga et al. (2006)	US and South Korea		-	3 (US)		-		-		-			-	-	
				2 (South Korea)											
Govindasamy et al. (2008)	South Korea		-	2		-		-		-			-	-	
Hallman et al. (2002)	US		8–sheep	3		8–sheep		-		-			-	-	
								
Hallman et al. (2003)	US		-	3		-		-		-			-	-	
Puduri et al. (2004)	US		-	3		-		-		-			-	-	
Macer et al. (1997)	Japan and New Zealand		Pigs	-		4 (J and NZ)		-		-			-	-	
			5 (J)												
			3 (NZ)												
			Mice												
			6 (J)												
			5 (NZ)												
Inaba and Macer (2003a)	Japan		3	-		-		-		-			-	-	
Chikhazhe (2015)	New Zealand		1	1		6		2		1			-	-	
McCaughey et al. (2016)	Global							6 (life-threatening and debilitating)		-			6 (life-threatening and debilitating)	3	
STAT and Harvard (2016)	US							-		-			3	1 (intelligence of physical)	
Funk, Kennedy, Sciupac (2016)	US							-		-			5 ***Prevent: 1–4	-	
Cormick and Mercer (2017)	Australia		5	3		-		7 (general)							
Chen and Liang (2018)	China							6		Intelligence: 2			6	-	
								Disease: 7–8		Skin color: 1					
								Non-disease (high cholesterol): 3							
Gaskell et al. (2017)	Europe							8		2			6	0	
								
Scheufele et al. (2017)	US		-	-		-		6		4			6	3	
Weisberg et al. (2017)	US		-	-		-		Risk−7; no risk–8							
Wang J-H et al. (2017)	China		-	-		-		8–adults and children		4			6	4	
van Mill et al. (2017)	United Kingdom		7 (mosquitoes and organs)	5–efficiency of food		7–resistant to disease		8–(in)curable		5–prolonged life			8	-	
				3–profit		6–invasive species		7–non-life-threatening		2–cosmetic					
						5–control pest and hornless cows		6–disorder not inherited		3–intelligence					
Hendriks S et al. (2018)	Netherlands		-	-		-		9		-			7–Neuromuscular	2	
													3–HIV		
Uchiyama et al. (2018)	Japan		-	-		-		The higher the awareness, the higher the support							
Lakomý M et al. (2018)	Europe		-	3–6		-		8–9 (disease) ***Prevent		-			-	3–5	
								8–9 (disease)							
								7–9 (disabilities)							
Pew Research Center (2018)	US							-		-			7 (treatment of serious illnesses) ***Prevent - 6	2 (intelligence)	
Funk and Heferon (2018a)	US		6 (transplants)	4		-									
			7 (mosquitoes)												
McCaughey et al. (2019)	Global							6 (life-threatening and debilitating)		-			6 (life-threatening and debilitating)	3	
Critchley et al. (2019)	Australia		7	6		-		***Prevent - 8		5			***Prevent - 8	4	
McConnachie et al. (2019)	US		-	6		9									
Yunes et al. (2019)	Brazil		-	4		3									
Kohl et al. (2019)	US					Wildlife - 1									

### Genetically modified animals in pre-CRISPR and CRISPR periods

A) Pre-CRISPR: GM animals for food purposes are mostly rejected, and medical applications are seen ambivalently worldwide.

**FIGURE 2:**
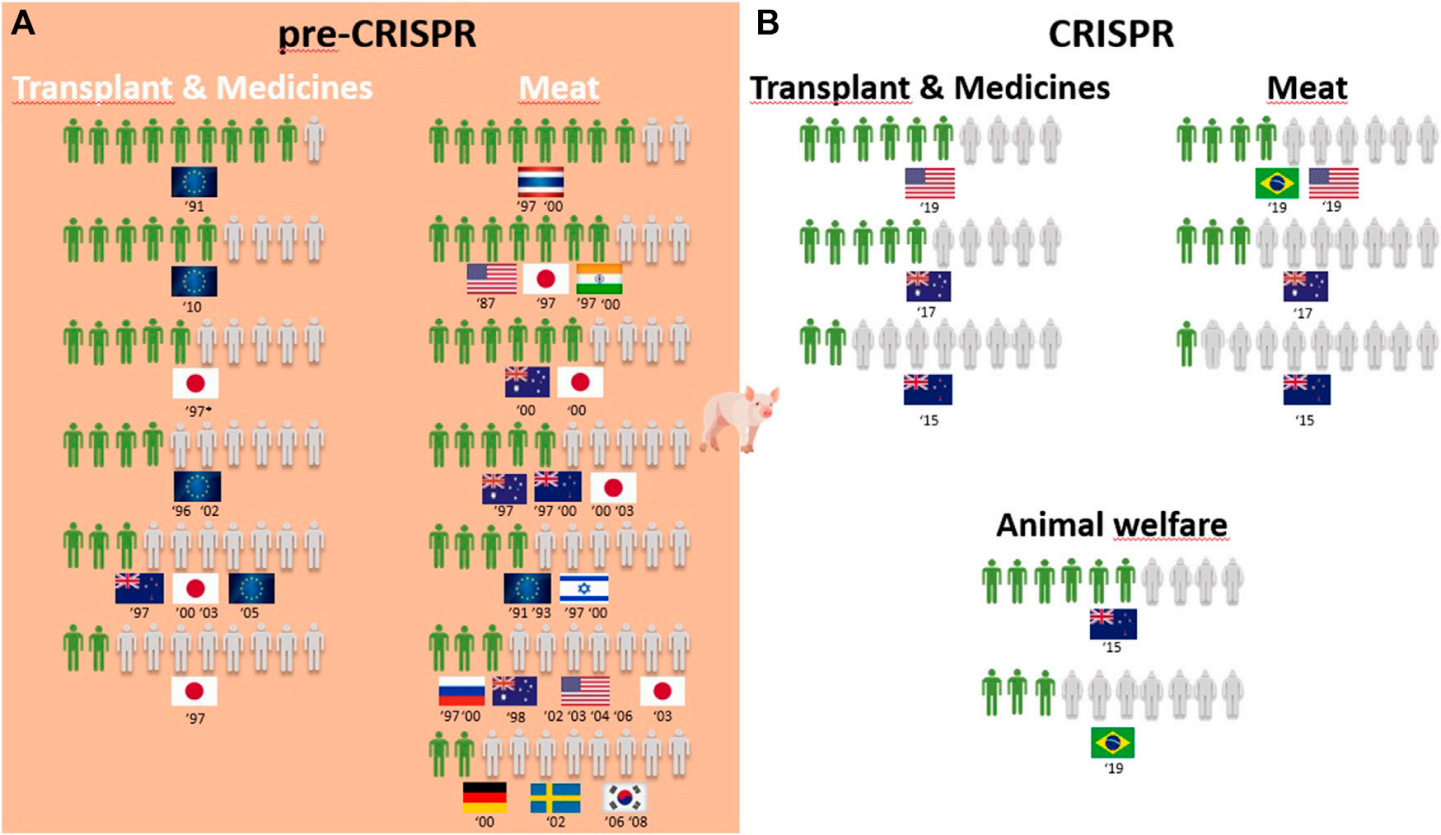
Public support for gene modification in pigs worldwide for a proportion of 10 citizens upon survey inquiry in pre-CRISPR **(A)** and CRISPR **(B)** periods. **(B)** CRISPR: Animal welfare in focus and genome-edited animals for food applications continue to be less approved than for medical applications.

**FIGURE 3:**
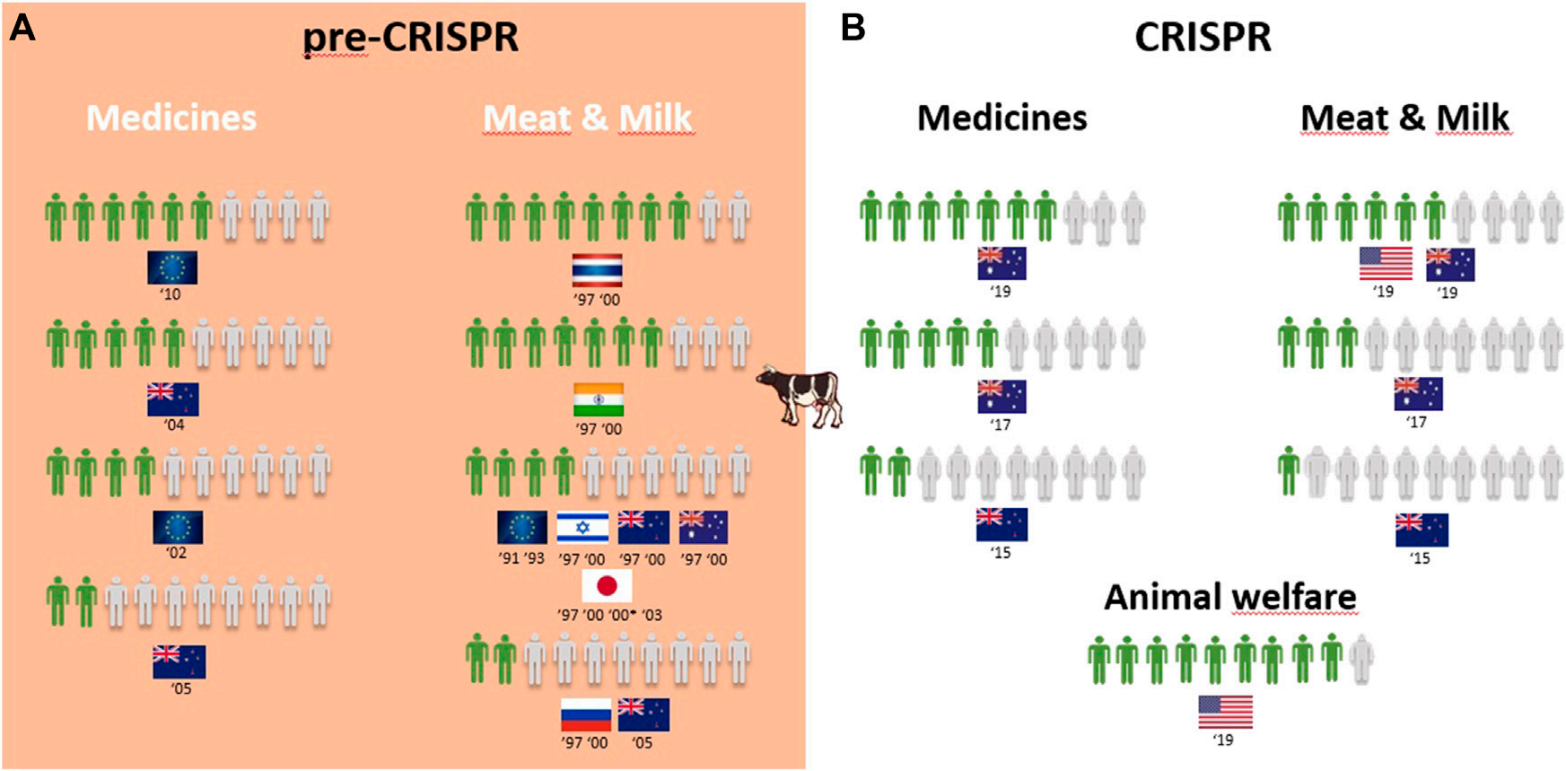
Public support for gene modification in cows worldwide for a proportion of 10 citizens upon survey inquiry in pre-CRISPR **(A)** and CRISPR **(B)** periods.

Overall, 25 of the 30 surveys from the pre-CRISPR period covered the genetic modification of animals (GM animals). In a quick overview of Table 1, we can see that transplants and medicines face a higher approval from respondents than food products derived from GM animals. For all cases of food derived from GM animals, either to obtain “leaner meat,” “meat less fatty,” or simply “meat from these animals,” the approval rate is very low among respondents in almost all countries analyzed, and this trend is consistent from 1987 to 2006, although there are some studies where approval for meat consumption of GM animals reaches more than half of the respondents (the US in 1987, Japan in 1997, Thailand and India in 1997 and 2000, and Australia in 2000; Figure 2A). Approval of GM animals for organ transplantation and medicines dropped considerably between 1991 and 2010 in Europe. The lowest approval reached 4 in every 10 European citizens in 1996 and 2002 and only 3 in every 10 citizens in 2005, according to Eurobarometer (Figure 2A). Conversely, medicines derived from GM cows gained approval among Europeans between 2002 and 2010, according to Eurobarometer (Figure 3A). Australians and New Zealanders are among the lowest approvers of GM animals worldwide for both medical and food purposes, and their approval has been decreasing in surveys after the 2000s (Figures 2A, 3A). A similar trend is seen for citizens from the US who rejected meat derived from GM pigs in all surveys conducted after 2000 (Figure 2A). Japanese citizens were the most surveyed public in the pre-CRISPR period, regarding attitudes toward GM animals, which they approved slightly more for food—meat and milk—than for medical purposes (organs for transplantation in pigs (Figure 2A) and mice for cancer research (Supplementary Figure S5), going against the general trend. A note of remark is their decrease in approval for the meat of GM pigs from 1997 to 2003, as well as for transplants and medicines (Figure 2A). The use of GM mice for cancer research is seen as “to be encouraged” more than GM pigs for transplants among Japanese citizens (Table 1–pre-CRISPR). In two studies of single European countries, in Germany, less than half of the citizens supported GM laboratory animals for cancer research, and Swedish citizens categorically rejected GM salmon for food consumption (Supplementary Figure S5), similar to their choice regarding GM pigs (Figure 2A).

The CRISPR period surveys on attitudes toward GM animals represent a total of eight surveys worldwide over a 10-year period, with the highest number conducted in the US (Funk and Heferon, 2018a; Kohl et al., 2019; Lull et al., 2019; McConnachie et al., 2019). Table 1 (CRISPR period) shows that US citizens approve of the genetic modification of animals for human health purposes, in this case, genome-edited pigs for transplants of organs to humans (6 in 10) and genome-edited mosquitoes to eradicate the spreading of diseases into humans (7 in 10). Upon examining Oceanic countries, Australian citizens are more supportive of GE cows for medicines than for meat- and milk-derived products, while New Zealanders are profound rejecters of GE animals for both applications (Figure 3B). Regarding the approval for genome-edited pigs for food consumption, Brazilian citizens are mostly rejecters (only 4 in 10) in contrast to US citizens, where more than half support gene editing either for derived products such as meat from GE pigs or meat and milk from GE cows (Figures 2B, 3B). A new type of question present in surveys from the CRISPR era deals with the genetic engineering of animals for improved animal welfare. Here, we can see that US citizens frankly approve of “GE cows to become hornless” as a way to avoid invasive and painful dehorning (Figure 3B). The majority of citizens in New Zealand approve of GE pigs for better animal health and safety, whereas among Brazilian citizens, the approval for GE pigs to “reduce boar taint in pigs” (as an alternative to invasive and painful castration) is below half of the respondents (Figure 2B). The only study covering genome editing in wildlife reported a profound rejection among US citizens (Table 1; Supplementary Figure S5–CRISPR period) because this was perceived as a risk for both humans and nature.

### Genetic modification of humans in pre-CRISPR and CRISPR periods

A) CRISPR: Somatic genetic modification for therapy is a yes, while enhancement is a no.

**FIGURE 4:**
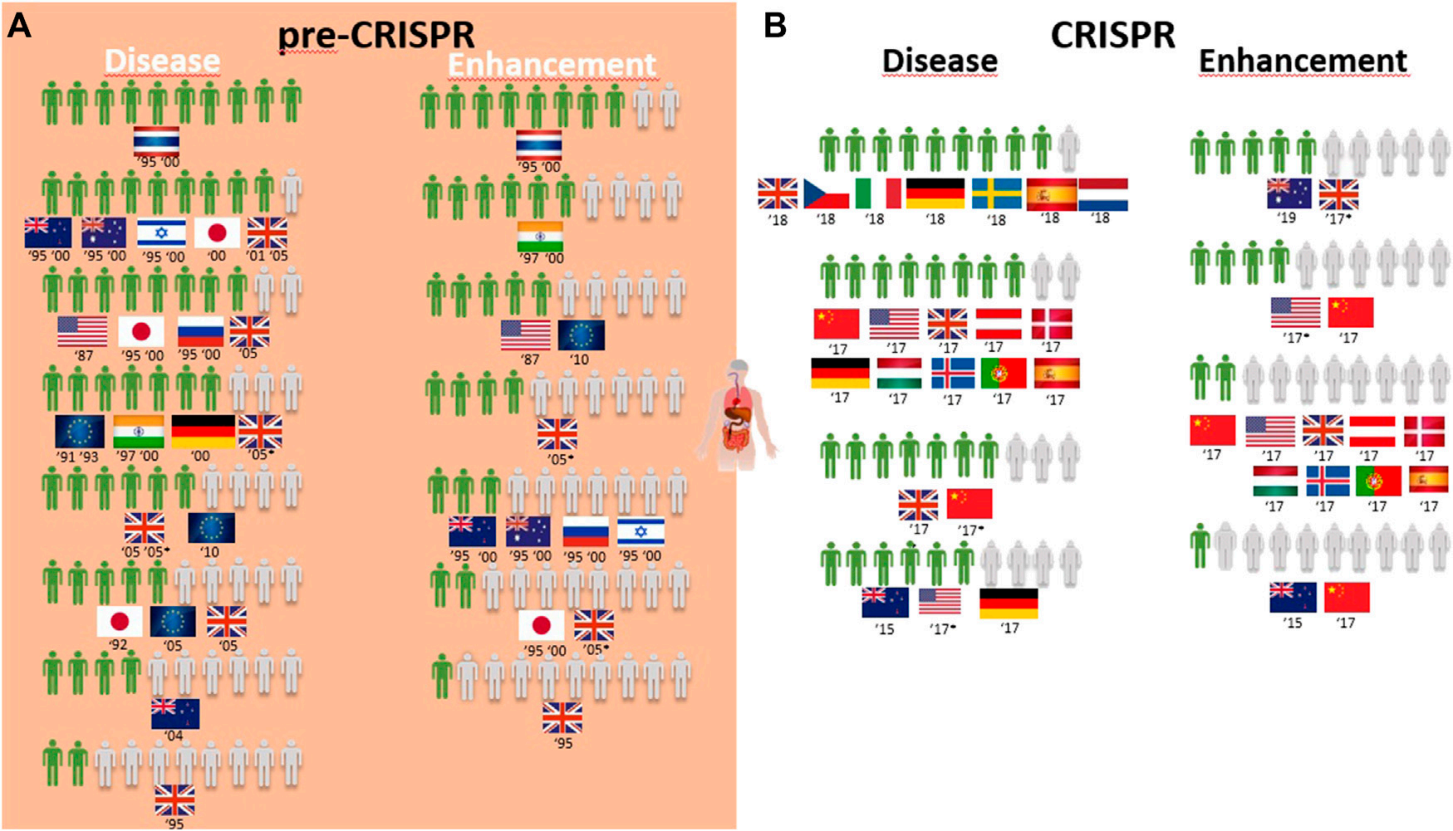
Public support for gene modification in human adults worldwide for a proportion of 10 citizens upon survey inquiry in pre-CRISPR **(A)** and CRISPR **(B)** periods.

Overall, the genetic modification of humans for gene therapy purposes receives medium to high acceptance worldwide (Table 1; Figure 4A). Only three exceptions can be identified: two related to disease prevention, where 4 in every 10 New Zealand respondents agree with it for “preventing stomach cancer by modifying a person’s genetic code,” and 2 in every 10 United Kingdom citizens approve it to prevent baldness (Table 1). The same low proportion of United Kingdom citizens approved of gene therapy to treat aggressive behavior and alcoholism identified as diseases (Figure 4A). The overall greatest support for gene therapy is found among Thai citizens, followed by Australians, New Zealanders, and Israeli and Japanese citizens in the 1990s to cure fatal diseases and United Kingdom citizens in the 2000s for genetic diseases like cystic fibrosis and heart diseases (Table 1; Figure 4A). On the other side of the genetic modification of humans, enhancement is mostly rejected by all citizens surveyed during the pre-CRISPR period, with the only exceptions being in 1995 and 2000 studies, where Thai and Indian citizens show high approval to “make people more ethical” and the ambivalence demonstrated by US citizens in 1987 toward “changing the genetic makeup of human cells” as well as European Union respondents in 2010 regarding human enhancement (Table 1; Figure 4A).

**FIGURE 5:**
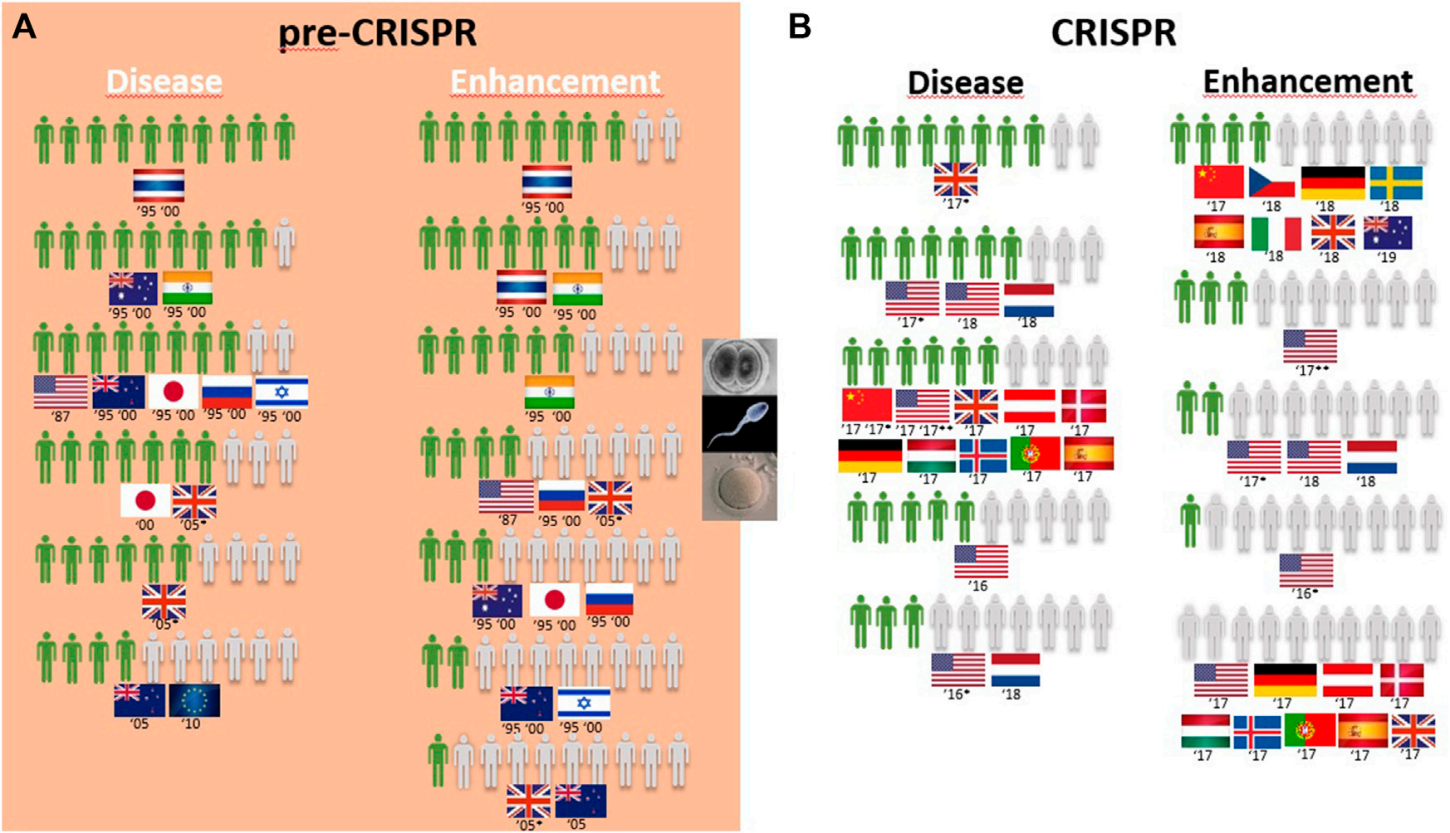
Public support for gene modification in human germline cells worldwide for a proportion of 10 citizens upon survey inquiry in pre-CRISPR **(A)** and CRISPR **(B)** periods.

**FIGURE 6:**
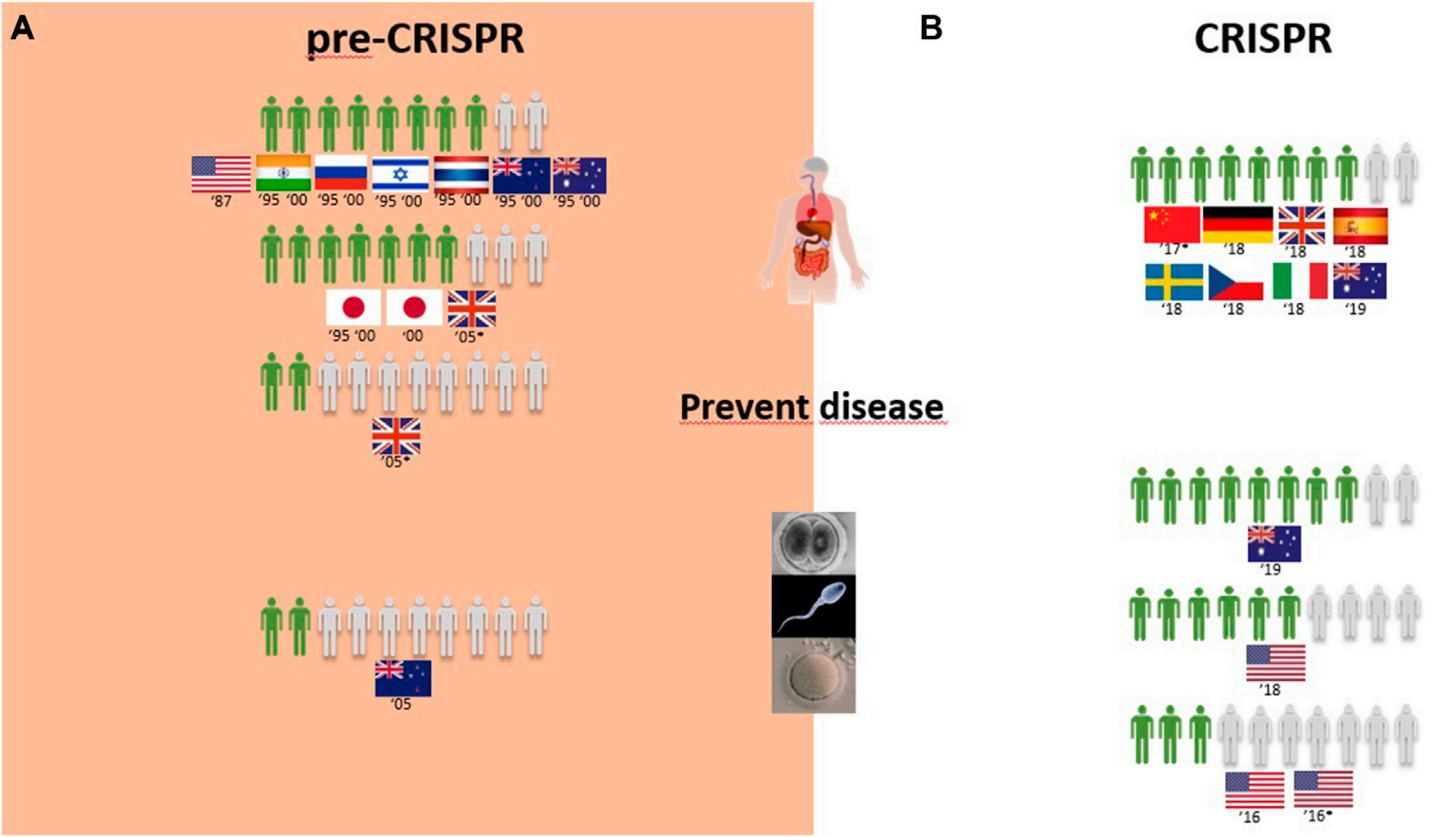
Public support for gene modification in human adults and human germline cells for preventing disease worldwide for a proportion of 10 citizens upon survey inquiry in pre-CRISPR **(A)** and CRISPR **(B)** periods.

Germline genetic modification for therapy purposes gained high approval, similar to somatic genetic modification. Once again, there are exceptions, and these involve citizens from New Zealand in 2005 and Europeans in 2010. For New Zealanders, this represents a drop from much higher levels in the second half of the 1990s (almost 8 in every 10 citizens supporting it to cure fatal disease (Figure 5A); then, 10 years later, the number decreased to 4 in 10 citizens for approving GE for serious defects and further decreased to 2 for minor defects and to 1 in every 10 citizens for preventing aggression and violence (Table 1; Figure 6A)). Among the most approving respondents of the germline genome modification for therapy are Thai respondents, followed closely by Australian and Indian citizens (Figure 5A).

Germline genetic modification for enhancement purposes is approved largely by Thai and Indian citizens to improve the physical characteristics and intelligence level “that children would inherit” (Table 1; Figure 5A). All the other countries surveyed about this rejection of those applications, particularly for the improvement of intelligence, cosmetic modifications in children, and determination of sex in an unborn baby (Table 1).

B) CRISPR: Genome editing of humans for therapy is considered more acceptable in somatic than in germline modifications, but enhancement is opposed.

Surveys in the CRISPR period inquired citizens about genome editing of humans for therapy, similar to that in the pre-CRISPR period, with results showing strong approval worldwide. At this point, Europeans are the most approving of GE to cure diseases, although by a low margin when compared with Chinese and US citizens and with New Zealand citizens following closely. For the prevention of diseases, all citizens surveyed demonstrate an equally high approval rate of 8 in every 10 citizens (Figure 6B). In children, the approval rate of gene therapy was only assessed in China and showed to be similarly high among citizens (Table 1).

Similar to the pre-CRISPR period, genetic enhancement of human beings was generally rejected by citizens worldwide (Figures 4A, B). Intelligence and the change in skin color were purposes profoundly rejected by Chinese citizens (Table 1). The only case with less than a majority rejecting enhancement (genome editing of “human body cells to change one’s appearance”) was among Australians and to “prolong life” among United Kingdom citizens (Figure 4B).

Overall, GE in the human germline, as in the cases of unborn babies and embryos to cure serious diseases, gained approval among citizens (Figure 5B). US citizens were the most surveyed public in the CRISPR period, and multiple surveys conducted consecutively from 2016 to 2018 demonstrate a growth in approval of this type of genetic intervention for disease during this period, increasing from 3 to 5 in every 10 citizens in two surveys conducted in 2016 to 6 and 7 in every 10 citizens in surveys conducted in 2017 and 2018, respectively. The remaining studies include European and Chinese publics, and approval rates fall between the highest and the lowest of the US studies (Figure 5B; Table 1). In fact, 7 in every 10 citizens from the Netherlands approve GGE to avoid hereditary neuromuscular disease, while only 3 in every 10 citizens agree with it for HIV resistance (Table 1). Australian citizens are the most approving of GE in germ cells and embryos, whereas US citizens have lower approval rates, between 3 in every 10 citizens in 2016 and 5 in every 10 citizens in 2018 that approve genetic interventions in unborn babies (Figure 5B). Likewise, this is similar for “prevention of disease” scenarios (Figure 6B).

Finally, the idea of genetic enhancement of unborn babies is not approved by members of the public anywhere in the world. It was even completely rejected among Europeans and US citizens in a survey conducted in 2017 (Figure 5B), and although the surveys conducted demonstrate a higher approval rate among Europeans 1 year later, still less than half of the participants agree with germline genetic enhancement, which is similar to the responses of Australian and Chinese citizens (Figure 5B; Table 1).

### Awareness and knowledge correlation with public attitudes toward the genetic modification of humans and animals

Overall, the more aware or knowledgeable inquired publics are about topics of science and technology, in general, biotechnology, genetics, genetic modification, and gene editing, the most approving they are of genetic modification in humans and animals. In total, 44 surveys assessed the awareness and knowledge of participants about genetic modification topics, and from these, 17 surveys assessed only awareness (level of familiarity) and eight surveys assessed only knowledge (the level of education of respondents) about these topics.

From the total of surveys administered during the pre-CRISPR period, almost none showed a significant correlation between the awareness and knowledge of citizens about topics of science and their approval of genetic modification of humans or animals (OTA, 1987; Macer, 1992; Ng et al., 2000; Hallman et al., 2002; Nayga et al., 2006). There was, however, in the United Kingdom in 2007, a survey that demonstrated a significant correlation between higher awareness of citizens to genes and genetics and lower approval of gene therapy in humans (Barnett et al., 2007). Surveys measuring awareness and knowledge of genetics demonstrated a tendency or association between approval of gene therapy in humans and higher awareness or knowledge of these topics (Macer et al., 1995; Sturgis et al., 2005; Sato et al., 2006). Curiously, a tendency for citizens to reject GM animals when they are more aware of the technology can be observed (Macer et al., 1997; Ng et al., 2000; Inaba and Macer, 2003a; Inaba and Macer, 2003b). In terms of knowledge, the attitude of citizens showed a tendency to approve when the knowledge was higher (OTA, 1987; Commission of the European Communities, 1991; Commission of the European Community, 1993; European Commission Directorate-General ScienceResearch and Development XII, 1996; Hallman et al., 2002; Hallman et al., 2003; Puduri et al., 2005), except for one study with German respondents (Hampel et al., 2000).

In the CRISPR period, no studies demonstrated a significant correlation between the approval of GE animals or GE humans and awareness or knowledge about the topics among citizens. Nevertheless, there was a tendency for citizens who were more aware of scientific topics to show increased acceptance of gene therapy in humans and GE animals (Funk et al., 2016; STAT and Harvard, 2016; Scheufele et al., 2017; Funk and Heferon, 2018b; Funk and Heferon, 2018a; Lakomý et al., 2018; Uchiyama et al., 2018; Critchley et al., 2019; Lull et al., 2019; Chikhazhe 2015; McConnachie et al., 2019), except for one study finding no correlation (Yunes et al., 2019).

## Methodological quality: reporting of critical issues

This section presents the results of critical appraisal of the methodology, as reported in the primary publications selected for analysis, to provide an indication of the methodological quality (Petticrew and Roberts, 2005). All data are summarized in Supplementary Table S2.

### Questionnaire development

Surveys may be the result of original item generation or adaptation of items used in previous surveys. For pre-CRISPR surveys, there was an approximately even distribution between the eight studies originally generating their own items and the 10 that adapted existing studies. For studies in the CRISPR period, generating own items was much more common (11 *versus* 4). As for the remaining 18 surveys that have information available both in the pre-CRISPR and CRISPR periods, a hybrid approach was followed.

The validity of a survey instrument is reflected by how well it measures what it is supposed to measure. Face validity (whether it appears to measure what it should) and content validity (if it is understandable to respondents) were the most reported types in 29 and 37 studies, respectively (Boateng et al., 2018; Hair et al., 2019). Construct validity, to check if the construct used is suitable, appears mostly in the form of hypothesis testing in 16 pre-CRISPR and 14 CRISPR surveys.

Reliability is about how reproducible survey instrument data are across different applications of the survey. Most papers (32 of the 53) included in the review did not report this parameter. Among the papers that did, the most used was Cronbach’s alpha index to measure internal consistency and split-half reliability, where samples are divided into halves or thirds to ensure that there is not a significant difference between groups of individuals studied.

### Sampling: method, response rate, and weighing

The methodology of sampling participants for surveys is very diverse across the different surveys analyzed. CRISPR surveys were conducted mostly online, and pre-CRISPR surveys overlap between telephone, face-to-face, and mail responses. Quota sampling from databases (rather than random sampling) was more common in CRISPR surveys than in pre-CRISPR surveys (10 vs. 3). Weighing of the sample was used to overcome potential sampling bias but was reported in less than half of the studies. In the studies where weighing was reported, the correction tool mostly used was based on demographics for surveys in both time periods. The majority of the studies report a medium response rate (25%–75% of invited participants responded). CRISPR studies show a medium to high response rate compared with pre-CRISPR studies, for which response rates were generally low to medium. Multinational surveys such as Eurobarometer and intercontinental surveys demonstrate a different response rate per country, and therefore, sample weighing was used. Furthermore, an equal number of pre-CRISPR and CRISPR surveys did not report on the response rate (7 each).

### Methodology accountability and reporting

Only half of the studies provide information on bias, and this is transversal to both pre-CRISPR and CRISPR studies. The most commonly referred by authors in the studies from the systematic review is recruitment bias, with under- or over-representation of certain demographic groups for education, age, gender, race, and socio-economic status. Some studies report techniques to avoid bias, namely, the use of random digit dialing to avoid inadequate telephone surveys (OTA, 1987), demographic comparisons to the census to avoid sample distortions (OTA, 1987), standardization of questionnaires and their delivery (Macer et al., 1995), use of open responses (Macer et al., 1995; Macer et al., 2000), background campaigning (McCaughey et al., 2019), online survey to have a more robust sample (Weisberg et al., 2017), online tools to avoid age bias (Wang et al., 2017), and not mentioning the survey nature to avoid self-selection bias (McConnachie et al., 2019; Yunes et al., 2019). In CRISPR studies, authors report about ethical practices taken during survey conduction, whereas this is mostly not reported in pre-CRISPR studies. Such practices involve obtaining informed consent from participants, voluntary participation invitation, obtaining a privacy statement, or even the chance of withdrawal from the study. Formal ethics approval for the study was only reported for 10 studies from the total of publications in the systematic review. Finally, incentives to participants in order to increase their willingness to participate were disclosed in nine studies.

## Discussion

This systematic review of 53 primary publications on attitudes toward genetic modification in humans and non-human animals provides a comprehensive picture of studies in Europe, North America, Asia, and Oceania over 35 years. The review shows some variation between countries but a clear pattern in how different applications are viewed, which does not change substantially over time.

There is an overall positive attitude to gene therapy for medical purposes in humans, both for adults and children, and both as treatment for a fatal genetic disease and as prevention from developing a disease that would otherwise be likely to occur. This is transversal from the early studies before the 1990s to the most recent studies, with little variation among the public and regardless of their origin. This is in agreement with international and national policies (Walters, 1991; Horst, 2007; DH-Bio, Committee on Bioethics, 2015; Polcz and Lewis, 2016; Nicol et al., 2017), and indeed, several clinical trials of somatic gene therapy are underway (EASAC, 2017; Karagyaur et al., 2019). Key challenges in the use of these therapies in the clinic raised by scholars regard their definition and regulation (Nicol et al., 2017; Sherkow et al., 2018) and were partly recognized in some of the public opinion surveys, including the “need for strict regulation” in somatic therapy (Eurobarometer, 2010) and the need for FDA approval to proceed (STAT and Harvard, 2016).

The differentiation between germline and somatic cells becomes important over time. Surveys administered pre-CRISPR hardly ever distinguish between the correction of genes carrying disease for the individual and those that can be passed onto future generations. In contrast, post-CRISPR surveys address this directly not just by questioning explicitly about germline and unborn babies but also when asking both about germline *versus* somatic therapy and adult *versus* prenatal therapy. Overall, somatic gene therapy is widely accepted in most surveys, whereas there is much ambivalence about germline gene therapy, with higher support to prevent future health issues in unborn babies and lower support if the purposes are non-health-related issues like physical and psychological characteristics. The ethics of germline gene editing experienced a spike of interest with the advent of the CRISPR-Cas9 technology (National Academy of Sciences, 2017; Nordberg et al., 2018; Brokowski and Adli, 2019; Morrison and de Saille, 2019), and the ethical issues are discussed by the general public and the scientific community in distinct ways. The surveyed public often mentions unnaturalness, messing with nature, and humans playing God in the creation of designer babies as main arguments to reject germline gene editing and health benefits as a reason to accept it. Researchers, on the other hand, primarily refer to technical hurdles and uncertainties, such as off-target effects and mosaicism, as the background of ethical questions related to unintended consequences and safety and also the problem of introducing irreversible changes to the genome of future individuals whose consent cannot be obtained (Bosley et al., 2015; Gyngell et al., 2017; National Academy of Sciences, 2017; Brokowski and Adli, 2019; Morrison and de Saille, 2019). Many scholars defend that, while germline gene editing will eventually be inevitable, the technology should not be pursued in the clinic except when no other alternative exists to prevent a severe or deadly genetically transmitted disease and only after the technology has proven to be safe to proceed to clinical trials (Bosley et al., 2015; Gyngell et al., 2017; Brokowski, 2018; Browkoski and Adli, 2019; Morrison and de Saille, 2019). Others argue that research on gene editing could improve the understanding of genetic diseases and should be used for single-gene disorders and other disorders arising from polygenic traits (Gyngell et al., 2017). Scholars have defended the adoption of a moratorium on germline gene editing more than once: following the first edit on human cells and after the birth of the first gene-edited babies in late November 2018, respectively (Baltimore et al., 2015; EASAC, 2017; Brokowski and Adli, 2019; Lander, 2019), often justified by the precautionary principle and taking into account the unpredictability of an emerging new form of technology (Nordberg et al., 2018).

A third relevant point is the differentiation between therapy and enhancement. Across countries, citizens are generally opposed to genetic modification for the purpose of enhancement. When asked to distinguish between different types of enhancement, intelligence or psychological features were favored over physical abilities and appearance in US and British studies. Across the countries where there is some support for non-therapeutic gene editing, the most supported purpose is improved human health. This is in line with the establishment of a purpose for genome editing beforehand and the clear distinction between what is a disease and what is a deviation from a societal norm (Brokowski and Adli, 2019). As for current guidelines, the US National Academies of Sciences, Engineering, and Medicine exclude the use of genome modification for any type of enhancement under any circumstance (National Academy of Sciences, 2017; Brokowski and Adli, 2019). The reasons for this are also aligned with the slippery-slope argument that gene editing will ultimately lead to social harm by the creation of new genetically modified humans that may lead to “new forms of inequality, discrimination, and societal conflict” if regulation fails to limit germline gene editing to therapeutic uses (Gyngell et al., 2017).

With regard to GM animals, the aspect that stands out as a continued trend is the way acceptance differs between different purposes. Overall, GM animals appear as generally not acceptable for food purposes, be it for leaner or healthier meat, as in the case of GM pigs, or to produce more milk, as in the case of GM cows. In 2007, Novoselova et al. highlighted the important role of consumers in the potential integration of GM products derived from animals into the food chain, pointing out the perception of healthy and safe food, as well as understanding of environmental and ethical concerns as key issues (Novoselova et al., 2007). This perception is based on arguments that “genetic modification is intrinsically wrong” for food applications (Frewer, 2003), with many people even questioning the usefulness of such applications (Macnaghten, 2004). Risk and benefit perceptions regarding food are affected by many factors which interact in complex ways; specifically, with regard to animals, this is further complicated by the duality of the animal as a friend and food (Ueland et al., 2012). As for GM pigs or GM sheep, for medical purposes such as organs for transplantation and derived products to help with diseases, the acceptance is higher. Furthermore, among professionals who are involved with animal research, support for GM pigs in medical applications like xenotransplantation was greater than that for food applications (Schuppli and Weary, 2010). Although this would overcome the shortage of human organs for transplantation, this discussion is again reflecting current and older moral reservations regarding the mixing of tissues from human and non-human species, as well as the unnaturalness and invasiveness of the process and ultimately the risk for human health (Einsieddel, 2005; EASAC, 2017; Luna, 2017; de Graeff et al., 2019). Similarly, it has been found over the years of public opinion surveys that public perceptions of risk are higher when they concern GM animals rather than GM crops/microorganisms and are also perceived as riskier and having more ethical concerns if the context is food applications rather than medical applications as the latter tend to be evaluated on a more specific or case-by-case basis (Frewer, 2003; Frewer et al., 2011). The two differences that appear when comparing surveys from before and after the introduction of CRISPR-Cas9 technology are largely associated with the type of questions that were asked. In pre-CRISPR surveys, most respondents see laboratory research in animal models like GM mice as useful but not morally acceptable. This reflects an ambivalence between what is perceived to be a valuable objective (the study of human disease) and the concerns over animals’ welfare (Spencer, 1999; EASAC, 2017; de Graeff et al., 2019). In the CRISPR surveys that include animal applications, the questions are about applications where genetic modification is done to avoid animal welfare problems, and while people mention some concerns, in particular about potential suffering, overall, they see it as something good. However, they also reveal an unwillingness to consume products derived from these animals, similar to respondents in pre-CRISPR surveys. This follows the usual perception of risks and ethical concerns where the public has also been found to be willing to pay less for GM foods than conventional ones (Frewer et al., 2011). Impacts on human health by the introduction of genetically modified species in the food chain, unnaturalness, and potential ecosystem disturbance are also recognized as moral issues of these interventions (Nuffield Council of Bioethics, 2016; EASAC, 2017; Nordberg et al., 2018; de Graeff et al., 2019). Impacts on biodiversity and sustainability are repeatedly identified ethical concerns about the genetic modification of animals, together with animal welfare, tampering with nature, and unnaturalness (Frewer et al., 2004; MacNaghten, 2004; Schuppli and Weary, 2010; Frewer et al., 2011). Furthermore, GM animals are also seen more negatively than GM plants, and the perception that the technology is unnatural has increased over the years (Frewer, 2017).

Across many surveys, there is a correlation with support for gene technology: the higher the awareness and knowledge levels, the higher the support as well. This lends some support for the deficit model, according to which education and an improved public understanding of science would lead to a higher acceptance of food that is genetically engineered and gene therapy as a clinical treatment approach (Uzogara, 2000; Gottweis, 2002). However, in most cases, this relationship is weak, and awareness and knowledge levels toward genetic engineering or modification and biotechnology are generally not considered predictive of public attitude (Priest, 2000; Gottweis, 2002; Chen and Chern, 2004; Saher et al., 2006; Wheeler, 2008; Frewer et al., 2011). In this context, it is relevant to consider the role of social media. Huber et al. (2019) found that the use of social media news and trust in science was positively correlated across data from 20 countries. They also found that trust in science was more strongly related to social media news use than traditional media news. However, an important caveat highlighted by the authors is that their analysis did not consider the quality of the information. The social media discussion of COVID-19 has made the question of whether what is disseminated is verified scientific information or misinformation/fake news increasingly critical. Radrizzani et al. (2023) surveyed a sample of the United Kingdom public about how their trust in science had been affected by the introduction of the first COVID-19 vaccines. They found that it was much more common for people to report that not only their trust had increased than that it had decreased but also that trust decreased among those who had little trust in science to begin with. In the US, Xiao et al. (2021) found that individuals who get most news from social media had greater beliefs in conspiracies in general and in COVID-19-related conspiracies in particular. Social media may also play a different role in survey research, as illustrated by studies covered by our systematic review, such as McCaughey et al., 2016, Wang et al., 2017; McCaughey et al., 2019, that included online social media as a method for participant recruitment and response to surveys.

The critical appraisal of methodological quality shows that most studies provide low- to medium-quality information. Only two publications (Magnusson and Hursti, 2002; Kohl et al., 2019) fulfill all the criteria recommended for questionnaire surveys (Petticrew and Roberts, 2005; Malhotra, 2006; Stockemer, 2019a; Stockemer, 2019b). Most studies report or demonstrate the consideration of two to three of the criteria but typically not on the aspects considered more relevant for ensuring the methodological quality, such as the item generation method and response rate. Characteristics of greater relevance, such as validity, reliability, risk of bias, and sampling, are reported at a much lower frequency than what is desired. Poor methodological quality may justify the exclusion of studies from a systematic review. We nevertheless included all surveys in this systematic review because, first, our priority was comprehensiveness and, second, in order to be able to highlight the issue of study quality, which is not yet receiving as much attention in reviews of social science research as it does in biomedical research. Although not reporting does not necessarily mean that the practice was absent, it does, at least, suggest limited attention to the methodology. Lack of information is more common in earlier studies, which probably reflects the changing practice in the field. One also needs to distinguish between survey reports in the gray literature, which focus on reporting the results, from articles in scholarly journals with peer review, where a discussion of methods and issues such as the risk of bias are expected to be an integral part of reporting. Finally, the lack of information regarding formal ethics approval might simply mean that the context in which the study was implemented was considered exempt from formal approval, even though mentioning the exemption would be expected.

To the best of our knowledge, our study is unique in comprehensiveness. First, it includes publications covering almost 35 years and addressing attitudes to human and non-human genetic modifications. Although the 2020 systematic review by Delhove et al. undertook a similar approach in terms of timespan and definition of primary publications, it covers only attitudes to human genetic modification (Delhove et al., 2020). The limitations to our study include the choice of databases, studies, and information to include. We used WOS as the source database and Google web search for publication retrieval. It is possible that other databases would have generated a somewhat different outcome in terms of selected publications. We chose only to include studies of the general public, excluding studies of only specific publics (Frewer et al., 1997; Chen and Chern, 2004; Napier et al., 2004). Additionally, we must admit some delay regarding the change in terminology from “genetic modification” to “genome editing” that occurred with the advent of CRISPR in 2012 and which was considered in our literature search (see Methodology). In terms of analysis of results, we opted to only assess the influence of awareness and knowledge in public attitudes and did not include other parameters that could have had an influence here, like trust in organizations, demographics (e.g., socio–economic status), and religious index. The reason to only include awareness and knowledge is because these variables have been continuously assessed, and therefore, we could have a parallel view of how they would have influenced public opinions toward genetic modification over time. Finally, the present paper includes only a qualitative analysis of quantitative results, and we did not perform a meta-analysis.

## Future perspectives

Public consultation is critical in controversial matters in relation to genetics and biotechnology, especially when applications will potentially directly influence citizens’ lives and, therefore, have to ensure accurate representation (Halpern et al., 2019). Although cross-sectional surveys such as those we analyzed are important because they provide an overview of how public opinion evolved during the last 35 years, real comprehensive initiatives of public engagement and societal debate on genome modification beforehand are indispensable (Tait et al., 2017; Jasanoff and Hurlbut, 2018; Wirz et al., 2020). This could include a citizen policy approach, such as that described for climate action policy (Wintle et al., 2017; O’Grady, 2020). This would be particularly important in the context of policy-making for CRISPR-Cas9 technology implementation. The design of citizen engagement initiatives with multiple stakeholders in the discussion of genome editing driven by the intervention of some associations already in place like the Association for Responsible Research and Innovation in Genome Editing (ARRIGE) may elevate the dialog and contribute to the adoption of a participatory governance framework that may resemble such reflections (Montoliu et al., 2018; Hirsch et al., 2019; Pereira and Völker, 2020). This path would also entail the best opportunity for scientists and policymakers to consolidate RRI practices in an era where the speed of technology implementation is key but responsibility for its adoption is mandatory (Tait et al., 2017; Shelley-Egan et al., 2020).

The surveys we analyzed varied widely in methodology, and more standardized approaches across countries and over time would be important for such future studies. Good examples to follow are Eurobarometer surveys and international surveys that demand a higher collaboration between teams and offer a consistent overview that may transform a cross-sectional view into a more longitudinal one, allowing for more robust hypothesized theories over time (Stockemer, 2019a; Stockemer, 2019b). Co-authorship analysis for the studies included in the present review (Supplementary Figure S2) enabled addressing the connectedness of the authors involved. Although some extensive networks can be seen, most studies seem authored by independent groups of researchers. More collaborations may benefit methodological consistency in future studies.

Additionally, the bioethics literature on biotechnology recognizes a wider range of issues than those that have been covered in the public attitude surveys, such as eugenics, access to technology, funding of genome technologies, and social justice. These are subjects that impact the public and which they often care about, and should be included in future studies as well (Isasi et al., 2016; Nuffield Council of Bioethics, 2016; Brokowski and Adli, 2019). In policy-making, principles such as solidarity, social justice, and the welfare of future generations are worth considering in the case of GE (Halpern et al., 2019; Mulvihill et al., 2017). Finally, it is important to include an assessment of technology awareness and knowledge as part of the survey. Many surveys indicate low levels of knowledge and awareness, and these factors seem to be related to opinion, at least to some extent.

## Data Availability

The original contributions presented in the study are included in the article/Supplementary Material; further inquiries can be directed to the corresponding authors.
