# Supplementary material for: What do people think about genetic engineering? A systematic review of questionnaire surveys before and after the introduction of CRISPR
Source: Front Genome Ed. 2023 Dec 19;5:1284547. doi: 10.3389/fgeed.2023.1284547 (PMC10773783; doi:10.3389/fgeed.2023.1284547)
Supplement: Supplementary file 1 [file Table1.DOCX]

**Pre-CRISPR (1987-2012)**

|  | | **Approvers (in a total of 10 respondents)** | | | | | | |
| --- | --- | --- | --- | --- | --- | --- | --- | --- |
|  |  | **Genetic modification of animals** | | | **Genetic modification of humans** | | | |
| **Authors (Year)** | **Country(ies)** | **Transplants and/or medicines** | **Meat (Pork, Sheep, Cow)** | **Milk (Cow, Sheep)** | **Somatic (disease)** | **Somatic (enhancement)** | **Germline (disease)** | **Germline (enhancement)** |
| Office of Technology Assessment (OTA) (1987) | US |  | 7 (farm animals) |  | 8  ***Prevent – 8  9 - children | 5 | ***Prevent – 8  8 (non-fatal) | 4 (intelligence), 4 (physical) |
| Macer DRJ (1992) | Japan | - | - | - | 5  7 - children | - | - | - |
| Macer DRJ et al (1995) | Asia/Oceania |  |  |  | 10 (TH), 9 (NZ, AU and IS), 8 (J and RU), 7 (IN)  ***Prevent  8 (NZ, AU, IN, TH, RU and IS), 7 (J)  Children  8 (AU, TH, IN, NZ and IS), 7 (J), 6 (RU) | More ethical  8 (TH), 6 (IN), 3 (NZ, AU, RU and IS), 2 (J) | 10 (TH), 9 (AU, IN), 8 (NZ, J, RU and IS)  Non-fatal  9 (TH), 8 (AU, NZ), 7 (RU and IS), 6 (J, IN) | Physical  8 (TH), 6 (IN)  4 (RU), 3 (AU and J), 2 (NZ and IS)  Intelligence  7 (TH and IN), 3 (AU, J and RU), 2 (NZ and IS) |
| Eurobarometer 35.1 (1991) | EC12 | 9 | 4 | 4 | 7 | - | - | - |
| Eurobarometer 39.1 (1993) | EC12 | - | 4 | 4 | 7 | - | - | - |
|  | | **Approvers (in a total of 10 respondents)** | | | | | | |
|  |  | **Genetic modification of animals** | | | **Genetic modification of humans** | | | |
| **Authors (Year)** | **Country(ies)** | **Transplants and/or medicines** | **Meat (Pork, Sheep, Cow)** | **Milk (Cow, Sheep)** | **Somatic (disease)** | **Somatic (enhancement)** | **Germline (disease)** | **Germline (enhancement)** |
| Eurobarometer 46.1 (1996) | EU15 | 4 - mice and pigs | - | - | - | - | - | - |
| Eurobarometer 58.0 (2002) | EU15 | 4 | - | - | - | - | - | - |
| Eurobarometer 63.1 (2005) | EU23 | - | 3 | - | 5 | - | - | - |
| Sato et al (2006) | Japan | - | - | - | Opinion formation increased greatly after the first gene therapy success (only 56% formed an opinion) | | | |
| Barnett et al (2007) | UK | - | - | - | Trust in government and people in charge reveals favoring to allow gene therapy  Belief in public involvement, awareness, interest and levels of education don’t favor to allow for gene therapy | | | |
| Eurobarometer 73.1 (2010) | EU27 | 6 | - | - | 6 | 5 | 4 | - |
| Ng MAC et al (2000) | Japan | 5 (mice)  3 (pigs) | 5 | 4 | 7  ***Prevent - 6 | - | 7 | 5  3 (physical)  2 (intelligence) |
| Macer DRJ et al (2000) | Asia/Oceania | - | 8 (TH), 7 (IN), 6 (AU and J), 5 (NZ), 4 (IS), 3 (RU) | 8 (TH), 7 (IN), 4 (NZ, AU, J and IS), 2 (RU) | 10 (TH), 9 (NZ, AU and IS), 8 (J and RU), 7 (IN)  ***Prevent  8 (NZ, AU, IN, TH, RU and IS), 7 (J) | More ethical  8 (TH), 6 (IN), 3 (NZ, AU, RU and IS), 2 (J) | 10 (TH), 9 (AU and IN), 8 (NZ, J, RU and IS)  Non-fatal  9 (TH), 8 (AU, NZ), 7 (RU and IS), 6 (J, IN) | Physical  8 (TH), 6 (IN), 4 (RU), 3 (AU and J), 2 (NZ, IS)  Intelligence  7 (TH and IN), 3 (RU, AU and J), 2 (NZ and IS) |
|  | | **Approvers (in a total of 10 respondents)** | | | | | | |
|  |  | **Genetic modification of animals** | | | **Genetic modification of humans** | | | |
| **Authors (Year)** | **Country(ies)** | **Transplants and/or medicines** | **Meat (Pork, Sheep, Cow)** | **Milk (Cow, Sheep)** | **Somatic (disease)** | **Somatic (enhancement)** | **Germline (disease)** | **Germline (enhancement)** |
| Evans MDR et al (2005) | Australia | - | - | - | - | - | 4 (Serious defect)  3 (Minor defect)  2 (Aggression and violence) | 1 - cosmetic |
| Cook AJ et al (2004) | New Zealand | 5 | - | - | 4 | - | - | - |
| Human Genetics Commission (2001) | United Kingdom | - | - | - | 9  8 - children | - | - | - |
| Sturgis P et al (2005) | United Kingdom | - | - | - | 9 – cystic fibrosis, 8 – heart disease, 6 – Baldness  7 – schizophrenia  6 – less aggressive  *** Prevent  7 – heart disease  2 - baldness | 4 – average height  2 – height, intelligence, sexual option | 6-8 – cystic fibrosis, 5-6 – heart disease  2-4 - baldness | 1 – sex of unborn baby |
| Marteau T et al (1995) | United Kingdom | - | - | - | 2 - aggressive behavior and alcoholism | 1 – adults (intelligence/specific skills)  1 – children (appearance/behavior) | - | - |
| Hampel J et al (2000) | Germany | 4 – lab: medical | 2 – farm: agricultural | - | 7 | - | - | - |
|  | | **Approvers (in a total of 10 respondents)** | | | | | | |
|  |  | **Genetic modification of animals** | | | **Genetic modification of humans** | | | |
| **Authors (Year)** | **Country(ies)** | **Transplants and/or medicines** | **Meat (Pork, Sheep, Cow)** | **Milk (Cow, Sheep)** | **Somatic (disease)** | **Somatic (enhancement)** | **Germline (disease)** | **Germline (enhancement)** |
| Norton J et al (1998) | Australia | - | 3 - sheep and pork | - | - | - | - | - |
| Magnusson MK and Hursti UKK (2002) | Sweden | - | 2 - pork and salmon | - | - | - | - | - |
| Macer DRJ (1997) | Asia/Oceania | - | 8 (TH), 7 (J and IN), 5 (AU and NZ), 4 (IS), 3 (RU) | 8 (TH and IN), 4 (J, AU, NZ and IS), 2 (RU) | - | - | - | - |
| Macer DRJ and Ng MAC (2000) | Japan | 3 | 5 | 4 | - | - | - | - |
| Inaba M and Macer DRJ (2003) | Japan | 5 - mosquitoes | 5 | 4 | - | - | - | - |
| Small BH, Parminter TG and Fisher MW (2005) | New Zealand | 2 | - | 2 | - | - | - | - |
| Nayga RM (2006) | US and South Korea | - | 3 (US)  2 (South Korea) | - | - | - | - | - |
| Govindasamy R et al (2008) | South Korea | - | 2 | - | - | - | - | - |
| Hallman WK et al (2002) | US | 8 - sheep | 3 | 8 - sheep | - | - | - | - |
|  |  |  |  |  |  |  |  |  |
|  | | **Approvers (in a total of 10 respondents)** | | | | | | |
|  |  | **Genetic modification of animals** | | | **Genetic modification of humans** | | | |
| **Authors (Year)** | **Country(ies)** | **Transplants and/or medicines** | **Meat (Pork, Sheep, Cow)** | **Milk (Cow, Sheep)** | **Somatic (disease)** | **Somatic (enhancement)** | **Germline (disease)** | **Germline (enhancement)** |
| Hallman WK et al (2003) | US | - | 3 | - | - | - | - | - |
| Puduri V et al (2004) | US | - | 3 | - | - | - | - | - |
| Macer DRJ et al (1997) | Japan and New Zealand | Pigs  5 (J)  3 (NZ)  Mice  6 (J)  5 (NZ) | - | 4 (J and NZ) | - | - | - | - |
| Inaba M and Macer DRJ (2003) | Japan | 3 | - | - | - | - | - | - |

**CRISPR period (2013-2022)**

|  | | **Approvers (in a total of 10 respondents)** | | | | | | |
| --- | --- | --- | --- | --- | --- | --- | --- | --- |
|  |  | **Genetic modification of animals** | | | **Genetic modification of humans** | | | |
| **Authors (Year)** | **Country(ies)** | **Transplant/Medicines** | **Meat/Milk** | **Welfare** | **Somatic (disease)** | **Somatic (enhancement)** | **Germline (disease)** | **Germline (enhancement)** |
| Chikhazhe TL (2015) | New Zealand | 1 | 1 | 6 | 2 | 1 | - | - |
| McCaughey et al (2016) | Global |  |  |  | 6 (life-threatening and debilitating) | - | 6 (life-threatening and debilitating) | 3 |
| STAT and Harvard (2016) | US |  |  |  | - | - | 3 | 1 (Intelligence of physical) |
| Funk, Kennedy, Sciupac (2016) | US |  |  |  | - | - | 5  ***Prevent: 1-4 | - |
| Cormick C and Mercer R (2017) | Australia | 5 | 3 | - | 7 (General) | | | |
| Chen C and Liang Z (2017) | China |  |  |  | 6  Disease: 7-8  Non-disease (high cholesterol): 3 | Intelligence: 2  Skin color: 1 | 6 | - |
| Gaskell et al (2017) | Europe |  |  |  | 8 | 2 | 6 | 0 |
|  |  |  |  |  |  |  |  |  |
| Scheufele et al (2017) | US | - | - | - | 6 | 4 | 6 | 3 |
| Weisberg et al (2017) | US | - | - | - | Risk -7 ; No risk - 8 | | | |
| Wang J-H et al (2017) | China | - | - | - | 8 – adults and children | 4 | 6 | 4 |
| Hopkins and van Mill (2017) | UK | 7 (mosquitoes and organs) | 5 – efficiency of food  3 – profit | 7 – resistant to disease  6 – invasive species  5 – control pest and hornless cows | 8 - (in)curable  7 – non-life threatening  6 - Disorder not inherited | 5 – prolong life  2 – cosmetic  3 - intelligence | 8 | - |
|  | | **Approvers (in a total of 10 respondents)** | | | | | | |
|  |  | **Genetic modification of animals** | | | **Genetic modification of humans** | | | |
| **Authors (Year)** | **Country(ies)** | **Transplant/Medicines** | **Meat/Milk** | **Welfare** | **Somatic (disease)** | **Somatic (enhancement)** | **Germline (disease)** | **Germline (enhancement)** |
| Hendriks S et al (2018) | The Netherlands | - | - | - | 9 | - | 7 – Neuromuscular  3 - HIV | 2 |
| Uchiyama et al (2018) | Japan | - | - | - | The highest the awareness, the highest the support | | | |
| Lakomý M et al (2018) | Europe | - | 3-6 | - | 8-9 (disease)  ***Prevent  8-9 (Disease)  7-9 (Disabilities) | - | - | 3-5 |
| Pew Research Center (2018) | US |  |  |  | - | - | 7 (Treat serious)  ***Prevent - 6 | 2 (Intelligence) |
| Funk C and Heferon M (2018) | US | 6 (transplants)  7 (mosquitoes) | 4 | - |  |  |  |  |
| McCaughey T et al (2019) | Global |  |  |  | 6 (life-threatening and debilitating) | - | 6 (life-threatening and debilitating) | 3 |
| Critchley C et al (2019) | Australia | 7 | 6 | - | ***Prevent - 8 | 5 | ***Prevent - 8 | 4 |
| McConnachie E et al (2019) | US | - | 6 | 9 |  |  |  |  |
| Yunes MC et al (2019) | Brazil | - | 4 | 3 |  |  |  |  |
| Kohl PA et al (2019) | US |  |  | Wildlife - 1 |  |  |  |  |
