# Supplementary material for: What do people think about genetic engineering? A systematic review of questionnaire surveys before and after the introduction of CRISPR
Source: Front Genome Ed. 2023 Dec 19;5:1284547. doi: 10.3389/fgeed.2023.1284547 (PMC10773783; doi:10.3389/fgeed.2023.1284547)
Supplement: Supplementary file 2 [file Table15.docx]

**Supplementary files captions**

Fig S1. Public support for genetic modification in different animals worldwide for a proportion of 10 citizens upon survey inquiry in pre-CRISPR and CRISPR periods.

Fig S2. Coauthorship map regarding the 53 primary publications of the present study. Analysis was done with Bibexcel (homepage.univie.ac.at/juan.gorraiz/bibexcel/). Network visualization was done in VOSviewer (vosviewer.com).

Table S1. Questionnaire characteristics from surveys applied in pre-CRISPR and CRISPR periods

Table S2. Critical appraisal of questionnaires from surveys applied worldwide in pre-CRISPR and CRISPR periods

Table S3. Results from surveys about genetic modification of humans applied to the general public during pre-CRISPR period

Table S4. Results from surveys about genetic modification of animals applied to the general public during pre-CRISPR period

Table S5. Results from surveys about genome editing of humans applied to the general public during CRISPR period
