# Supplementary figures and images for: What do people think about genetic engineering? A systematic review of questionnaire surveys before and after the introduction of CRISPR
Source: Front Genome Ed. 2023 Dec 19;5:1284547. doi: 10.3389/fgeed.2023.1284547 (PMC10773783; doi:10.3389/fgeed.2023.1284547)

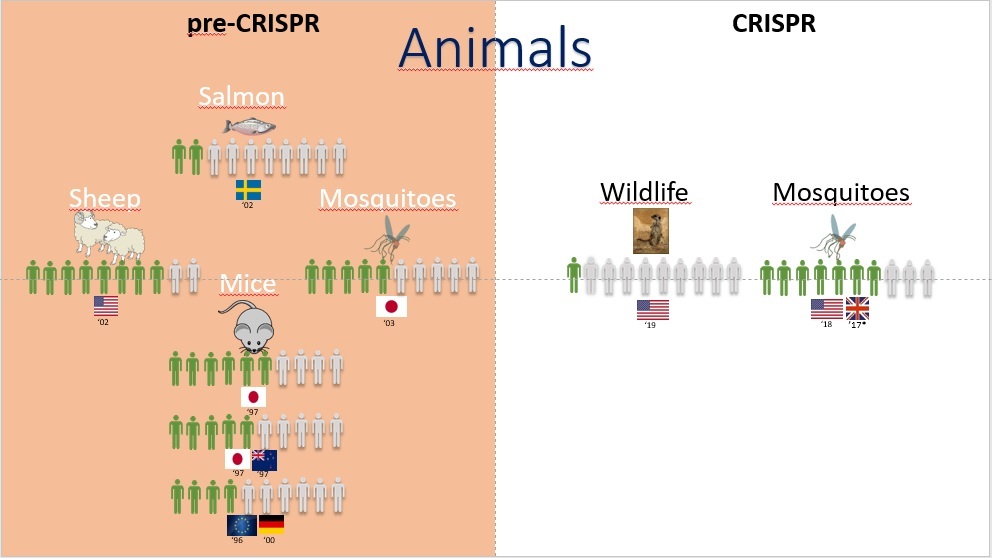

Supplement: Supplementary file 3 [file Image1.JPEG]

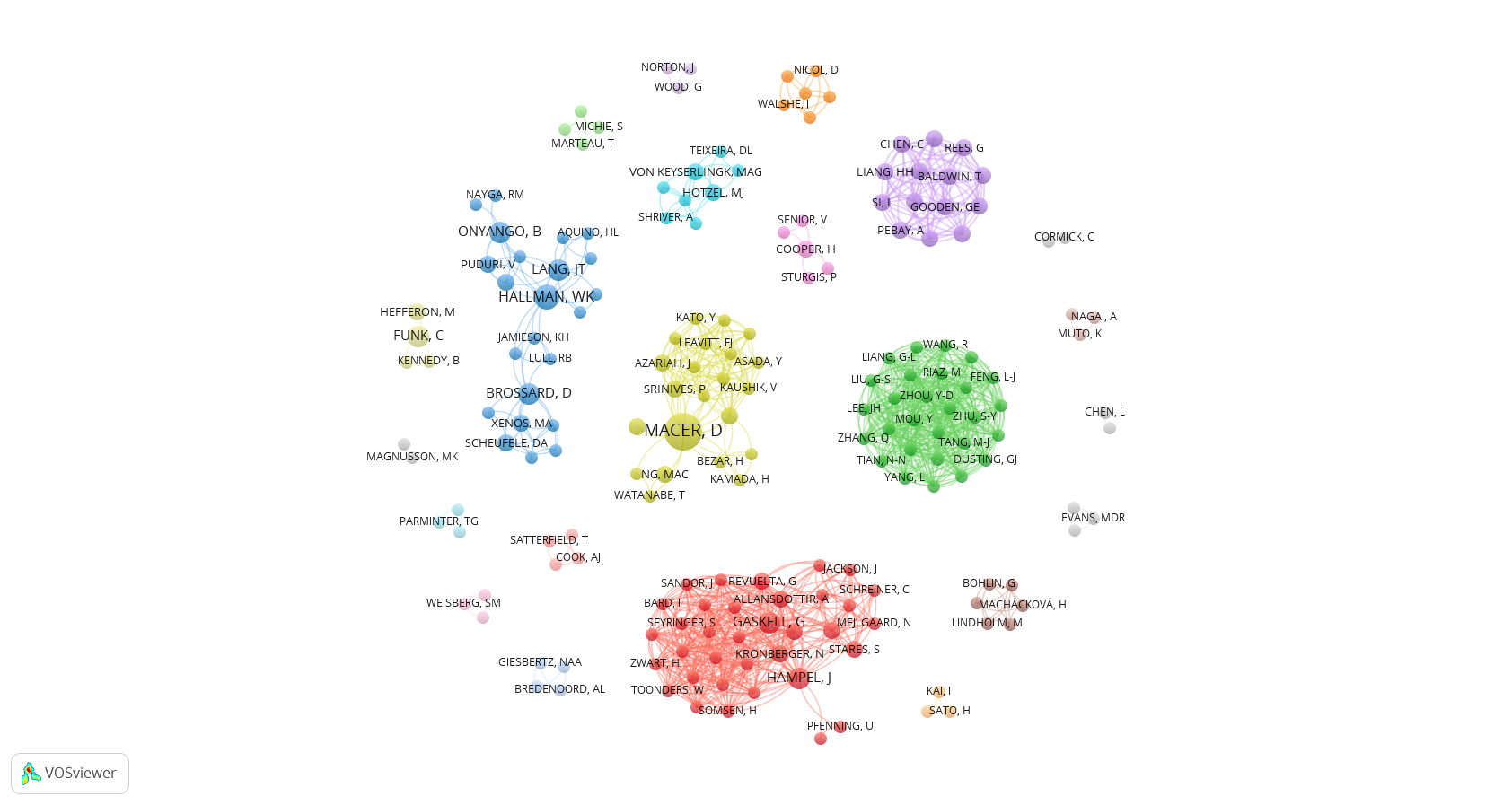

Supplement: Supplementary file 7 [file Image2.PNG]
