# Supplementary material for: What do people think about genetic engineering? A systematic review of questionnaire surveys before and after the introduction of CRISPR
Source: Front Genome Ed. 2023 Dec 19;5:1284547. doi: 10.3389/fgeed.2023.1284547 (PMC10773783; doi:10.3389/fgeed.2023.1284547)
Supplement: Supplementary file 4 [file Table2.DOCX]

| **Authors (year)** | **Type of publication** | **Recruitment** | **Country (Language)** | **Representation of sample** | **Sample size** | **Humans/Animals** | **GM Animals** | **Gene therapy** | **Terms/Language used for Themes** | **Topics covered in the questionnaires** | | |
| --- | --- | --- | --- | --- | --- | --- | --- | --- | --- | --- | --- | --- |
|  |  |  |  |  |  |  |  |  |  | **Awareness** | **Knowledge** | **Attitude/Acceptance** |
| OTA (1987)  [34] | Survey report | Telephone (RDD) | US (English) | Rep (>=18 y)  Oct-Nov 1986 | 1273 | Humans, human cells  Farm animals and laboratory animal cells | Food  Medical | Germline  Somatic | Genetic engineering and gene therapy, enhancement, eugenics | Yes | Yes | Yes |
| Comission of the European Communities Directorate-General Science, Research and Development  (Eurobarometer 35.1) (1991)  [37] | Survey report | Face-to-face Interview | B, DK, D-West, D-East, GR, E, FR, IRL, IT, L, NI, NL, PT, UK (EC12 own languages) | Rep (>=15 y)  28 Mar – 25 Apr 1991 | 12800 | Humans, human cells  Farm Animals | Food | Somatic | Biotechnology and Genetic Engineering | Yes | Yes (Objective and Subjective) | Yes |
| Macer, (1992)  [35] | Journal Article | Hand distribution and mail response | Japan (Japanese) | Rep  Aug-Oct 1991 | 538 | Humans | - | Somatic | Genetic engineering or manipulation | Yes | Yes | Yes |
| Comission of the European Community Directorate-General Science, Research and Development Unit XII/E/1  (Eurobarometer 39.1)  (1993)  [38] | Survey report | Face-to-face Interview | B, DK, D-West, D-East, D-All, GR, E, FR, IRL, IT, L, NL, PT, UK (EC12 own languages) | Rep  Conducted in 1993 | 13032 | Humans, human cells  Farm Animals | Food | Somatic | Biotechnology and Genetic Engineering | Yes | Yes (Objective and Subjective) | Yes |
| Macer DRJ, Akiyama S, Alora AT, Asada Y, Azariah J, Azariah H, et al, (1995)  [36] | Journal Article | Letter (Mail) | NZ, AU, IN, IS (English),  RU (Russian), TH (Thai),  J (Japanese) | Rep  Conducted in 1993 | NZ (329)  AU (201)  J (352)  IN (568)  IS (50)  RU (446)  TH (680) | Humans | - | Germline  Somatic | Gene therapy  And Genetic Engineering, enhancement, eugenics | Yes | No (Indirect upon open questions) | Yes |
| Marteau T, Michie S, Drake H, Bobrow M, (1995)  [50] | Journal Article | Face-to-face interviews | UK (English) | Rep (18-45 y)  July 1994 | 975 | Humans | - | Somatic | “Implanting genes” (gene manipulation) | No | No | Yes |
| Ng MAC, Takeda C, Watanabe T, Macer D (2000)  [43] | Journal Article | Anonymous cover envelope (Mail) | Japan (Japanese) | Rep  November 1999-January 2000 | 297 | Humans and Animals: Laboratory mice and Pigs with human hearts | Food  Medical | Germline  Somatic | Gene therapy;  Genetic modification (GM); Biotechnology | Yes | No | Yes |
| Macer DRJ, Azariah J, Srinives P (2000)  [44] | Online Article | Letter (Mail) | NZ, AU, IN, IS (English), RU (Russian), TH (Thai),  J (Japanese) | Rep | NZ (329)  AU (201)  J (352)  IN (568)  IS (50)  RU (446)  TH (680) | Humans and animals | Food | Germline  Somatic | Genetic engineering, GMOs;  Gene therapy;  Biotechnology | No | No | Yes |
| UK Human Genetics Comission (2001)  [48] | Survey report | Interview | UK (English) | Rep (>=16 y)  6 Oct – 17 Dec 2000 | 1038 | Humans | - | Somatic | Genetics, human genetics and new genetic developments | Yes | Yes (Identification of diseases) | Yes |
| Cook AJ, Fairweather JR, Satterfield T, Hunt LM (2004)  [46] | Research Report | Mail | NZ  (English) | Rep (>=15 y)  Dec, 2003 – Jan, 2004 | 701 | Humans, Animals | Medical | Somatic | Biotechnology, Genetic modification, genetic engineering, genetically modified organisms | Yes | No | Yes |
| Evans MDR, Kelley J, Zanjani ED (2005)  [45] | Journal Article | Personally-addressed mail, post return | AU (English) | Rep  2002 annual survey | 1403 | Humans | - | Germline | Gene therapy, enhancement, eugenics | No | Yes (Indirect and predictive) | Yes |
| Sturgis P, Cooper H, Fife-Schaw C (2005)  [49] | Journal Article | Interview (Method not specified) | UK (English) | Rep  (BSAS and WT: >=18y)  2000 (BSAS)  1999 (WT) | Wellcome Trust (696)  British Social Attitudes Survey (3426) | Humans | - | Germline  Somatic | Gene therapy | No | Yes (Objective) | Yes |
| European Comission Directorate-General for Research.    (Eurobarometer 64.3)  (2005)  [41] | Survey Report | Face-to-face interview | EU25  (EU25 own languages) | Rep  (>=15 y)  November 05 to December 07, 2005 | 25000 | Humans | - | Somatic (but undefined) | Gene therapy | Yes | Yes | Yes |
| Sato H, Akabayashi A, Kai I (2006)  [87] | Journal Article | Mail | Japan (Japanese) | Rep  Jan-Feb, 2003 | 900 | Humans | - | Somatic (but undefined) | Gene therapy | Yes | No | Yes (Evolution on period and not trends) |
| Barnett J, Cooper H, Senior V (2007)  [85] | Journal Article | Interview | UK (English) | Rep (>=18 y) | 3272 | Humans | - | Somatic | Gene therapy | Yes | Yes | Yes |
| European Comission Directorate-General for Research  (Eurobarometer 73.1) (2010)  [42] | Survey Report | Face-to-face interview | EU27 (EU25 + Iceland and Croatia (EU27 own languages) | Rep (>=15 y)  Jan-Feb 2010 | 26676 | Humans/Animals | Medical | Somatic | Biotechnology, Gene therapy,  Human genes into animals | No | No | Yes |
| European Comission Directorate-General Science, Research and Development XII  (Eurobarometer 46.1) (1996)  [39] | Survey report | Face-to-face interview | EC12+ FIN, S, AT (EU15 own languages) | Rep  October 18 to November 22, 1996 | 16246 | Humans/Animals | Medical | - | Genetic engineering,  Biotechnology and modern biotechnology | Yes | Yes (Objective) | Yes |
| Macer DRJ (1997)  [54] | Book chapter | Letter (Mail) | NZ, AU, IN, IS (English),  RU (Russian), TH (Thai),  J (Japanese) | Rep  Conducted in 1993 | NZ (329)  AU (201)  J (352)  IN (568)  IS (50)  RU (446)  TH (680) | Animals | Food  Medical | - | Genetic engineering  Genetically modified organisms (GMO)  Biotechnology | Yes | No  (Indirect upon open questions) | Yes |
| Macer D, Bezar H, Harman N, Kamada H, Macer N (1997)  [63] | Online Article | Telephone | Japan (Japanese), NZ (English) | Rep  28 December 1996 to 8 April 1997 (Japan)  February 1997 to 18 June 1997 (NZ) | Japan (405)  NZ (489) | Animals | Medical | - | Genetic engineering | Yes | Yes (Objective) | Yes |
| Norton J, Lawrence G, Wood G (1998)  [52] | Journal Article | Mail | Australia (English) | Rep  Dec 1996 – Jan 1997 | 969 | Humans  Animals | Food  Welfare | - | Genetic engineering,  Genetically-engineered organisms | No | No | Yes |
| Hampel J, Pfenning U, Peters HP (2000)  [51] | Journal Article | Telephone | Germany (German | Rep (>= 16 y)  17 April-18 May 1997 | 1501 | Humans Animals | Food  Medical | Germline | Genetic engineering  Genetic therapy | Yes (Communication on the topic) | Yes (Subjective) | Yes |
| Macer D, Ng MAC (2000)  [55] | Journal Article (Feature) | Mail | Japan (Japanese) | Rep  Nov 1999 – Feb 2000 | 297 | Animals:  Healthier meat and Cows which produce more milk | Food | - | Genetic engineering;  Biotechnology;  GMOs | No | No | Yes |
| Magnusson MK, Hursti UKK  (2002)  [53] | Journal Article | Mail  + Mail and Telephone for initial non-respondents = | Sweden (Swedish) | Rep  (18-65 y)  May-June 2000 | 786 | Animals: pork and salmon | Food | - | Genetic engineering | No | Yes | Yes |
| Hallman WK, Adelaja AO, Schilling BJ, Lang JT (2002)  [60] | Survey Report | Computer-assisted telephone interviews (CATI) | US (English) | Rep (>=18 y)  March 15 – Apr 4 2001 | 1203 | Animals  (Hybrid) | Food  Medical | - | Genetic modification;  Genetic engineering;  Biotechnology | Yes | Yes (Self-rated and Objective) | Yes |
| European Comission Directorate General for Research  Eurobarometer 58.0 (2002)  [40] | Survey Report | Face-to-face interviews | EU15 (EU own languages) | Rep (>=15 y)  September 1 to October 7, 2002 | 16500 | Animals | Medical | - | Biotechnology  Genetics | Yes | Yes (Objective and subjective) | Yes |
| Inaba M, Macer DRJ (2003)  [56] | Online Article | Personal delivery envelopes + Telephone surveys (2000) and mail (1997, 1993, 1991) | Japan (Japanese) | Rep | 378 | Animals/Humans | Food  Medical | Germline  Somatic | Biotechnology,  Genetic engineering;  Genetic modification (GM);  Gene therapy | Yes | No | Yes |
| Inaba M, Macer DRJ (2003b)  [64] | Online Article | Mail (2000) Personal delivery (2003) | Japan | Rep | 2000 – 297  2003 - 378 | Animals | Food  Medical | - | Biotechnology  Genetic engineering  Genetic modification | Yes | No | Yes |
| Hallman WK, Hebden WC, Aquino HL, Cuite CL, Lang, JT (2003)  [61] | Survey Report | Computer-assisted telephone interviews (CATI) | US (English) | Rep (>= 18y)  27 Feb – 1 Apr 2003 | 1201 | Animals | Food | - | Genetic modification (GM); Genetic engineering;  Biotechnology | Yes | Yes (Self-rated and objective) | Yes |
| Puduri V, Govindasamy R, Lang JT, Onyango B (2004)  [62] | Journal Article | Telephone | US (English) | Rep  Non-institutionalized adults  Conducted in 2004 | 1201 | Animals | Food | - | Genetic modification; Biotechnology | No | Yes (Objective) | Yes |
| Small BH, Parminter TG, Fisher MW (2005)  [57] | Journal Article | Post | NZ (English) | Rep (>= 18y)  March-June 2001 | 1684 | Animals: Cattle | Food  Medical | - | Genetic modification;  Genetic engineering; | No | No | Yes |
| Nayga RM, Fishera MG, Onyango B (2006)  [58] | Journal Article | CATI (US)  Face-to-face interviews (South Korea) | US (English) and South Korea (South Korean) | Rep  US (>= 18y)  South Korea (20-59 y)  US: 27 Feb – 1 Apr 2003  South Korea: 10 Apr – 9 May 2003 | 1201 (US)  903 (South Korea) | Animals | Food | - | Genetic modification; Biotechnology | Yes | Yes | Yes |
| Govindasamy R, Onyango B, Hallman WK, Jang H-M, Puduri V (2008)  [59] | Journal Article | Face-to-face interviews (South Korea) | South Korea (South Korean) | Rep  US (>= 18y)  South Korea (20-59 y) | 903 | Animals | Food | - | Genetic modification; Biotechnology | No | Yes | Yes |
| Chikhazhe TL (2015)  [67] | Thesis | Online (Small City) | NZ (English) | Rep  2013 census | 353 | Animals/Humans | Food  Medical | Somatic | Genetic modification | Yes | No | Yes |
| McCaughey T, Sanfilippo PG, Gooden GEC, Budden DM, Fan L, Fenwick E, et al (2016)  [72] | Journal Article | Online (Via social media) | 185 countries (English) | Voluntary  (11-90 y).  June 2015 | 12562 (10067 complete responses) | Humans | - | Germline  Somatic | Gene editing  Genetic manipulation | No | No | Yes |
| STAT and Harvard T.H. Chan School of Public Health (2016)  [74] | Survey Report | Telephone  (cell phone and landline) | US (English) | Rep (>=18 y)  Jan 13-17, 2016 | 1000 | Humans | - | GermlineSomatic | Changing the genes | Yes | No | Yes |
| Funk C, Kennedy B, Sciupac E  Pew Research Center (2016)  [76] | Survey Report | Webmail and Mail | US (English) | Rep (>=18 y)  Mar 2-28, 2016 | 4726 | Humans | - | Germline | Gene editing  Human enhancement | Yes | No | Yes |
| Cormick C, Mercer R (2017)  [71] | Survey report | Online + booster CATI (phone) | Australia (English) | Rep | 1255 | Animals | Food  Medical | - | Gene editing  Genetic modification  Genetic engineering  Biotechnology | Yes | No | Yes |
| Chen, C, Liang, Z (2017)  [82] | Survey Report | Not mentioned (Poll) | China (Chinese) | Rep | 4196 | Humans | - | Somatic | Gene editing | No | No | Yes |
| Gaskell G, Bard I, Allansdottir A, da Cunha RV, Eduard P, Hampel J, et al (2017)  [78] | Journal Article (Correspondence) | Online | Austria, Denmark, Germany, Hungary, Iceland, Italy, the Netherlands, Portugal, Spain, UK (EEA-10 countries) and the United States (Countries own language) | Rep | 11,716 | Humans | - | Germline  Somatic | Gene therapy  Gene enhancement | No | No | Yes |
| Scheufele DA, Xenos MA, Howell EL, Rose KM, Brossard D, Hardy BW (2017)  [75] | Journal Article | YouGov platform (online) | US (adults) - English  YouGov | Rep  (Dec 2016-Jan 2017) | 1600 | Humans | - | Germline  Somatic | Gene editing  Somatic  therapy  Germline  therapy  Somatic  enhancement  Germline  enhancement | No | Yes  (Factual knowledge) | Yes |
| Weisberg SM, Badgio D, Chatterjee A (2017)    [83] | Journal Article | Online | US (English) | Rep - Quota | 1249 (Study 1)  1244 (Study 2) | Humans | - | Somatic | Genetic modification  Genetic editing  Genetic engineering,  Genetic surgery  Genetic hacking | No | No | Yes |
| Wang J-H, Wang R, Lee JH, Iao TWU, Hu X, Wang Y-M, et al (2017)  [81] | Journal Article (Commentary) | Online (Social media announced) | China (Chinese) | Voluntary (11-90 y). (18-50)    24 Aug – 2 Nov, 2016 | 13201 | Humans | - | Germline Somatic | Gene therapy  Genetic enhancement | Yes | No | Yes |
| Hendriks S, Giesbertz NAA, Bredenoord AL, Repping S (2018)  [79] | Journal Article | Online | The Netherlands (Dutch) | Not Rep (11-90 y) – social media dissemination  (30 March for 4 weeks, 2016) | 1013 | Humans | - | Germline  Somatic | Genetic modification | No | No | Yes |
| Uchiyama M, Nagai A, Muto K (2018)  [86] | Journal Article | Online | Japan (Japanese) | Rep (20-69y)  Feb – March 2017 | 10881 | Humans | - | Somatic | Genome Editing | Yes | Yes (1 T or F question) | Yes |
| Lakomý M, Bohlin G, Hlavová R, Macháčková H, Bergman M, Lindholm M  ORION (2018)  [80] | Survey Report | Telephone (CATI) – mobile and landline | Czechia, Germany, Italy, Spain, Sweden, and the UK (Countries own languages) | Rep (16-79y)  25 Jan – 28 Feb, 2018 | 5870 | Humans | - | Germline  Somatic | Genome Editing | Yes | No | Yes |
| Funk C and Heferon M  Pew Research Center (2018)  [77] | Survey Report | Telephone (Landline and cell phone)  Participation via monthly self-administered web surveys | US (English) | Rep (>=18y)  23 April- 6 May, 2018  10 May – 6 June, 2018 (Knowledge index) | 2537 | Humans | - | Germline | Gene editing  Changing genes | Yes | Yes  (Objective) | Yes |
| Funk C and Heferon M  Pew Research Center (2018b)  [65] | Survey Report | Telephone (Landline and cell phone) recruitment  Participation via monthly self-administered web surveys | US (English) | Rep (>=18y)  23 April- 6 May, 2018  10 May – 6 June, 2018 (Knowledge index) | 2537 | Animals | Food  Medical  Welfare | - | Genetic engineering | No | Yes (Objective) | Yes |
| McCaughey T, Budden DM, Sanfilippo PG, Gooden GEC, Fan L, Fenwick E, et al (2019)  [73] | Journal Article | Online | Global (English) | Voluntary (11-90 y). | 3935 free-text responses | Humans | - | Germline  Somatic | Genetic editing  Human Genetic engineering | Yes | No | Yes |
| Critchley C, Nicol D, Bruce G, Walshe J, Treleaven T, Tuch B (2019)  [70] | Journal Article | Telephone (CATI) and online | Australia (English) | Rep  2017 | 1004  501 (CATI)  503 (Online) | Humans and Animals | Food  Medical | Germline  Somatic | Genome editing | No | Yes (Subjective) | Yes |
| McConnachie E, Hotzel MJ, Robbins JA, Shriver A, Weary DM, von Keyserlingk MAG (2019)  [68] | Journal Article | Amazon Mechanical Turk (Online database) | US (English) | Rep | 434 | Animals: Cattle | Food  Welfare | - | Technology and agriculture (Pre-survey)  Genetic engineering (At survey);  “genetic modification”, and “genetically  modifying cows to be hornless” | Yes | Yes (Objective) | Yes |
| Yunes MC, Teixeira DL, von Keyserlingk MAG, Hotzel MJ (2019)  [69] | Journal Article | Face-to-face surveys | Brazil (Portuguese) | Voluntary (>=18 y)  Sep-Oct 2017 | 570 | Animals: Pigs | Food  Welfare | - | Animal production  Science and Technology  Gene editing  Biotechnology | Yes | Yes (Objective) | Yes |
| Kohl PA, Brossard D, Scheufele DA, Xenos MA (2019)  [84] | Journal Article | YouGov platform (online) – originally from Census Bureau 2010 | US (English) | Rep  Dec 2016 – Jan 2017 | 1600 | Wildlife | - | - | Gene editing | No | No | Yes |
| Lull RB, Akin H, Hallman WK, Brossard D, Jamieson KH (2019)  [66] | Journal Article | Telephone (Landline and cell phone) | US (English) | Rep  10 – 15 Nov 2016 | 1137 | Animals (Mosquitoes) | Food | - | Gene editing  Genetic engineering | No | Yes | Yes |
