# Supplementary material for: What do people think about genetic engineering? A systematic review of questionnaire surveys before and after the introduction of CRISPR
Source: Front Genome Ed. 2023 Dec 19;5:1284547. doi: 10.3389/fgeed.2023.1284547 (PMC10773783; doi:10.3389/fgeed.2023.1284547)
Supplement: Supplementary file 5 [file Table5.DOCX]

| **Authors (years)** | **Country** | **Topic** | **Scale** | **Questions** | **Answers** |
| --- | --- | --- | --- | --- | --- |
| European Comission Directorate-General Science, Research and Development XII  (Eurobarometer 46.1) (1996)  [39] | EC12 + FIN, S, AT | **Awareness in biotechnology**  **Objective knowledge** about biotechnology  **Attitudes** towards GM animals  - Laboratory research  - Human genes into animals for organ production | 4-point scale  • Tend to/Definitely Agree and Tend to/Definitely Disagree (4);  Dichotomous   - Likely/Unlikey + D/K; | Opinion on modern biotechnology applications:  For five different applications, two on animals  *-  Developing genetically modified animals for laboratory research (mice, cancer).*  *-  Introducing human genes into animals for transplants (pigs, heart)*  indicate agreement/disagreement that the application...?  *a) benefits society?*  *b) involves risks for society?*  *c) is morally acceptable?*  *d) should be encouraged?*    Exposure to information about modern biotechnology:  Over the last three months, have you heard anything about issues involving modem biotechnology?  *No*  *Yes, in newspapers*  *Yes, in magazines*  *Yes, on television*  *Yes, on the radio*  *Yes, but no longer remembers*    Knowledge of biotechnology:  10 factual statements – responding whether true or false  1. There are bacteria which live from waste water. (TRUE)  2. Ordinary tomatoes do not contain genes, while genetically modified tomatoes do. (FALSE - IMAGE)  3. The cloning of living things produces exactly identical offspring. (IMAGE)  4. By eating a genetically modified fruit, a person's genes could also become modified. (IMAGE)  5. Viruses can be contaminated by bacteria. (FALSE)  6. Yeast for brewing beer consists of living organisms. (TRUE)  7. It is possible to find out in the first few months of pregnancy whether a child will have Down's syndrome.  (TRUE)  8. Genetically modified animals are always bigger than ordinary ones. (FALSE - IMAGE)  9. More than half of the human genes are identical to those with chimpanzees. (TRUE)  10. It is impossible to transfer animal genes into plants. (FALSE - IMAGE)    Opinion on biotechnology versus traditional breeding methods:  Indicating agreement/disagreement with a series of statement including  *- Only traditional breeding methods should be used, rather than changing the hereditary characteristics of plants and animals through modem biotechnology.*  *- Traditional breeding methods can be as effective as modem biotechnology in changing the hereditary characteristics of plants and animals.* | \| *Application* \| *Beneficial* \| *Risky* \| *Acceptable* \| *Encouraged* \| \| --- \| --- \| --- \| --- \| --- \| \| Food \| 54 \| 61 \| 50 \| 44 \| \| Plants \| 69 \| 48 \| 62 \| 58 \| \| Medicines \| 80 \| 47 \| 40 \| 71 \| \| **Mice, cancer** \| **58** \| **54** \| **40** \| **43** \| \| **Pigs, hearts** \| **54** \| **61** \| **36** \| **39** \| \| Detection \| 83 \| 40 \| 74 \| 75 \|   - Consistently, GM mice for cancer research and introducing human genes in pigs to produce organs for human transplants feature as the least beneficial, riskier, less morally acceptable and that shouldn’t be encouraged at all (Similar to food production)   \| **Biotechnology** \| *EU 15 average* \| *D/K* \| \| --- \| --- \| --- \| \| Traditional breeding methods… \|  \|  \| \| *… should be the only way* \| **56** \| 15 \| \| *… can be as effective as modern biotechnology* \| **43** \| 27 \|   - More than half of the Europeans think that only traditional should be used rather than genetic modification of plants and animals.  - A lower portion although close followed think that both methods can be equally effective in the modification of hereditary characteristics from these organisms  - Almost half heard nothing at all about modern biotechnology and a similar proportion demonstrates objective knowledge in the matter  - Lower objective knowledge index is correlated with preference for traditional breeding methods  - Almost half of respondents didn’t hear about biotechnology it in the previous 3 months   \| **Familiarity with biotechnology** \| *EU 15 average* \| \| --- \| --- \| \| *No* \| 47 \| \| *Yes, in newspapers* \| 33 \| \| *Yes, in magazines* \| 21 \| \| *Yes, on television* \| 14 \| \| *Yes, on the radio* \| 8 \| \| *Yes, but no longer remembers* \| 7 \|  \| *Knowledge (Quiz)* \| *% correct answers* \| *D/K* \| \| --- \| --- \| --- \| \| *1* \| 83 \| 13 \| \| *2* \| 81 \| 12 \| \| *3* \| 68 \| 19 \| \| *4* \| 51 \| 35 \| \| *5* \| 48 \| 29 \| \| *6* \| 46 \| 35 \| \| *7* \| 36 \| 30 \| \| *8* \| 35 \| 35 \| \| *9* \| 27 \| 44 \| \| *10* \| 19 \| 33 \|   - Respondents reveal a very dispersed knowledge about biotechnology  - Average of 4.95/10 was seen for EU15 regarding objective knowledge |
| Macer DRJ (1997)  [54] | NZ, AU, J, IN, IS, RU, TH | **Awareness** on:  - Genetic engineering  -Biotechnology  - Human gene therapy  **Approval** of GM animals:  - genes from humans  - genes from animals  - For food production  - For environment | Dichotomous   - Unacceptable/Acceptable + D/K   • Yes/No + D/K   - Approve/Disapprove + D/K   4-point scale   - No, Few, Some, Lot - 1. Agree 2. Agree Strongly 3. Neither 4. Disagree 5. Disagree Strongly   3-point scale   - 1. Not heard of 2. Heard of 3. Could explain it to a friend | Exposure to information about modern biotechnology:   - Heard or read about: - *Biotechnology* - *Human gene therapy* - *Genetic engineering* - *GMOs for food and medicines*   Provided no risk to humans and remote risks to environment, approve/disapprove of environmental release of the genetically engineered:  *-Produce healthier meat*  *-Cows which produce more milk*    Concerns regarding consuming products from genetically modified organisms   - *Dairy products* - *Vegetables* - *Meat* - *Medicines*     Acceptability of different types of genetic engineering   - *Using genes from another animal to make chicken less fatty* - *Using human genes to make chicken less fatty*     Agreement that genetically  modified plants and animals will help agriculture become less dependent on chemical pesticides | \| **Biotechnology** \| \| \| \| \| \| \| \| \| \| --- \| --- \| --- \| --- \| --- \| --- \| --- \| --- \| --- \| \| *Awareness* \| *NZ* \| *A* \| *J* \| *J (1991)* \| *IN* \| *TH* \| *RU* \| *IS* \| \| Not heard \| 23 \| 19 \| 6 \| 3 \| 10 \| 2 \| 8 \| 18 \| \| Heard \| 62 \| 56 \| 65 \| 65 \| 53 \| 57 \| 62 \| 62 \| \| Explain \| 15 \| 25 \| 29 \| 32 \| 37 \| 41 \| 30 \| 20 \|  \| **GMOs for food and medicines** \| \| \| \| \| \| \| \| \| \| --- \| --- \| --- \| --- \| --- \| --- \| --- \| --- \| --- \| \| *Awareness* \| *NZ* \| *A* \| *J* \| *J (1991)* \| *IN* \| *TH* \| *RU* \| *IS* \| \| Yes \| 80 \| 78 \| 61 \| 75 \| 82 \| 56 \| 77 \| 65 \|  \| **Genetic engineering** \| \| \| \| \| \| \| \| \| \| --- \| --- \| --- \| --- \| --- \| --- \| --- \| --- \| --- \| \| *Awareness* \| *NZ* \| *A* \| *J* \| *J (1991)* \| *IN* \| *TH* \| *RU* \| *IS* \| \| Not heard \| 9 \| 9 \| 9 \| 6 \| 17 \| 13 \| 14 \| 8 \| \| Heard \| 62 \| 49 \| 74 \| 68 \| 46 \| 58 \| 60 \| 82 \| \| Explain \| 29 \| 42 \| 17 \| 26 \| 37 \| 29 \| 26 \| 10 \|   - The majority of publics surveyed have “heard” of biotechnology and genetic engineering and a large portion of these are also aware of GMOs use for food and medicines production  - Japan public are the more aware on both Biotechnology and Genetic engineering and Indians on GMOs   \| **Dairy** \| \| \| \| \| \| \| \| \| \| --- \| --- \| --- \| --- \| --- \| --- \| --- \| --- \| --- \| \| *Concerns* \| *NZ* \| *A* \| *J* \| *J (1991)* \| *IN* \| *TH* \| *RU* \| *IS* \| \| No \| 27 \| 31 \| 16 \| 48 \| 48 \| 48 \| 37 \| 41 \| \| Few \| 22 \| 21 \| 40 \| 52 \| 26 \| 28 \| 63 \| 22 \| \| **Some** \| **20** \| **23** \| **26** \| **-** \| **14** \| **18** \| **-** \| **25** \| \| **Lot** \| **31** \| **25** \| **18** \| **-** \| **12** \| **6** \| **-** \| **12** \|  \| **Meat** \| \| \| \| \| \| \| \| \| \| --- \| --- \| --- \| --- \| --- \| --- \| --- \| --- \| --- \| \| *Concerns* \| *NZ* \| *A* \| *J* \| *J (1991)* \| *IN* \| *TH* \| *RU* \| *IS* \| \| No \| 25 \| 29 \| 14 \| 45 \| 47 \| 43 \| 31 \| 37 \| \| Few \| 18 \| 18 \| 39 \| 55 \| 25 \| 29 \| 69 \| 18 \| \| **Some** \| **22** \| **23** \| **28** \| **-** \| **13** \| **19** \| **-** \| **18** \| \| **Lot** \| **35** \| **30** \| **19** \| **-** \| **15** \| **9** \| **-** \| **27** \|  \| **Medicines** \| \| \| \| \| \| \| \| \| \| --- \| --- \| --- \| --- \| --- \| --- \| --- \| --- \| --- \| \| *Concerns* \| *NZ* \| *A* \| *J* \| *J (1991)* \| *IN* \| *TH* \| *RU* \| *IS* \| \| No \| 38 \| 46 \| 22 \| 50 \| 47 \| 52 \| 54 \| 45 \| \| Few \| 23 \| 21 \| 39 \| 51 \| 28 \| 25 \| 46 \| 10 \| \| **Some** \| **18** \| **20** \| **20** \| **-** \| **13** \| **15** \| **-** \| **20** \| \| **Lot** \| **21** \| **13** \| **19** \| **-** \| **12** \| **8** \| **-** \| **25** \|   - GMOs to produce medicines have the lowest concern whereas meat shows the highest followed by dairy products  - Asian and Oceanic countries approved GM pigs to produce less fatty meat  - Oceanic countries didn’t agree with cows to produce more milk compared to Asian ones  - Russians and Israelis didn’t approve both in their majority  - Most of the public (except for Russians and Israelis) agreed that GM plants and animals would help agriculture become less dependent upon pesticides   \| **GM pigs to produce meat less fatty** \| \| \| \| \| \| \| \| \| --- \| --- \| --- \| --- \| --- \| --- \| --- \| --- \| \| *Approve* \| *NZ* \| *A* \| *J* \| *IN* \| *TH* \| *RU* \| *IS* \| \| Yes \| 49 \| 54 \| 69 \| 73 \| 83 \| 35 \| 40 \| \| D/K \| 16 \| 11 \| 11 \| 7 \| 7 \| 20 \| 16 \|  \| **GM cows to produce more milk** \| \| \| \| \| \| \| \| \| --- \| --- \| --- \| --- \| --- \| --- \| --- \| --- \| \| *Approve* \| *NZ* \| *A* \| *J* \| *IN* \| *TH* \| *RU* \| *IS* \| \| Yes \| 36 \| 39 \| 44 \| 75 \| 84 \| 23 \| 38 \| \| D/K \| 19 \| 19 \| 24 \| 6 \| 9 \| 39 \| 20 \|   - GM pigs to produce meat less fatty are greatly approved by Asian (Japan, India, Thailand) publics, average accepted by Oceanic ones (Australia, New Zealand) and mostly rejected by Russian and Israeli ones  - As for GM cows all levels of acceptance decrease except in India and Thailand publics   \| **GM plants and animals to be less dependent pesticides** \| \| \| \| \| \| \| \| \| \| --- \| --- \| --- \| --- \| --- \| --- \| --- \| --- \| --- \| \| *Country* \| *NZ* \| *A* \| *J* \| *J (1991)* \| *IN* \| *TH* \| *RU* \| *IS* \| \| *++ totally agree*  *-- totally disagree* \| \| ++ \| 16 \| 18 \| 11 \| 9 \| 19 \| 18 \| 10 \| 12 \| \| + \| 48 \| 47 \| 31 \| 39 \| 37 \| 51 \| 34 \| 35 \| \| = \| 22 \| 25 \| 45 \| 45 \| 5 \| 15 \| 37 \| 33 \| \| - \| 12 \| 9 \| 10 \| 6 \| 12 \| 13 \| 14 \| 16 \| \| -- \| 2 \| 1 \| 3 \| 1 \| 8 \| 3 \| 5 \| 4 \|   - In their majority, countries agree with GMOs for less dependency on pesticides. The exception are Japanese which keep neutral as they were in 1991.   \| **Cross-species gene transfer** \| \| \| \| \| \| \| \| \| \| --- \| --- \| --- \| --- \| --- \| --- \| --- \| --- \| --- \| \| *Animal-animal* \| *NZ* \| *A* \| *J* \| *IN* \| *TH* \| *RU* \| *IS* \| \| **Acceptable** \| **29** \| **40** \| **20** \| **40** \| **68** \| **32** \| **26** \| \| Unacceptable \| 46 \| 40 \| 41 \| 27 \| 10 \| 35 \| 46 \| \| D/K \| 25 \| 20 \| 39 \| 33 \| 22 \| 33 \| 28 \| \| *Human-animal* \| *NZ* \| *A* \| *J* \| *IN* \| *TH* \| *RU* \| *IS* \| \| **Acceptable** \| **10** \| **16** \| **6** \| **16** \| **29** \| **10** \| **14** \| \| Unacceptable \| 78 \| 66 \| 53 \| 52 \| 44 \| 66 \| 64 \| \| D/K \| 12 \| 18 \| 41 \| 32 \| 27 \| 24 \| 22 \|   - Animal-animal gene transfer has a higher acceptance than human-animal across all countries |
| Macer D, Bezar H, Harman N, Kamada H, Macer N (1997)  [63] | Japan, NZ | **Awareness** of specific applications of genetic engineering in species  **Knowledge** on biotechnology  **Attitudes** to genetic engineering medical applications:  - GM mice for medical application  - GM animals (pigs) for organ production   - Risk, moral, usefulness, encouragement | Dichotomous   - Yes/No + D/K - True False + D/K   4-point scale   - From Definitely agree to Definitely disagree | Have you heard of?  *GM mice for cancer research*  *GM pigs for human transplant*    Knowledge of biotechnology:  10 factual statements – responding whether true or false  1. There are bacteria which live from waste water.(TRUE)  2. Ordinary tomatoes do not contain genes, while genetically modified tomatoes do. (FALSE - IMAGE)  3. The cloning of living things produces exactly identical offspring. (IMAGE)  4. By eating a genetically modified fruit, a person's genes could also become modified. (IMAGE)  5. Viruses can be contaminated by bacteria. (FALSE)  6. Yeast for brewing beer consists of living organisms. (TRUE)  7. It is possible to find out in the first few months of pregnancy whether a child will have Down's syndrome.  (TRUE)  8. Genetically modified animals are always bigger than ordinary ones. (FALSE - IMAGE)  9. More than half of the human genes are identical to those with chimpanzees. (TRUE)  10. It is impossible to transfer animal genes into plants. (FALSE - IMAGE)    Opinion on modern biotechnology applications:  For 5 different applications, two on animals  *-  Developing genetically modified animals for laboratory research (mice, cancer).*  *-  Introducing human genes into animals for transplants (pigs, heart)*  *indicate agreement/disagreement*  *a) that you have heard of the application*  that the application...?  *b) benefits society?*  *c) involves risks for society?*  *d) is morally acceptable?*  *e) should be encouraged?* | \|  \| **GM mice for cancer research** \| \| \| \| **GM pigs for human transplant** \| \| \| \| \| --- \| --- \| --- \| --- \| --- \| --- \| --- \| --- \| --- \| \| Variables \| *J* \| \| *NZ* \| \| *J* \| \| *NZ* \| \| \| *a) Awareness* \| 62.6 \| \| 88.3 \| \| 42.6 \| \| 76 \| \| \| **+ agree/ ++ totally agree** \| **++** \| **+** \| **++** \| **+** \| **++** \| **+** \| **++** \| **+** \| \| *b) Useful* \| 27,4 \| 48,3 \| 54,9 \| 29,3 \| 16,2 \| 35,8 \| 29,3 \| 25,7 \| \| *c) Risky* \| 16,2 \| 29,7 \| 22,2 \| 21,2 \| 26,1 \| 32,8 \| 43,6 \| 26,7 \| \| *d) Acceptable* \| 10,4 \| 32,6 \| 24,2 \| 21,4 \| 5,5 \| 18,2 \| 14,9 \| 16,4 \| \| *e) Encouraged* \| 22,9 \| 41,5 \| 30,3 \| 24 \| 14 \| 34,2 \| 18 \| 14,3 \|   - NZ respondents are more aware of both applications than Japan ones  - GM pigs are the least acceptable application despite majority of Japan public finding it useful and to be encouraged although risky and not morally acceptable. As for NZ public, they share the views on the usefulness but find it even riskier, more unacceptable and not to be encouraged  - GM mice for cancer research are less risky, more useful and more to be encouraged by majority of both publics, although Japanese are more enthusiastic. They both find it average morally acceptable   \|  \| **% of correct answers** \| \| \| \| \| --- \| --- \| --- \| --- \| --- \| \|  \| *Survey results* \| \| *Previous studies* \| \| \| Q. \| *J* \| *NZ* \| *C* \| *US* \| \| 1 \| 85,9 \| 98,4 \| 95 \| 84 \| \| 2 \| 47,8 \| 51,1 \| 52 \| 35 \| \| 5 \| 13,1 \| 21,7 \| 18 \| 19 \| \| 6 \| 55,2 \| 86,8 \| 77 \| 68 \| \| 7 \| 57,4 \| 81 \| 77 \| 81 \| \| 8 \| 59,2 \| 52,8 \| 54 \| 36 \| \| 9 \| 37,4 \| 43,1 \| 53 \| 51 \| \| 10 \| 23,3 \| 29,6 \| 38 \| 27 \|   - NZ are the ones who score best overall on biotechnology questions, despite Canadians scoring higher on statements concerning genetics specifically, while Japan public present the lowest general and particular knowledge  - US public lie somewhere in between revealing an average to high knowledge nonetheless, close to Canada and NZ ones |
| Norton J, Lawrence G, Wood G (1998)  [52] | AU | Consumer **attitudes** to genetically-engineered foods | Numerical 6-point scale  • From Strongly Agree (1) to Strongly Disagree (6);  Dichotomous  • Conventional vs genetic eng | For 7 genetically engineered products, two animals/animal-derived   - *pork containing gene sequence of human origin* - *sheep genetically-engineered to be resistant to blowfly strike*   indicate whether:  the genetic engineering of this product was acceptable  *- release of this product will not cause environmental damage*  *- eating this product will have long term health effects*  *- the consumer would be worried about eating the product*  *- the benefits of the product outweigh the risks.*  *- the respondent would like to see the product on the market.* | \| *Acceptability* \| *Mean* \| \| --- \| --- \| \| GE animals \| 4.03 \| \| GE humans \| 4.75 \| \| GE organisms release \| 4.26 \| \| GE sheep \| 2.94 \| \| GE pork \| 4.26 \|   **1 – Strongly agree**  **6- Strongly disagree**  - GE animals are more acceptable than GE humans and release of such organisms is mostly not seen as acceptable  - GE sheep to resist a disease is by far more acceptable than a pork containing a human gene   \| **GE organism** \| *Release* \| *Long-terms health effects* \| *Consume* \| *Buy (Products from)* \| *Benefits vs risks* \| \| --- \| --- \| --- \| --- \| --- \| --- \| \| *GE sheep* \| 3.01 \| - \| 4.04 \| 2.5 \| 3.16 \| \| *GE pork* \| 3.69 \| 4 \| 4.32 \| 4.34 \| 4.15 \|   - GE sheep is in general seen as more acceptable in regards to any of the conditions asked  - GE pork may lead to environmental problems when released and presents more risks than benefits overall. Australian public is also not so inclined to consume it or buy products derived from it. |
| Hampel J, Pfenning U, Peters HP (2000)  [51] | Germany | **Attitudes** towards genetic engineering applications  - Approval, Rejection, Ambivalence   - Medical - Agricultural   **Assessment** of genetic engineering in general  - Positive, Negative, Ambivalent  - Benefit and risk assessment  - Legal/Regulation  - Ethical  **Subjective** knowledge on genetic engineering | 5-point scale  • From Considered Very good to Considered Very bad + no opinion (5);  3 point-scale   - Approval, Ambivalence, Rejection (3); - Positive, Negative, Ambivalent (3);   4-point scale  • From Very good to Very bad (4)   - Very good to completely ignorant | Attitudes of 10 concrete applications of genetic engineering were surveyed, including:  *- Laboratory Animals*  *- Gene transfer between animal species*  *- Genetic therapy (cancer)*      Measuring self-reported knowledge of respondents  Including considerations of risks and benefits   - TABLE 1. Assessment of genetic engineering applications by persons interviewed with positive, ambivalent and negative overall assessment of genetic engineering   Subjective knowledge | - Two-fifths have an ambivalent vision on genetic engineering whereas one-fifth approve it or reject it conditionally and a very low fraction (mere 7 percent) are really opponents to it.  - Ambivalence comes from balanced views on chances and risks where one-third think that risks will outweigh chances and only a fifth think the opposite   \| **Lab animals for pharma research** \| \| *Assessment of genetic engineering applications* \| \| \| \| --- \| --- \| --- \| --- \| --- \| \|  \| **Atittude** \| *Approval* \| *Ambivalence* \| *Rejection* \| \| *Balancing assessment of genetic engineering* \| *Approval* \| 27 \| 31 \| **43** \| \| *Ambivalence* \| 15 \| 25 \| **61** \| \| *Rejection* \| 4 \| 14 \| **82** \|  \| **Breeding transgenic animal species** \| \| *Assessment of genetic engineering applications* \| \| \| \| --- \| --- \| --- \| --- \| --- \| \|  \| **Atittude** \| *Approval* \| *Ambivalence* \| *Rejection* \| \| *Balancing assessment of genetic engineering* \| *Approval* \| 14 \| 19 \| **67** \| \| *Ambivalence* \| 2 \| 8 \| **90** \| \| *Rejection* \| 1 \| 2 \| **97** \|      \| **Cancer genetic therapy** \| \| *Assessment of genetic engineering applications* \| \| \| \| --- \| --- \| --- \| --- \| --- \| \|  \| **Atittude** \| *Approval* \| *Ambivalence* \| *Rejection* \| \| *Balancing assessment of genetic engineering* \| *Approval* \| **91** \| 7 \| 2 \| \| *Ambivalence* \| **78** \| 18 \| 4 \| \| *Rejection* \| **44** \| 32 \| 24 \|   - Both applications concerning animals are rejected in their majority by respondents from Germany even by the ones who approve of genetic engineering  - Application of cancer genetic therapy is highly approved by respondents and even by the majority of genetic engineering rejecters   \| **Assessment** \| *Very good* \| *Fairly good* \| *Partly good* \| *Fairly bad* \| *Very bad* \| *No opinion* \| \| --- \| --- \| --- \| --- \| --- \| --- \| --- \| \| *Laboratory animals* \| 4 \| 10,5 \| 23,4 \| 18 \| 43,3 \| 1 \| \| *Gene transfer between animals* \| 1,7 \| 3,7 \| 10,1 \| 17,4 \| 56,4 \| 0,8 \| \| *Gene therapy (Cancer)* \| 38 \| 31,9 \| 18,2 \| 5,3 \| 3,6 \| 3 \|   - The two applications involving animals are both seen as very bad by subjects in Germany  - On the other hand, cancer genetic therapy is assessed majorly as good by total of 70% respondents  - Gene transfer involving animal species for agricultural purposes is rejected by more than four-fifths  - Almost two-thirds of subjects interviewed consider themselves ignorant on genetic engineering with supporters evaluating their self-knowledge as very good often than opponents, where more than two-thirds see risks outweighing chances  - Ethical issues are considered very important by more than half of respondents, being majorly mentioned by supporters than opponents  - Rejection of technology cannot however be attributed to lack of knowledge on genetic engineering |
| Macer DRJ, Ng MAC (2000)  [55] | Japan | **Awareness** to genetic engineering  **Attitudes** to genetic engineering applications | - Qualitative   3-point scale   - Approve, disapprove + D/K | *“What comes to mind when you think about modern biotechnology*  *in a broad sense, that is, including genetic engineering?”*  “If there were no direct risk to humans and only very remote risks to the environment, would you approve or disapprove of the environmental use of genetically engineered organisms designed to produce ...?”  *… Healthier meat*  *… Cows that produce more milk* | \| **What do you think about genetic engineering?** \| *1997* \| *2000* \| \| --- \| --- \| --- \| \| *GM food* \| 5 \| 17 \| \| *Genetic testing* \| 1 \| 3 \| \| *Gene therapy* \| 3 \| 11 \| \| *GM animals* \| 4 \| 3 \| \| …. \|   - GM food and gene therapy are more related with modern biotechnollgy including genetic engineering than genetic testing and GM animals   \| **Genetic engineering of pigs and cows** \| *1993* \| \| *2000* \| \| \| --- \| --- \| --- \| --- \| --- \| \| *Would you…* \| *Approve* \| *Disapprove* \| *Approve* \| *Disapprove* \| \| *Healthier meat* \| 57 \| 26 \| 52 \| 33 \| \| *Cows that produce more milk* \| 44 \| 32 \| 42 \| 40 \|   - GM pig to obtain healthier meat is more approved than cows to produce more milk in both 1997 and 2000 |
| Magnusson MK, Hursti UKK  (2002)  [53] | Sweden | **Attitudes** towards genetic engineering of some species (pork and salmon) for food consumption  **Knowledge** about biology and genetics | 6-point unipolar scale (X represents reason)  • From “X” at all (1) to Very much “X” (6);  • From No or Not at all to Very strong or Very much … (6);  Dichotomous   - True/False + D/K;   7-point bipolar scale  • Disagree strongly, agree strongly (endpoints) and midpoint: neither agree or disagree (7) | Subjects were asked to rate nine applications of GE in food production, including two on animals   - *Pigs engineered to produce less fatty meat* - *Salmons engineered to grow 10 time faster*   in terms of “desirable” features such as:  Benefit  Good purpose  Necessary  Knowledge consequences  Healthy  Control consumption  and “problematic” features:  Unethical  Reluctance  Concern  Tampering nature  Risk   - Respondents were asked to answer to 5 statements about biology   Conventional food do not contain genes  Yeast used for brewing beer contains living organisms  GM animals are always bigger than “conventional” animals  >50% of the genes are identical for man and chimpanzee  All human cells contain DNA  Subjects scoring on NPI and GHI given by Roininen et al, 1999 Health Scale based on statements like:  *``I would feel guilty if I consumed foods derived from GE'',* *``Consuming foods derived from GE goes against my principles''*,  *``It would be morally wrong for me to consume foods derived from GE''* (Saba et al., 2000),  *``I would purchase GM foods if they (1) tasted better, (2) were healthier than conventional foods, (3) contributed to improve the general state of the environment, and (4) were cheaper''*.  were asked to evaluate constructs such as:   - *Benefit* - *Risk* - *Health* - *Concern*   In regards to   - *Pigs engineered to produce less fatty meat* - *Salmons engineered to grow 10 time faster* | \| **Features** \| *GE pork* \| \| \| \| *GE salmon* \| \| \| \| --- \| --- \| --- \| --- \| --- \| --- \| --- \| --- \| \| ***“Desirable” features*** \| *No* \| *Little* \| \| *High* \| *No* \| *Little* \| *High* \| \| *Benefit* \| 75 \| 20 \| 5 \| \| 82 \| 15 \| 3 \| \| *Good purpose* \| 77 \| 18 \| 5 \| \| 84 \| 12 \| 4 \| \| *Necessary* \| 85 \| 14 \| 1 \| \| 91 \| 8 \| 1 \| \| *Knowledge consequences* \| 77 \| 20 \| 3 \| \| 79 \| 18 \| 3 \| \| *Healthy* \| 76 \| 16 \| 8 \| \| 84 \| 11 \| 5 \| \| *Control consumption* \| 70 \| 21 \| 9 \| \| 73 \| 18 \| 9 \| \| ***“Problematic” features*** \| *No* \| *Little* \| \| *High* \| *No* \| *Little* \| *High* \| \| *Unethical* \| 11 \| 18 \| 71 \| \| 12 \| 14 \| 74 \| \| *Reluctance* \| 7 \| 17 \| 76 \| \| 6 \| 15 \| 79 \| \| *Concern* \| 7 \| 23 \| 70 \| \| 5 \| 22 \| 73 \| \| *Tampering nature* \| 4 \| 16 \| 80 \| \| 3 \| 14 \| 83 \| \| *Risk* \| 5 \| 24 \| 72 \| \| 3 \| 14 \| 83 \| \| *Risk misuse* \| 4 \| 21 \| 75 \| \| 4 \| 20 \| 76 \| \| *Profit use* \| 4 \| 19 \| 78 \| \| 3 \| 10 \| 87 \|   - Sweden public scores high for %No/Very little/Not at all/Very little extent in all “desirable” features and low in every “problematic” constructs  - Indeed GE pork and GE salmon are the 2 applications rated as worst by Sweden public where lowest benefits, highest risks, lowest health and highest bad consequences and intentions are voiced by respondents   \| **Statements** \| *% correct answers* \| *DK* \| \| --- \| --- \| --- \| \| *Conventional food do not contain genes* \| 67 \| 23 \| \| *Yeast used for brewing beer contains living organisms* \| 84 \| 14 \| \| *GM animals are always bigger than “conventional” animals* \| 52 \| 19 \| \| *>50% of the genes are identical for man and chimpanzee* \| 66 \| 28 \| \| *All human cells contain DNA* \| 94 \| 5 \|   - More than the majority (52-84%) of Sweden public is knowledgeable about biology and genetics   \| **Variables** \| *Low NPI* \| \| *High NPI* \| \| *Low GHI* \| \| *High GHI* \| \| \| --- \| --- \| --- \| --- \| --- \| --- \| --- \| --- \| --- \| \| **Constructs** \| *GE pork* \| *GE salmon* \| *GE pork* \| *GE salmon* \| *GE pork* \| *GE salmon* \| *GE pork* \| *GE salmon* \| \| *Benefit* \| 2.4 \| 2.3 \| 1.7 \| 1.4 \| - \| 2.0 \| - \| 1.6 \| \| *Risk* \| 4.3 \| 4.4 \| 1.1 \| 1.0 \| 4.6 \| 4.7 \| 5.1 \| 5.2 \| \| *Healthy* \| 2.6 \| 2.1 \| 1.6 \| 1.5 \| 2.3 \| - \| 1.8 \| - \| \| *Concern* \| 3.9 \| 4.1 \| 5.2 \| 5.3 \| 4.3 \| 4.4 \| 5 \| 5.2 \|   (Scores between 1= and 6)  - High NPI scorers show lower levels of benefits but also of risks for both GE pork and GE salmon  - Low NPI and High GHI scorers are more negative towards GE pork and GE Salmon in terms of its risk despite low GHI scorers being also somewhat negative towards it  - Low NPI scorers also perceive more benefits than High NPI ones for both applications |
| Hallman WK, Adelaja AO, Schilling BJ, Lang JT (2002)  [60] | US | **Awareness** of: Genetic modification;  Genetic engineering;  Biotechnology  **Familiarity** with biotechnology  **Knowledge** on food biotechnology and genetics  **Acceptance** of genetic modification in animals and hybrid animals | 5-point scale   - A great deal, Some or a fairly amount, not much or very little, nothing + Not sure (5); - From Totally Agree to Totally Disagree + Not sure (5); - Morally wrong, not wrong, depends, not sure, refused (5);   4-point scale  • From Strongly approve to Strongly disapprove + Not sure (4);  Dichotomous  • Approve vs Disapprove + Not sure; | Subjects were asked to rate own awareness of:   - *genetic modification, genetic engineering and biotechnology* - *Self-reported understanding of science and technology*   Subjects were asked about knowledge on biotechnology and genetics  *1.There are bacteria which live from waste water.*  *2.Ordinary tomatoes do not contain genes, while genetically modified tomatoes do.*  *3. If a person eats a genetically modified fruit, their genes could also become modified.*  *4. Father's genes that determine whether a child is a girl*  *5. Yeast for brewing beer consists of living organisms.*  *6. Genetically modified animals are always bigger than ordinary ones.*  *7. It is not possible to transfer animal genes into plants.*  *8. Tomatoes genetically modified with genes from catfish would probably taste fishy*  *9. Genetically modified foods are created using radiation to create genetic mutations*  Opinion on biotechnology for animal breeding:  Hybrid animals can be created by genetic engineering / biotechnology: taking parts of the genes of one animal and inserting into cells   - *Familiarity with the method* - *General approval/disapproval* - *Morally wrong?* - *Crossbreeding vs GM* - *GM sheep for milk production (medical application)* | \| **Awareness** \| *A great deal* \| *Some* \| *Not much* \| *Nothing at all* \| *Not sure* \| \| --- \| --- \| --- \| --- \| --- \| --- \| \| *Genetic modification* \| 13 \| 47 \| 29 \| 11 \| <1 \|  \| **Adequately informed** \| *Totally agree* \| *Mostly agree* \| *Mostly disagree* \| *Totally disagree* \| *Not sure* \| \| --- \| --- \| --- \| --- \| --- \| --- \| \| *Biotechnology* \| 14 \| 27 \| 33 \| 24 \| 2 \|   - Almost half of US respondents heard some about genetic modification methods to be used in plants and animals  - A similar percentage feels adequately informed about biotechnology despite the majority represented by one-third mostly disagreeing that are adequately informed on this   \| **Knowledge (Quiz)** \| *% correct answers* \| *D/K* \| \| --- \| --- \| --- \| \| 1 \| 94 \| 2 \| \| 2 \| 57 \| 19 \| \| 3 \| 68 \| 11 \| \| 4 \| 64 \| 9 \| \| 5 \| 70 \| 11 \| \| 6 \| 59 \| 11 \| \| 7 \| 51 \| 16 \| \| 8 \| 67 \| 10 \| \| 9 \| 46 \| 20 \|   - More than the majority of US respondents seem to be have a good knowledge on genetics and genetic modification (Average close two-thirds)  - US public is usually knowledgeable on topics about genetics (between 50 and 100% answers correct), but only two-fifths answered more than 6 questions correctly  - Their own understanding is rated as, at least good, by two-thirds of them   \| **Approval** \| *Strongly/Totally approve* \| *Somewhat/Mildly approve* \| *Somewhat/Mildly disapprove* \| *Strongly/Totally disapprove* \| *Not sure* \| \| --- \| --- \| --- \| --- \| --- \| --- \| \| *Hybrid animals* \| 7 \| 21 \| 25 \| 43 \| 5 \| \| *After Heard* \| 31.7 \| \| 65.3 \| \| 3 \| \| *After not heard* \| 22 \| \| 71.2 \| \| 6.6 \| \| *GM* \| 28 \| \|  \| \|  \| \| *Crossbreeding* \| 31 \| \|  \| \|  \| \| *GM sheep milk* \| 39.5 \| 35.6 \| 9.1 \| 12.5 \| 3.3 \|   - Only less than a third approve of GM to creation of hybrid animals and from the ones that heard about this, close to two-thirds disapprove of it, a fraction that increases with lack of awareness on the topic  - Approval for crossbreeding is higher than for GM but despite by a mere percentage  - As for GM sheep to use milk for medicines and vaccines, the majority of Americans approve of it, close to three-quarters.  - Close to three-fifths of US see GM as morally wrong and a similar number think that GM foods will bring benefits and unjustified fears blocked such developments  - More than a third agree that GM shouldn’t be known at all and less than half think is not necessary |
| European Comission Directorate General for Research  Eurobarometer 58.0 (2002)  [40] | EU15 | **Awareness** of using transgenic animals for xenotransplantation  **Knowledge** on biotechnology and genetic engineering  **Attitudes** to transgenic animals for xenotransplantation | 3-point scale  4-point scale 🡪 Numerical scale (1-4)  • Definitely agree, tend to agree, tend to  disagree and definitely disagree (4)🡪 +1,5 🡪 -1,5  ++ Strong support,  + Weak support,  - Weak opposition  -- Strong opposition  Dichotomous:   - Yes/No | *- Have you heard of this application of biotechnology before, or not?*   - Yes/No   Knowledge of biotechnology:  10 factual statements – responding whether true or false  1. There are bacteria which live from waste water.  2. Ordinary tomatoes do not contain genes, while genetically modified tomatoes do.  3. The cloning of living things produces exactly identical offspring.  4. By eating a genetically modified fruit, a person's genes could also become modified.  5. It is the father’s [1999] / mother's [2002] genes that determine whether a child is a girl  6. Yeast for brewing beer consists of living organisms.  7. It is possible to find out in the first few months of pregnancy whether a child will have Down's syndrome.  8. Genetically modified animals are always bigger than ordinary ones.  9. More than half of the human genes are identical to those with chimpanzees  10. It is impossible to transfer animal genes into plants.  11. Criminal tendencies are mainly genetically inherited  12. Musical abilities are mainly learned    Opinion on modern biotechnology applications:  For 6 different applications, one on animals  -  Xenotransplantation  indicate agreement/disagreement  *a) that you have heard of the application*  that the application...?  *b) benefits society?*  *c) involves risks for society?*  *d) is morally acceptable?*  *e) should be encouraged?*    - by supporters  - by risk-tolerant supporters  - by opponents | \| **Statements** \| *1996* \| *1999* \| *2002* \| \| --- \| --- \| --- \| --- \| \| 1. There are bacteria which live from waste water. \| 83 \| 83 \| 84 \| \| 2. Ordinary tomatoes do not contain genes, while genetically modified tomatoes do. \| 35 \| 35 \| 36 \| \| 3. The cloning of living things produces exactly identical offspring. \| 46 \| 64 \| 66 \| \| 4. By eating a genetically modified fruit, a person's genes could also become modified. \| 48 \| 42 \| 49 \| \| 5. It is the father’s [1999] / mother's [2002] genes that determine whether a child is a girl \| N/A \| 44 \| 53 \| \| 6. Yeast for brewing beer consists of living organisms. \| 68 \| 66 \| 63 \| \| 7. It is possible to find out in the first few months of pregnancy whether a child will have Down's syndrome. \| 81 \| 79 \| 79 \| \| 8. Genetically modified animals are always bigger than ordinary ones. \| 36 \| 34 \| 38 \| \| 9. More than half of the human genes are identical to those with chimpanzees \| 51 \| 48 \| 52 \| \| 10. It is impossible to transfer animal genes into plants. \| 27 \| 26 \| 26 \|   - Questions on genetics (2, 4, 8, 9, 10) reveal a lower knowledge than questions concerning biology in specific which are answered consistently correctly 1996-2002 period.   \| *%* \| *Talked frequently* \| *Public discussions* \| *TV programme/Read* \| *Applications heard (2-3)* \| *Engaged* \| \| --- \| --- \| --- \| --- \| --- \| --- \| \| *EU15* \| 32 \| 33 \| 68 \| 1.79 \| 25 \|   - Awareness in Europe is low in terms of speaking about biotechnology and engagement  - Nevertheless, more than two-thirds watched a TV program or read about it and on average Europeans heard about 2 of 3 applications on biotechnology asked   \| **Judgements** \| *Introducing human genes to produce organs for human transplants (-1,5 to 1,5)* \| \| --- \| --- \| \| *Useful* \| ~0.3* \| \| *Risky* \| ~0.25* \| \| *Morally acceptable* \| ~0.05* \| \| *Encouraged* \| ~0.1* \|   *averages are approximated  - Xenotransplantation is seen as average useful and risky at the same time despite being only little encouraged and ambivalent on moral acceptance   \|  \| *Introducing human genes to produce organs for human transplants* \| \| \| --- \| --- \| --- \| \| **Public (~half sample)** \| *1996* \| *2002* \| \| Supporters \| 23 \| 29 \| \| Risk tolerant supporters \| 33 \| 44 \| \| Opponents \| 45 \| 27 \|   - Number of supporters increased from 1996 to 2002 as well as risk tolerant ones and concomitantly opponents also decreased markedly |
| Inaba M, Macer DRJ (2003)  [56] | Japan | **Awareness** of biotechnology, gene therapy, genetic engineering  **Attitudes** genetic engineering  **Attitudes** to genetic modification of animals for certain applications  **Attitudes** towards gene therapy in germline and somatic cells | 3-point scale   - Not heard, Heard of it, Could explain to a friend (3)   Dichotomous   - Yes/No + D/K   4-point scale •  • From Definitely Agree to Definitely Disagree + D/K (4)   - No, few, some, a lot | Familiarity with:   - *Biotechnology* - *Gene therapy* - *Genetic engineering*       Opinion on modern biotechnology applications:  -  *Introducing human genes into animals for transplants (pigs, heart)*  indicate agreement/disagreement that the application...?  *a) is familiar to the respondent?*  *b) is useful for society*  *c) involves risks for society?*  *d) is morally acceptable?*  *e) should be encouraged?*    - Using genetic engineering to make mosquitoes unable to be a vector for human diseases   - Provided no risk to humans and remote risks to environment, approve/disapprove of environmental release of the genetically engineered:   - *Produce healthier meat* - *Cows which produce more milk* - *Mosquitoes which do not transmit human disease* - Q28. *How do you feel about scientists changing the genetic makeup of human cells* to:   a. *Cure a usually fatal disease, such as cancer*  b. *Reduce the risk of developing a fatal disease later in life*  c. *Prevent children from inheriting a usually fatal disease*  d. *Prevent children from inheriting a non-fatal disease*, such as  diabetes  e. *Improve the physical characteristics that children would inherit*  f. *Improve the intelligence level that children would inherit*  g. *Make people more ethical*  h. *As an AIDS vaccine* | \|  \| *1991* \| \| \| *1993* \| \| \| *2003* \| \| \| \| --- \| --- \| --- \| --- \| --- \| --- \| --- \| --- \| --- \| --- \| \| **N – Not heard**  **H – Heard**  **E - Explain** \| *N* \| *H* \| *E* \| *N* \| *H* \| *E* \| *N* \| *H* \| *E* \| \| *Gene therapy* \| - \| - \| - \| 23 \| 59 \| 19 \| 14 \| 61 \| 25 \| \| *Genetic engineering* \| 6 \| 68 \| 26 \| 9 \| 74 \| 17 \| 10 \| 70 \| 20 \| \| *Biotechnology* \| 3 \| 65 \| 32 \| 6 \| 65 \| 29 \| 7 \| 68 \| 25 \|   - Biotechnology is the topic that people heard more about from 1991 to 2003, despite their awareness slight increase for both gene therapy and genetic engineering ones from 1993 to 2003  - As for their literacy, biotechnology is also the topic that respondents feel they can explain better but this percentage only goes from one-quarter to one-fifth by 2003   \|  \| **GM pigs for human transplant** \| \| \| \| \| \| \| --- \| --- \| --- \| --- \| --- \| --- \| --- \| \| *Variables* \| *1997* \| \| *2000* \| \| *2003* \| \| \| *a) Awareness* \| 42.6 \| \| 66.8 \| \| 61.6 \| \| \| **+ agree/ ++ totally agree** \| **+** \| **++** \| **+** \| **++** \| **+** \| **++** \| \| *b) Useful* \| 35.8 \| 16.2 \| 27.7 \| 13.7 \| 25.2 \| 6.1 \| \| *c) Risky* \| 32.8 \| 26.1 \| 23.4 \| 25.9 \| 34.2 \| 24.4 \| \| *d) Acceptable* \| 18.2 \| 5.5 \| 16.4 \| 7.3 \| 14.3 \| 4.9 \| \| *e) Encouraged* \| 34.2 \| 14 \| 18.3 \| 11 \| 20.8 \| 5.5 \|   - Only less than half were aware of GM pigs for organ production with transplantation purposes, a fraction that rose to two-thirds and remained in above three-fifths in 2003  - Consistently, usefulness decreased from half to a third of agreement by Japanese public  - Risk behaved differently: in 1997, more than half felt there was risk; in 2000, less than half and in 2003, close to three-fifths  - GM pigs were seen as something to encourage by almost half in 1997, less than a third by 2000 and by a quarter in 2003  - Moral acceptance was consistently low, around one-fifth along time  - Reasons pointed by respondents to justify opinions:  - human benefits, balanced view, risk, ethical issues, necessity, naturalness and personal choice  - For xenotransplantation, safety was pointed by two-fifths of respondents as a major risk including: infectious diseases, GMO safety and transmission of genetic changes followed by ethical issues pointed by close to a third, including animal rights   \|  \| **Approval** \| \| \| \| \| \| \| --- \| --- \| --- \| --- \| --- \| --- \| --- \| \| *Years* \| *1997* \| \| *2000* \| \| *2003* \| \| \| *Answers* \| *Yes* \| *DK* \| *Yes* \| *DK* \| *Yes* \| *DK* \| \| *GM pigs for meat less fatty* \| 57 \| 17 \| 51.6 \| 33 \| 52 \| 18 \| \| *GM cows to produce more milk* \| 44 \| 24 \| 42.1 \| 18.3 \| 37 \| 28 \| \| *Mosquitoes to not transmit disease to humans* \| - \| - \| - \| - \| 53 \| 27 \|   - GM pigs for meat less fatty had consistent acceptance over the years in levels above half of the public  - GM cows to produce more had consistently less acceptance over the years going from roughly two-fifths to roughly one-third  - GM mosquitoes to avoid human disease transmittance had acceptance levels in above half of the public   \| **Cure a usually fatal disease** \| *1993* \| *2000* \| *2003* \| \| --- \| --- \| --- \| --- \| \| *Strongly agree* \| 42 \| 38.3 \| 39.3 \| \| *Somewhat agree* \| 31 \| 34.4 \| 43.2 \| \| *Somewhat agree* \| 3 \| 13.1 \| 5.0 \| \| *Strongly agree* \| 2 \| 4.6 \| 2.2 \| \| **Reduce the risk of developing a fatal disease later in life** \| *1993* \| *2000* \| *2003* \| \| *Strongly agree* \| 35 \| 28.4 \| 40.5 \| \| *Somewhat agree* \| 40 \| 34.8 \| 40.3 \| \| *Somewhat agree* \| 5 \| 16.7 \| 4.1 \| \| *Strongly agree* \| 1 \| 6 \| 2.2 \| \| **Improve the physical characteristics that children would inherit** \| 1993 \| 2000 \| 2003 \| \| *Strongly agree* \| 12 \| 10.7 \| 11.4 \| \| *Somewhat agree* \| 16 \| 12.5 \| 17.2 \| \| *Somewhat agree* \| 35 \| 43.4 \| 34.9 \| \| *Strongly agree* \| 16 \| 19.9 \| 14.4 \| \| **Improve intelligence level that children would inherit** \| *1993* \| *2000* \| *2003* \| \| *Strongly agree* \| 13 \| 9.6 \| 10.4 \| \| *Somewhat agree* \| 13 \| 10.3 \| 16.5 \| \| *Somewhat agree* \| 35 \| 45.2 \| 34.2 \| \| *Strongly agree* \| 49 \| 22.4 \| 15.7 \| \| **Make people more ethical** \| *1993* \| *2000* \| *2003* \| \| *Strongly agree* \| 14 \| 10.4 \| 10.6 \| \| *Somewhat agree* \| 10 \| 8.2 \| 13.4 \| \| *Somewhat agree* \| 32 \| 33.7 \| 32.9 \| \| *Strongly agree* \| 21 \| 30.1 \| 17.5 \|   - Cure and reduce risk of developing fatal disease are more approved (~40%) than improvement of physical or intelligence characteristics (27%) on average |
| Inaba M, Macer DRJ (2003b)  [64] | Japan | **Awareness** of GM pigs for xenotransplantation  **Approval** of GM animal applications | - Dichotomous   - Yes/No + D/K   - 4-point scale   - From Definitely agree to definitely disagree + D/K   - Qualitative | Opinion on modern biotechnology applications:  -  *Introducing human genes into animals for transplants (pigs, heart)*  indicate agreement/disagreement that the application...?  *a) is familiar to the respondent?*  *b) is useful for society*  *c) involves risks for society?*  *d) is morally acceptable?*  *e) should be encouraged?*  - Reasons given regarding  *a) GM pigs for human transplants*  *b) GM mice for cancer research*  in terms of …  *… human benefits*  *… balanced view*  *… risk concerns*  *… ethical concerns*  *… we don’t need*  *… unnatural feelings*  *… consumer’s choice* | \|  \| **GM pigs for human transplant** \| \| \| \| \| \| \| --- \| --- \| --- \| --- \| --- \| --- \| --- \| \| *Variables* \| *1997* \| \| *2000* \| \| *2003* \| \| \| *a) Awareness* \| 42.6 \| \| 66.8 \| \| 61.6 \| \| \| **+ agree/ ++ totally agree** \| **+** \| **++** \| **+** \| **++** \| **+** \| **++** \| \| *b) Useful* \| 35.8 \| 16.2 \| 27.7 \| 13.7 \| 25.2 \| 6.1 \| \| *c) Risky* \| 32.8 \| 26.1 \| 23.4 \| 25.9 \| 34.2 \| 24.4 \| \| *d) Acceptable* \| 18.2 \| 5.5 \| 16.4 \| 7.3 \| 14.3 \| 4.9 \| \| *e) Encouraged* \| 34.2 \| 14 \| 18.3 \| 11 \| 20.8 \| 5.5 \|   - Only less than half were aware of GM pigs for organ production with transplantation purposes, a fraction that rose to two-thirds and remained in above three-fifths in 2003  - Consistently, usefulness decreased from half to a third of agreement by Japanese public  - Risk behaved differently: in 1997, more than half felt there was risk; in 2000, less than half and in 2003, close to three-fifths  - GM pigs were seen as something to encourage by almost half in 1997, less than a third by 2000 and by a quarter in 2003  - Moral acceptance was consistently low, around one-fifth along time  - Human benefits are the most evoked reason by Japan citizens in terms of usefulness for GM pigs for human transplant but the number of them decreased in 3 years’ time  - For risk parameter, risk concerns obviously dominated and also increased from 2000 to 2003  - For moral acceptance, the main reason mentioned was the unnecessity of such application in 2000 and this opinion changed in 2003 with citizens evoking unnatural feelings and ethical concerns (23%) and human benefits at the same time (27%)  - Finally, for social acceptability, the main reasons mentioned in 2000 were similar to moral acceptance in 2003 but human benefits prevailed in 2003 with one-third saying this is the reason for GM pigs to be used for transplants   \|  \| **GM pigs for human transplant** \| \| \| \| \| \| \| \| \| --- \| --- \| --- \| --- \| --- \| --- \| --- \| --- \| --- \| \|  \| *2000* \| \| \| \| *2003* \| \| \| \| \| *Reasons* \| *U* \| *R* \| *MA* \| *SA* \| *U* \| *R* \| *MA* \| *S A* \| \| *Stated* \| 152 \| 162 \| 159 \| 159 \| 232 \| 260 \| 235 \| 248 \| \| *Human benefits* \| 55 \| 14 \| 24 \| 46 \| 44 \| 9 \| 27 \| 32 \| \| *Balanced view* \| 13 \| 11 \| 13 \| 12 \| 8 \| 6 \| 13 \| 14 \| \| *Risk concerns* \| 23 \| 41 \| 8 \| 15 \| 15 \| 50 \| 3 \| 9 \| \| *Ethical concerns* \| 10 \| 30 \| 25 \| 16 \| 8 \| 23 \| 23 \| 19 \| \| *We don’t need* \| 10 \| - \| 30 \| 22 \| 7 \| - \| 13 \| 12 \| \| *Unnatural feelings* \| 28 \| 26 \| 25 \| 22 \| 17 \| 18 \| 23 \| 16 \| \| *Consumers choice* \| 2 \| - \| - \| - \| 2 \| - \| - \| - \|  \|  \| **GM mice for cancer research** \| \| \| \| \| --- \| --- \| --- \| --- \| --- \| \|  \| *2000* \| \| \| \| \| *Reasons* \| *U* \| *R* \| *MA* \| *SA* \| \| *Stated* \| 153 \| 142 \| 135 \| 138 \| \| *Human benefits* \| 81 \| 15 \| 43 \| 64 \| \| *Balanced view* \| 16 \| 29 \| 26 \| 19 \| \| *Risk concerns* \| 12 \| 38 \| 8 \| 27 \| \| *Ethical concerns* \| 14 \| 31 \| 28 \| 4 \| \| *We don’t need* \| 1 \| - \| 19 \| 21 \| \| *Unnatural feelings* \| 12 \| 14 \| 12 \| 5 \| \| *Consumers choice* \| 0.8 \| - \| - \| 6 \|   - In GM mice for cancer research, the main reason mentioned for usefulness, moral acceptability and social acceptability by citizens in 2000 were human benefits  - For risk, risk and ethical concerns were mentioned |
| Hallman WK, Hebden WC, Aquino HL, Cuite CL, Lang, JT (2003)  [61] | US | **Awareness and familiarity** with genetic engineering and biotechnology  **Knowledge** about biotechnology, science and genetic modification  **Opinions** on biotechnology, animal-based GM foods and GM foods | 4-point scale   - A great deal, Some or a fairly amount, not much or very little, nothing (4); - From Strongly approve to Strongly disapprove + D/K (4);   Dichotomous   - Yes, No + D/K; - Tend to agree or disagree   Qualitative  6-point scale   - From Extremely Negative to Extremely Positive + D/K (6); | Awareness and familiarity:   - Genetic modification involves new methods for scientists to create new plants/animals by taking parts of the genes of one and inserting into the cells of another plant/animal, sometimes called genetic engineering or biotechnology. *How much have you heard/read about this?* - *Have you heard / read about (list of applications)*     Self-reported knowledge of   - *biotechnology, genetic engineering, or genetic modification* - *science and technology*     Objective knowledge:   - 11 factual statements – responding whether true or false   *There are bacteria which live from waste water.*  *Ordinary tomatoes do not contain genes, while genetically modified tomatoes do.*  *By eating a genetically modified fruit, a person's genes could also become modified.*  *It is the mother's genes that determine whether a child is a girl*  *Yeast for brewing beer consists of living organisms.*  *Genetically modified animals are always bigger than ordinary ones.*  *It is not possible to transfer animal genes into plants.*  *The cloning of living things produces genetically identical copies*  *More than half of the human genes are identical to those with chimpanzees*  *Tomatoes genetically modified with genes from catfish would probably taste fishy*  *Genetically modified foods are created using radiation to create genetic mutations*  Opinion:   - *Acceptability of Biotechnology/genetic engineering/genetic modification acceptable as currently used? If unacceptable, why?* - *Approval/disapproval of genetic modification to create animal based food products* - Effect of genetic modification on people’s quality of life | \|  \| *A great deal* \| *Some* \| *Not much* \| *Nothing* \| \| --- \| --- \| --- \| --- \| --- \| \| GM \| 12 \| 45 \| 29 \| 14 \|  \|  \| *A fair amount* \| *Very little* \| *Nothing* \| \| --- \| --- \| --- \| --- \| \| GE/GM \| 21 \| 55 \| 22 \|   - More than half of US public was aware of GM and similar number think they know “very little” on GE and GM subjects   \| *Statements* \| *US* \| *Europe* \| \| --- \| --- \| --- \| \| There are bacteria which live from waste water. \| 94 \| 84 \| \| Ordinary tomatoes do not contain genes, while genetically modified tomatoes do. \| 57 \| 36 \| \| By eating a genetically modified fruit, a person's genes could also become modified. \| 68 \| 49 \| \| It is the mother's genes that determine whether a child is a girl \| 73 \| 53 \| \| Yeast for brewing beer consists of living organisms. \| 76 \| 63 \| \| Genetically modified animals are always bigger than ordinary ones. \| 57 \| 38 \| \| It is impossible to transfer animal genes into plants. \| 48 \| 26 \| \| The cloning of living things produces genetically identical copies \| 69 \| 6 \| \| More than half of the human genes are identical to those with chimpanzees \| 55 \| 52 \| \| Tomatoes genetically modified with genes from catfish would probably taste fishy \| 60 \| - \| \| Genetically modified foods are created using radiation to create genetic mutations \| 48 \| - \|   - Mean score of the quiz reached close to 65% although more than half of the respondents received a failing grade due to having less than 70% of answers correct  - Nevertheless, US public score better than European public for the same time period   \| **GM animal** \| *Strongly approve* \| *Somewhat approve* \| *Somewhat disapprove* \| *Strongly disapprove* \| *D/K* \| \| --- \| --- \| --- \| --- \| --- \| --- \| \| *2001* \| 7 \| 21 \| 25 \| 43 \| 5 \| \| *2003* \| 6 \| 21 \| 21 \| 45 \| 8 \|   - GM animal is seen as a matter of approval among only less than a third of US public and this pattern didn’t change from 2001.   \| **Acceptability** \| *Extremely negative* \| *Somewhat negative* \| *Neutral* \| *Somewhat positive* \| *Extremely positive* \| *D/K* \| \| --- \| --- \| --- \| --- \| --- \| --- \| --- \| \| *GE* \| 14 \| 10 \| 30 \| 9 \| 5 \| 4 \| \| *GM* \| 16 \| 14 \| 23 \| 7 \| 7 \| 4 \| \| *Biotec* \| 10 \| 11 \| 28 \| 12 \| 9 \| 6 \|   - Biotechnology is the term rated most positively followed by both genetic engineering and genetic modification, despite the latter being seen as slightly more negative even  - Roughly two-thirds agree that would be unhappy if they were served GM food and less than half agreed and disagreed at the same time that they would not mind with eating GM food on a restaurant  - The higher the degree in US citizens the higher the likelihood of accepting animal-based GM  - Subjective knowledge of GM foods and objective knowledge on biotechnology, on the other hand, have a weak correlation with animal-based GM approval despite slight existent  - Healthfulness has a higher correlation with approval of GM foods |
| Puduri V, Govindasamy R, Lang JT, Onyango B (2004)  [62] | US | **Approval** of animal-based GM foods | Dichotomous  • Approve vs Disapprove | Respondents’ opinion about:  approval of plant and animal-based by their:   - *objective knowledge about genetically modified foods.* - *education.* | \|  \| *Education* \| \| \| *Knowledge* \| \| \| \| --- \| --- \| --- \| --- \| --- \| --- \| --- \| \| **Levels** \| High school \| Some/4-years \| Grad \| Low \| Medium \| High \| \| *GM animal approval* \| 23 \| 26 \| 37 \| 21 \| 36 \| 100 \|   - Grad students were the ones more approving of GM animal-based food with high school and 4-years education scoring similarly around one-quarter  - All high scorers on knowledge approved GM animal-derived food, by opposition to one-third and one-fifth among medium and low scorers, respectively  - Overall, GM animal-derived foods are approved by close to a quarter of US public |
| Small BH, Parminter TG, Fisher MW (2005)  [57] | NZ | **Attitudes** to genetic engineering and genetic modification in general and specific to food and medical applications | 3-point scale   - Total support, support in some circumstances, totally opposed + D/K (3);   20-point scale   - From strongly agree (1) strongly disagree (20)   (For ease of presentation of graphic data, the 20-point scale has been collapsed into 5  equal categories where 1-4 =Strongly agree, 5-8 = Agree, 9-12=Neutral, 13-16 = Disagree, and 17-20 = Strongly disagree.) | - *Attitudes to food and medical applications of genetic modification in general (Questions 8-10*). The *latter three questions rated general support for genetic engineering for food, medical, and other uses*, on a 3-point scale - This information was followed by *Questions 11-91* designed to elicit responses on a wide range of issues *surrounding genetic engineering relating primarily to food and medical applications in the New Zealand context, both in general and with respect to the hypothetical product.* | \| **Support*** \| *Strongly agree* \| *Agree* \| *Neutral* \| *Disagree* \| *Strongly disagree* \| *D/K* \| \| --- \| --- \| --- \| --- \| --- \| --- \| --- \| \| *GM cows for human benefit* \| <10 \| 10-15 \| 15 \| 15 \| >40 \| 5-10 \| \| *GE for disease* \| ~5 \| 15 \| 15-20 \| 15 \| 25 \| ~15 \| \| *GE for food* \| 5-10 \| ~15 \| 15-20 \| ~10 \| 30 \| ~15 \| \| *GE is environmentally friendly* \| 0-5 \| ~5 \| ~20 \| ~10 \| 30 \| 25 \| \| **Milk from GE cows** \|  \| \| \| \| \| \| \| *Good about purchase* \| 5-10 \| ~10 \| 22 \| 10 \| 55 \| ~5 \| \| *Useful* \| 10 \| ~15 \| 22 \| 5-10 \| 30-35 \| 10-15 \| \| *Buy if available* \| 5-10 \| ~15 \| 20-25 \| ~15 \| ~40 \| ~0 \|   *- all averages are approximated  - GM cows are not seen with morality for human benefit application with the majority of people (~40%) strongly disagreeing with it, with the usefulness of milk derived from them and with its purchase if available. An even higher proportion was heavily opposed that it would be good to purchase this product.  - In general, the majority of the public from NZ disagrees strongly with genetic engineering for both food, disease and environmental purposes  - GE seen as unacceptable if it harmed:  - people (18.7) > environment (18.3) > animals (17.04) by this order  - GE was supported conditionally in its majority for:  - Medicine (62%) > Food (52%) |
| Nayga RM, Fishera MG, Onyango B (2006)  [58] | South Korea and US | **Awareness** to GM issue and Science  **Knowledge** of science and technology  **Attitudes** to GM technology to have animal-derived products | 5-point scale   - Poor, Fair, Good, Very good, Excellent (5);   4-point scale   - Nothing at all, not much, some, a great deal (4); - From Strongly approve to Strongly disapprove (4); | In both the U.S. and South Korean surveys, respondents were also asked to rate:  Self-reported knowledge of   - *GM methods* - *science and technology*   Awareness of   - *GM methods*   Opinion:   - *Approval/disapproval of genetic modification to create animal based food products* | \| **Knowledge** \| *Poor* \| \| *Fair* \| \| *Good* \| \| *Very good* \| \| *Excellent* \| \| \| --- \| --- \| --- \| --- \| --- \| --- \| --- \| --- \| --- \| --- \| --- \| \| *Country* \| *US* \| *SK* \| *US* \| *SK* \| *US* \| *SK* \| *US* \| *SK* \| *US* \| *SK* \| \| *Science* \| 18 \| 10 \| 42 \| 39 \| 25 \| 32 \| 12 \| 19 \| 3.7 \| 0.5 \|   - Almost 60% of respondents subjectively rank their understanding of Science as poor or fair   \| **Awareness** \| *Nothing at all* \| \| *Not much* \| \| *Some* \| \| *A great deal* \| \| \| --- \| --- \| --- \| --- \| --- \| --- \| --- \| --- \| --- \| \| *Country* \| *US* \| *SK* \| *US* \| *SK* \| *US* \| *SK* \| *US* \| *SK* \| \| *GM animal* \| 9 \| 5 \| 26 \| 11 \| 52 \| 66 \| 12 \| 18 \|   - When it comes to GM animal subject specifically, around half of US and two-thirds of SK respondents heard ‘Some’ about it.   \| **Approval** \| *Strongly approve* \| \| *Somewhat approve* \| \| *Somewhat disapprove* \| \| *Strongly disapprove* \| \| \| --- \| --- \| --- \| --- \| --- \| --- \| --- \| --- \| --- \| \| *Country* \| *US* \| *SK* \| *US* \| *SK* \| *US* \| *SK* \| *US* \| *SK* \| \| *GM animal* \| 5.9 \| 3 \| 22 \| 19 \| 24 \| 40 \| 48 \| 38 \|   - US public in its half majority disapproves strongly GM-animal compared to 40% of South Korean public but overall SK public is more disapproving of it (total of almost 80%) than US public (close to 70%)  - Subjects with higher knowledge have a more likelihood to approve GM animal-derived products  - Individuals that approve GM animal-derived foods have:  - at least good Science and Technology knowledge in US and poor to fair in SK  - accurate knowledge of GM outcomes  - at least heard some about GM |
| Govindasamy R, Onyango B, Hallman WK, Jang H-M, Puduri V (2008)  [59] | South Korea | **Awareness** of GM foods in supermarket  **Knowledge** of Science  **Attitudes** related to GM foods regarding personal health and safety (animal-based) | 4-point scale  • From Strongly approve to Strongly disapprove (4);  Knowledge  3 different levels:   - LOWSCORE (representing less than five correct responses); (b) MIDSCORE (six to eight correct responses); and (c) HIGHSCORE (nine or more correct responses).   Awareness:   - *1 (if aware); 0 if not* | Consumers’ opinion on and knowledge and awareness of GM foods.    Objective measurements of science and biotechnology knowledge.   - *11 questions to measure their basic understanding of science relating to biotechnology*   *There are bacteria which live from waste water.*  *Ordinary tomatoes do not contain genes, while genetically modified tomatoes do.*  *By eating a genetically modified fruit, a person's genes could also become modified.*  *It is the mother's genes that determine whether a child is a girl*  *Yeast for brewing beer consists of living organisms.*  *Genetically modified animals are always bigger than ordinary ones.*  *It is not possible to transfer animal genes into plants.*  *Tomatoes genetically modified with genes from catfish would probably taste fishy*  *Genetically modified foods are created using radiation to create genetic mutations*  *The cloning of living things produces genetically identical copies*  *More than half of the human genes are identical to those with chimpanzees*   - Awareness of GM Foods in Supermarkets. A dummy GM_NOWMA was assigned | - A quarter of respondents approve GM in animals compared to two-fifths that disapprove it  - A lower education brings higher approval of GM among South Koreans which accept it less for animals than plants  - Knowledge of Science is correlated with GM acceptance and close to half are aware that GM foods are available on supermarkets  - Awareness of GM foods impact approval of GM acceptance for GM animal-derived foods |
