# Supplementary material for: What do people think about genetic engineering? A systematic review of questionnaire surveys before and after the introduction of CRISPR
Source: Front Genome Ed. 2023 Dec 19;5:1284547. doi: 10.3389/fgeed.2023.1284547 (PMC10773783; doi:10.3389/fgeed.2023.1284547)
Supplement: Supplementary file 6 [file Table3.DOCX]

| **Authors** | **Item generation** | **Validity** | **Reliability** | **Response rate** | **Sampling** | **Bias** | **Weighing** | **Informed Consent/Incentives/Ethical Statement** |
| --- | --- | --- | --- | --- | --- | --- | --- | --- |
| OTA (1987)  [34] | Yes | Content validity: Questionnaire designed according with results of focus groups held with samples of public and pre-testing for posterior modification  Face validity: Critiques from reviewers | Not mentioned | Sufficient to obtain acceptable sampling error | Selection of househols based on national-area-probability sample (regional stratification and size of place)  Counties as primary sampling units (distribution of the population)  Random selection of telephone numbers from PSUs and random digit dialing    Sampling error at 95% CI  Sampling variance= ±2-3% | Published phone listing as universe is inadequate for telephone surveys – Random digit dialing avoids this    Demographics of achieved samples compared to Census estimates to avoid bias of sample distortions    Data handling and interviewer recording can introduce errors | Non-coverage (nontelephone households) weighted by Census estimates demographics    All findings are from weighted samples | No/No/No |
| Comission of the European Communities Directorate-General Science, Research and Development  (Eurobarometer 35.1) (1991)  [37] | Yes | Yes  Face validity: INRA (Europe) carried fieldwork co-ordinated by ECO  Content validity: Question text and contingency text based on INRA codebook and assists for discrepancies (machine-readable documentation) found can be corrected through a Central Archive    Construct validity: Hypothesis-testing  Convergent validity: Constructed variables for some index or status | Yes  Split-half samples reveals split-half reliability | Different by country | A multi-stage sampling design: 1st: Selection based on administrative regions by proportion of sample size for degree of urbanization.  2nd: a cluster of addresses was selected from 1st stage randomly  3rd: Individual random selection procedure | Not mentioned | Results weighed according to proportion of each nation based on demographics in comparison with Universe from National Research Institutes and EUROSTAT    National weighting based on Universe from NRI and EUROSTAT through geographical distribution by region and degree of urbanisation plus demographics    International weighting done by INRA achieved by comparison with Regional Statistics Yearbook of 1988 from EUROSTAT | No/No/No |
| Macer (1992)  [35] | No (From OTA 1987 and Couchman, Fink-Jensen, 1990) | Previous surveys validation | Not mentioned | 26% | Random nationwide distribution    Sample characteristics similar to population | Mail response had longer time for contemplation and lengthier comments but response rate can be lower than interviews  Sample bias can be obtained and non-responders were not reminded | Not mentioned | No/No/No |
| Comission of the European Community Directorate-General Science, Research and Development Unit XII/E/1  (Eurobarometer 39.1)  (1993)  [38] | No | Yes  Face validity: INRA (Europe) carried fieldwork  Content validity: Question text and contingency text based on INRA codebook and assists for discrepancies (machine-readable documentation) found can be corrected through a ICSPR documentation    Construct validity: Hypothesis-testing  Convergent validity: Constructed variables for some index or status | Yes  Split-half samples reveals split-half reliability | Different by country | A multi-stage sampling design: 1st: Selection based on administrative regions by proportion of sample size for degree of urbanization.  2nd: a cluster of addresses was selected from 1st stage randomly  3rd: Individual random selection procedure | Not mentioned | Results weighed according to proportion of each nation based on demographics in comparison with Universe from National Research Institutes and EUROSTAT    National weighting based on Universe from NRI and EUROSTAT through geographical distribution by region and degree of urbanisation plus demographics    International weighting done by INRA achieved by comparison with Regional Statistics Yearbook of 1989 from EUROSTAT | No/No/No |
| Macer DRJ, Akiyama S, Alora AT, Asada Y, Azariah J, Azariah H, et al, (1995)  [36] | No (Based on Macer 1994; OTA, 1986; Macer, 1992) | Questionnaires were pretested: Content validity | Not mentioned | Representative:  AU= 13%  Japan= 23%  NZ= 22%    Not representative:  India= 40%  Israel= 50%  Russia= 43%  Thailand= 36% | Random nationwide distrbution by hand    Samples chosen by clustered random sampling based on community maps cross-section – random choice of names    No more than 50 questionnaires handed and only 30 per area    Israel sampling was not random and sample size was not sufficient | Open responses to exsmine reasoning in order to avoid bias    Standardized response avoided by avoidance of personal contact during handing-out of questionnaires    AU, NZ, Japan questionnaires delivered by same people to help with standardization    Selection bias introduced (higher education) in India, Israel, Russia and Thailand: English-spoking and sampling through universities, academics, etc.    Indian and Russian surveys are not representative in terms of country areas | Not mentioned | No/No/No |
| Marteau T, Michie S, Drake H, Bobrow M, (1995)  [50] | No (Daily Telegraph survey) | Yes  Content validity: The wording of this item was similar to that used to assess attitudes towards the use of gene manipulation to achieve the same outcomes. | Not mentioned | N/A | Sample taken from 100 electoral constituencies and stratified by demographics and employment status (representative but quota) | Bias introduced by context, namely in terms of media optimism could have influenced shift in reporting | Not mentioned | No/No/No |
| Ng MAC, Takeda C, Watanabe T, Macer D (2000)  [43] | No (Based on Macer 1994; OTA, 1986; Macer, 1992) and Macer, 1997 | Yes  Previous survey validation | Not mentioned | 12% | Random national samples by choosing 3000 random houses across Japan    Sampling error: ±5% | Not mentioned | Not mentioned | No/No/No |
| Macer DRJ, Azariah J, Srinives P (2000)  [44] | No (Based on Macer 1994; OTA, 1986; Macer, 1992 and NZ 1990) | Previous survey validation | Not mentioned | Representative:  AU= 13%  Japan= 23%  NZ= 22%    Not representative:  India= 40%  Israel= 50%  Russia= 43%  Thailand= 36% | Random nationwide distribution | Open responses to examine reasoning in order to avoid bias    Some countries not representative of population sample | Not mentioned | No/No/No |
| UK Human Genetics Comission (2001)  [48] | Yes | Not mentioned | Not mentioned | N/A | Randomly recruitment panel of general public for People’s Panel members    Sampling error: ±2-3% (CI 95%) | Not mentioned | Data weighted to the known profile of UK population by demographics and socioeconomic status    Additional booster interviews to ensure large base sizes | No/No/No, but interview declaration |
| Cook AJ, Fairweather JR, Satterfield T, Hunt LM (2004)  [46] | Yes  199 separate items compared to usual 100 in AERU surveys | Yes  Face and content validity: Questionnaire designed with items developed from focus group research and pre-tested with small group  Construct validity: Items included and minor adjustments were made to questionnaire items    Construct validity also explained in detail using literature examples and similar questions employed in previous surveys | Yes    Internal consistency (Cronbach’s alpha): Construction of attitude measure for correlation with other variables | 36.3% | Random distribution to selected addresses    Over-representation and under-representation of some fractions of the public (Compared to Census) | Definition provided at the beginning could have led to response bias    Response bias found by representativeness tests: Over-representation of older people and higher levels of income, education and ethinicity    Non-response bias issue investigated by randomly surveying non-responders    Bias derived from lack of knowledge    No gender bias | Not mentioned | No, but inviting  voluntary participation /No/Yes |
| Evans MDR, Kelley J, Zanjani ED (2005)  [45] | Yes and No (Previously done in IsssA surveys) | Yes    A number of questions was asked to avoid random measurement (Construct validity: Hypothesis-testing through correlation and  Factorial validity    Content validity: Language and scientific content accurate to measure opinions through social researchers    Face validity with target population | Yes    Correlation to assess reliability over time: test-retest (Pearson coefficient over 3 years)    Inter-item reliability (alpha) | N/A | Random sample drawn from electoral register    TDM sampling method: multi-step    Non-respondents were mailed over a period | Less social desirability bias by mail    Random measurement error minimized by variations on a topic    Balanced introduction to avoid favourable/unfavourable responses    Non-citizens omission shoudn’t have bias the results due to residence duration | IsssA surveys compared data with Census and found they match | No, info on decline participation/Pre-paid envelope/ No |
| Sturgis P, Cooper H, Fife-Schaw C (2005)  [49] | Yes  (BSAS and WT survey) | Yes  Content validity: wording and coding of attitude items was performed    Construct validity:  Hypothesis-testing (correlational analysis through regression model)  Construct of measurement for general scientific knowledge item: Scale formed by correct/incorrect responses to questions  Construct of items for attitude measurement (coding) | Yes    Internal consistency (Cronbach’s alpha for Knowledge scale evaluation by item) | BSAS: 62%  Self-completion questions: 86%    WT: 58% | BSAS:  Multi-stage stratified random sample    WT: Data drawn from baseline survey: Quota sampling | Not mentioned | BSAS:  Weighted sample of 976 respondents for self-completion questions: Reflect probability of selection of addresses, households and individuals    WT: Not mentioned | No/No/No |
| European Comission Directorate-General for Research.    (Eurobarometer 64.3)  (2005)  [41] | Yes and No (1996 survey) | Yes  Face validity: Question text and contingency text based on TNS Opinion & Social and assists for discrepancies (machine-readable documentation) found can be corrected through data provider documentation: Content validity    Construct validity:  - Hypothesis-testing  - Convergent validity  (Constructed variables for some index or status) | Yes  Split-ballot design reveals split-half reliability | Different by country | A multi-stage sampling design: 1st: Selection based on administrative regions by proportion of sample size for degree of urbanization.  2nd: a cluster of addresses was selected from 1st stage randomly  3rd: Individual random selection procedure | Not mentioned | National weighting based on Universe from EUROSTAT population data or from national statistic offices      International weighting done by TNS Opinion & Social based on EUROSTAT or national statistic offices | No/No/No |
| Sato H, Akabayashi A, Kai I (2006)  [87] | Yes | Not mentioned | Not mentioned | 34.5% | Random stratified sampling: Questionnaires sent for 15 cities and towns selected nationwide | Not mentioned | Not mentioned | No/No/No |
| Barnett J, Cooper H, Senior V (2007)  [85] | No (Previous BSA surveys) | Yes  Face validity for BPE only even though limited  Construct validity: Hypothesis-testing (Variables to relate with gene therapy allowing – Regression coefficient) | Yes  Regression coeficients n-standardized  Alpha scoring for items measuring public attitudes and other features (internal consistency) | 59% | Confuse/Inconclusive:  Sampling from a quota sampling (From BSAS) | Face validity could have been limited due to not realistic public involvement | Data weighted for differences in the probability of individual and household selection    Unweighted data on tables | No/No/No |
| European Comission Directorate-General for Research  (Eurobarometer 73.1) (2010)  [42] | Yes (New when compared to previous surveys) | Yes  Face validity: Question text and contingency text based on TNS Opinion & Social and assists for discrepancies (machine-readable documentation) found can be corrected through data provider documentation: Content validity    Construct validity:  - Hypothesis-testing  - Convergent validity  (Constructed variables for some index or status) | Yes    Split-half ballot reveals split-half reliability | Different by country | A multi-stage sampling design: 1st: Selection based on administrative regions by proportion of sample size for degree of urbanization.  2nd: a cluster of addresses was selected from 1st stage randomly  3rd: Individual random selection procedure | Not mentioned | National weighting based on Universe from EUROSTAT population data or from national statistic offices    International weighting done by TNS Opinion & Social based on EUROSTAT or national statistic offices | No/No/No |
| **Authors** | **Item generation** | **Validity** | **Reliability** | **Response rate** | **Sampling** | **Bias** | **Weighing** | **Informed Consent/Incentives/Ethical Statement** |
| European Comission Directorate-General Science, Research and Development XII  (Eurobarometer 46.1) (1996)  [39] | Yes and No (1991 and 1993 surveys) | Yes  Face validity: INRA (Europe) carried fieldwork coordinated by ECO  Question text and contingency text based on INRA codebook and assists for discrepancies (machine-readable documentation) found can be corrected through a Central Archive: Content validity  Construct validity:  - Hypothesis-testing  - Convergent validity  Constructed variables for some index or status | Yes  Split-half samples reveals split-half reliability (internal consistency) | 15900 of 16246 | Multi-stage sampling: Random address selection and individual selection of respondents:  Degree of urbanization for sampling frames | Not mentioned | Results weighed according to proportion of each nation based on demographics | No/No/No, but Disclaimer |
| Macer DRJ (1997)  [54] | No (Based on Macer 1994; OTA, 1986; Macer, 1992) | Yes  Content validity: Questions were translated and pre-tested | Not mentioned | 6000 questionnaires returned during 1993 (N/A) | Clustered random sampling method: Representative cross-section of community on maps  Questionnaires randomly distributed nationwide | Not mentioned | Not mentioned | No/No/No |
| Macer D, Bezar H, Harman N, Kamada H, Macer N (1997)  [63] | Yes and No (Eurobarometer 1996 and Einsiedal, Canada 1997) | Yes  Face and content validity: Eurobarometer 1996 (same questionnaire with small modifications); Einsiedal and members of BEPCAG (design of the survey) | Not mentioned | 22% NZ    44% Japan | Random telephone surveys    Margin of error 4-5% NZ and Japan (95% CI for 50% answer) | Not mentioned | Weighed average for some answers (1996 Eurobarometer) | No/No/No, but correction and admission of fraud by one interviewers |
| Norton J, Lawrence G, Wood G (1998)  [52] | Yes and No | Yes  Construct validity:  4-sections of survey designed based on Beus and Dunlap, 1991; Decima Research 1993 from Canada and Bureau of Industry Economics, 1995 | Not mentioned | 45% (responded)52% (responded + non-responded) | Randomly seleted from Electoral Roll | Not mentioned | Not mentioned | No/No/No |
| Hampel J, Pfenning U, Peters HP (2000)  [51] | Inconclusive | Yes  Construct validity:  Correlation coefficient between individual applications of genetic engineering; Linear and multiple regression regarding attitudes to genetic engineering and overall assessment; Attitudes dependent on social variables | Not mentioned | 56.3% | Selection of population through stratification (state/district) - 1st phase  Random selection of telephone numbers – 2nd phase  Target person – 3rs phase | Not mentioned | Not mentioned | No/No/No |
| Macer DRJ, Ng MAC (2000)  [55] | No (1991, 1993, 1997 surveys) | Not mentioned | Not mentioned | 12% | Random mail survey | Not mentioned | Not mentioned | No/No/No |
| Magnusson MK, Hursti UKK  (2002)  [53] | Yes and No (Health Attitude Scales by Roninnen et al, 1999; Knowledge questions from Eurobarometer 1996) | Yes  Face validity with target population (41 subjects in Uppsala)  Content validity: Previous questionnaires performed by same authors (different subject)  Factorial analysis by 20-item Health Scale Attitude (Factorial validity);  Construct validity: Employed in questionnaires following interviews with small sample (Halved);  Hypothesis-testing: relationship between GM foods attitudes with the consumer’s interests | Yes  Internal consistency or homogeneity: Cronbach’s alpha for Health Scale Attitude (NPI and GHI) | 39% | Random selection from national population register | Recruitment/Selection bias:  Over-representation of population with higher education | Difference between respondents and non-respondents of original questionnaire | No/Yes (Lottery ticket or Donation to charity)/No |
| Hallman WK, Adelaja AO, Schilling BJ, Lang JT (2002)  [60] | Yes and No (Some features coming from Eurobarometer 1999) | Yes  Face and content validity: Representatives from different environments consulted to generate questions; Wording, order of questions and terminology´  Construct validity:  - hypothesis-testing  - Self-rating vs quiz correlation (Convergent validity) | Yes  Split-half sample  (Split-half reliability) | N/A | Random probability sample drawn from 97 million telephone households in US  (recent birthday)  Quotas to ensure representativeness  Sampling error rate: ±3% (95% CI)    Commensurate with state populations (US Census Bureau) | Not mentioned | Compensation for under-represented groups: Comparison data from 2000 Census and adjusted for demographics    Statistical analysis are done with unweighted data | No/No/No |
| European Comission Directorate General for Research  Eurobarometer 58.0 (2002)  [40] | Yes and No (1996 survey) | Yes  Content validity: Survey design and analysis conducted by a research group (European team devised and set the questions)  Factorial  Construct validity  Convergent validity (Knowledge index) | Yes  Split-ballot design: half genetic engineering, half biotechnology  Internal consistency:  The three items form a reliable scale (Cronbach’s alpha = 0.67) and provide an index of awareness of biotechnologies for each respondent, with a range from 0 to 3.  Knowledge as a possible attribute of the engaged public of biotechnology measure by a reliable scale (Cronbach's alpha  = 0.74). Hence the total number of correct answers is used as an index of knowledge of  biology and genetics for each respondent. | Different by country | A multi-stage sampling design: 1st: Selection based on administrative regions by proportion of sample size for degree of urbanization.  2nd: a cluster of addresses was selected from 1st stage randomly  3rd: Individual random selection procedure | Not mentioned | Nation Weights incorporate post-stratification adjustments based on cross tabulations of national populations by sex, age, region (NUTS II), and size of locality. Some countries have additional variables | No/No/No |
| Inaba M, Macer DRJ (2003)  [56] | No (derived from 1991, 1993, 1997, 2000 surveys) | Yes  Face and content validity: Eurobarometer 1996 (same questionnaire with small modifications); Einsiedal and members of BEPCAG (design of the survey) | Not mentioned | 20% | Random selection across Japan personally delivered questionnaires (47 prefectures)    Sample error: ±5% | Not mentioned | Not mentioned | No/No/No |
| Inaba M, Macer DRJ (2003b)  [64] | No (Previous surveys from Macer et al, 1997 and modified Eurobarometer 1996 (Einsiedal, 1997 and Gaskell et al, 2000) | Yes  Face and content validity: Eurobarometer 1996 (same questionnaire with small modifications); Einsiedal and members of BEPCAG (design of the survey) | Not mentioned | 20% Japan | Random telephone surveys | Not mentioned | Weighed average for some answers (1996 Eurobarometer) | No/No/No |
| Hallman WK, Hebden WC, Aquino HL, Cuite CL, Lang, JT (2003)  [61] | Yes and No (Eurobarometer 2002) + 2001 survey from Hallman et al, 2002 and Canada (Einsiedel, 2003) | Yes  Face and content validity: Food Policy Institute researchers designed, commented questionnaire and multi-institutional representatives gave advice and input; Question wording and order  Construct validity  - hypothesis-testing  - Self-rating vs quiz correlation (Convergent validity) | Yes  Split-half questions  (Split-half reliability) | 38% of phone selected    56% of working residential phone    65% available and eligible | Random but balanced selection process to ensure representativeness    Distribution proportionate to demographics (US Census Bureau)    Sampling error: ±3%    Split ballot sampling error: ±4% | Not mentioned | Weight adjustments achieved by demographics    Statistical analysis are done with unweighted data | No/No/No |
| Puduri V, Govindasamy R, Lang JT, Onyango B (2004)  [62] | No (From previous Food Policy Institute surveys) | Yes  Construct validity: Influence of socio-economic and value attributes variables on GM animal approval: Hypothesis-testing | Not mentioned | Not mentioned | Sampling error: ±4% | Not mentioned | Not mentioned | No/No/No |
| Small BH, Parminter TG, Fisher MW (2005)  [57] | Yes | Yes  Face validity given by design of the items and adequate content validity to be used in the constructs of choice;  Content validity: Content domain of particular contrusct  Construct validity:  Psychometric robust (sub)scales to measure constructs relative to genetic engineering and relationship with “intention to purchase” hypothetical GE products: hypothesis-testing (subscales)  - External validity | Yes  Scales generated to be used in future surveys as measure of internal consistency (Cronbach’s alpha and factor analysis) | 56% valid | Random sample (Stratified by region and income) | Low representation of Maori    Non-response bias: not represented    Encouraging of risk takers by money prize participation    Response bias: Social desirability is possible | Not mentioned | No/Yes (Money Prizes)/No |
| Nayga RM, Fishera MG, Onyango B (2006)  [58] | Yes and No (Many questions translated from Hallman et al, 2003) | Yes  Face and content validity following the survey on Hallman et al, 2003 with modifications for cultural differences  Construct validity given by hypothesis-testing of factors that influence GM technology approval for animal-based products | Not mentioned | US:  38% of phone selected  56% of working residential phone  65% available and eligible    SK:  40% cooperation rate | Random proportional probability dialing (US)  Random but balanced selection process to enxure representativeness (US Census Bureau)  Sampling error: ±3%  Split ballot sampling error: ±4%    SK:  Random sampling proportional to population by region  Sampling error: ±3% (desired)  Non-sampling error minimized by orientation | Age bias: Sampling procedure skews age proportions by comparison to Census figures (SK) | Weight adjustments achieved by demographics (US) | No/ Yes (a pen = 2$ US dollar /No |
| Govindasamy R, Onyango B, Hallman WK, Jang H-M, Puduri V (2008)  [59] | Yes and No (Many questions translated from Hallman et al, 2003) | Yes  Face and content validity following the survey on Hallman et al, 2003 with modifications for cultural differences  Construct validity given by hypothesis-testing of factors that influence GM technology approval for animal-based products | Not mentioned | Cooperation rate: 40% | Random draw from 7 large and 9 small cities  Household size determined stratified sample selection    Sampling error: ±3.1% (95% CI)    Non-sampling error minimized by orientation | Not mentioned | Weighting of data by demographics variables similar to US except race/ethnicity whose came from South Korean National Census | No/Yes (a pen = 2$ US dollar)/No |
| **Authors** | **Item generation** | **Validity** | **Reliability** | **Response rate** | **Sampling** | **Bias** | **Weighing** | **Informed Consent/Incentives/Ethical Statement** |
| Chikhazhe TL (2015)  [67] | No (Based on Small City surveys done in previous years – questions from 2009 survey and some ne included) | Yes  Content validity (Small City surveys done in previous years by agroup of panelists)  Construct validity:  Demographic variables influence on attitudes to GM: Hypothesis-testing | Not mentioned | 353 from 850 | Random from online database (Quota groups balanced) | Not mentioned | Not mentioned | Yes/Yes (Entries for competition prizes; real-time reward)/No |
| McCaughey T, Sanfilippo PG, Gooden GEC, Budden DM, Fan L, Fenwick E, et al (2016)  [72] | Yes | Yes  Face validity: cognitive phase testing to refine questionnaire with 10 participants with attention to interpretation and understading (Content validity) – Readability through Gunning Fog Index  Construct validity: First part of the questionnaire with demographic details hypothesized to have influence on attitudes (Multinomial logistic regression - covariates) | Not mentioned | N/A | Voluntary due to online survey and social media advertisement | Recruitment and selections biases (mitigated by diverse backgrounds campaigning)  Translation bias | Not mentioned | No/No/Yes |
| STAT and Harvard T.H. Chan School of Public Health (2016)  [74] | Yes | Yes  Content validity: Close work between representatives from STAT and Harvard T.H Chan School of Public Health developed and analyzed questionnaire  Construct validity:  Representatives of 2 different organizations developed the survey | Yes  Split-half sample questions (split-half reliability) | Not mentioned | Random selection of adults  Sampling error for total and combined sample (±3-3.7) | Non-response bias due to telephone survey (Non-sampling error) | Sample weighed by household size, cell phone/landline and demographics | No/No/No |
| Funk C, Kennedy B, Sciupac E  Pew Research Center (2016)  [76] | Yes | Yes  Face validity: Expert advisers from outside were recruited to help in questionnaire design as well as 6 focus groups outcome  Content validity: Pew Research Center team designed, questionnaire and pilot-tested it to test question wording and information on the survey | Not mentioned | March wave:  68% web  68% mail  April wave:  83% web  77% mail  3% cumulative response rate | Random selection  Sampling error ±2.2% pp (95% CI) | Error bias due to question wording and practical difficulties on conducting surveys | Base weight incorporating the respondents’ original survey selection probability and subsampling for invitation to the panel and finally, demographics. Population density also weighed | No/No/No |
| Cormick C, Mercer R (2017)  [71] | Yes and No (Survey methodology replicated plus additional questions based on new data from literature | Yes  Face validity: Meeting with OGTR to define oucomes and reviewing existing body of literature.  Content validity: pilot testing of survey.  Construct validity: 95% level of confidence for treatment of means using scales | Not mentioned |  | Online and booster CATI (phone) – Quota set based on states and territories type and gender – Representative national sample | Survey methodology replicated of previous years to avoid impact of externalities  Care to manage tendency of respondents to favour “risk” responses  No gender bias: 50/50  Similar areas were drawn for recruitment of individuals | Unweighted sample of survey  Weighting and statistical analysis of survey results made based on unweighted state and were based on State/Territory, age and gender | No/No/No |
| Chen, C, Liang, Z (2017)  [82] | Yes | Not mentioned | Not mentioned | N/A | Not mentioned | Not mentioned | Not mentioned | No/No/No |
| Gaskell G, Bard I, Allansdottir A, da Cunha RV, Eduard P, Hampel J, et al (2017)  [78] | Yes | Yes  Content validity: “Assiduous attention” given to comparable wording and phrase meaning  Construct validity:  Multiple regression coefficients obtained by country were assessed for agreement between variables (And R square) - Hypothesis-testing | Not mentioned | 25% quality interviews | Quota samples (online surveys) | Not mentioned | Not mentioned | No/No/Yes |
| Scheufele DA, Xenos MA, Howell EL, Rose KM, Brossard D, Hardy BW (2017)  [75] | No (National survey data – YouGov) | Yes  Content validity: Wording designed to differentiate items  Construct validity:  Hypothesis-testing (Independent and dependent variables)  Multivariate models using regression  Correlation index dependent variables influencing attitudes (mediating or confounding items) | Yes  Inter-item reliability given by Chronbach’s alpha index (factual knowledge)  Composite of items (acceptability) | 41.7% | Quota sampling:  demographics and political interests | Not mentioned | Not mentioned | No/No/No |
| Weisberg SM, Badgio D, Chatterjee A (2017)  [83] | Yes | Yes  Face and Content validity: Wording of vignettes and participant gives opinion only for 1 version of the vignettes  Construct validity (Hypothesis-testing):  - Testing of two hypotheses (demographic variables and presentation of technology)  - Effect of risk in split half samples  - Effect of metaphors | Yes  Split-half samples for study 2: Risk-before and Risk-after (split-half reliability) | Almost 100% (only 1 ID discarde) | Quota sampling:  Participants selected based on US and that completed >500 studies at M-Turk with approval rate >95% given by study administrators  (Convenient sample) | People who fills surveys are more open to promises of new technology: Response bias  Young people are left leaning and may have biased sample  Over-representation of groups based on education and race: selection bias  Sample is probably more robust than if sampling had been made by telephone  M-turk population is diverse and covers wide range of education and income levels (Avoiding bias)  Metaphors also bias people’s attitudes | Not mentioned | Yes/Yes (0,25$/person)/Yes |
| Wang J-H, Wang R, Lee JH, Iao TWU, Hu X, Wang Y-M, et al (2017)  [81] | Yes (Some questions are similar to survey used by Macer and colleagues which served as possible inspiration | Yes  Content validity:  Questions formulated and tested with 10 participants to refine (Face validity). Questions refined for understanding for use in survey  Construct validity: Likert scale were built to study association between independent variables: Hypothesis-testing | Not mentioned | 97.3% (83.6% public) | Voluntary sampling  Proportional distribution of people across China | Young age bias mitigated by other online tools spread by personal contacts, friends, etc  Wording of “gene therapy” phrase might lead to undesirable response  Responses influenced by medical backgrounds  Recruitment bias and possible non-representativeness of the sample (84.4% of public had a bachelor degree): Selection bias | Not mentioned | Yes/No/Yes |
| Hendriks S, Giesbertz NAA, Bredenoord AL, Repping S (2018)  [79] | Yes and No (Macer et al, 1995) | Content validity: Questionnaire reviewed by an expert panel (Face validity) of different professions for scientific accuracy and non-directive phrasing; understandable language for lay public.  Construct validity:  - Several respondent characteristics were used for ordinal regression analysis to correlate with willingness to use genetic modification | Not mentioned | N/A | Voluntary sampling | Watching documentary previous to survey could have had an effect on attitudes  Recruitment bias: Dissemination through social media | Not mentioned | No/No/No |
| Uchiyama M, Nagai A, Muto K (2018)  [86] | Yes | Not mentioned | Not mentioned | 24.5% | Quota sampling | Potential recruitment bias: Conducted online | Not mentioned | No/No/No |
| Lakomý M, Bohlin G, Hlavová R, Macháčková H, Bergman M, Lindholm M  ORION (2018)  [80] | Yes and No (Parts from other surveys  namely Eurobarometers and some national surveys) | Face validity: Pilot tested with target population from all the countries involved  Content validity: Preparation of the questionnaire in local language translated *a posteriori* revised by expert panel (native and non-native speakers)  Construct validity: The items were developed in multiple stages by gathering input from all institutions participating in ORION project | Not mentioned | 90% response rate in total | Random sampling | Not mentioned | Proper ratio of landlines and mobile phone and margined quota on demographics | No, but participants could leave at any time and skip questions/No/No |
| Funk C and Heferon M  Pew Research Center (2018)  [77] | Yes | Statistical/Empirical validation:  Exploratory Factor Analysis (EFA) and Item-Response Theory (IRT) | Yes  Internal consistency: Cronbach’s alpha and Pearson correlation coefficients for Science knowledge index scale | 82%  2.3% cumulative response rate | Random selection by telephone (RDD)  Sampling error ±2.8% pp (95% CI) | Error bias due to question wording and practical difficulties on conducting surveys | Base weight incorporating the respondents’ original survey selection probability and subsampling for invitation to the panel and finally, demographics. Population density also weighed  No internet access weighed for respondents without internet access | No/No/No |
| Funk C and Heferon M  Pew Research Center (2018b)  [65] | Yes | Statistical/Empirical validation:  Exploratory Factor Analysis (EFA) and Item-Response Theory (IRT) | Yes  Internal consistency: Cronbach’s alpha and Pearson correlation coefficients for Science knowledge index scale | 82%  2.3% cumulative response rate | Random selection by telephone (RDD)  Sampling error ±2.8% pp (95% CI) | Error bias due to question wording and practical difficulties on conducting surveys | Base weight incorporating the respondents’ original survey selection probability and subsampling for invitation to the panel and finally, demographics. Population density also weighed  No internet access weighed for respondents without internet access | No/No/No |
| McCaughey T, Budden DM, Sanfilippo PG, Gooden GEC, Fan L, Fenwick E, et al (2019)  [73] | Yes and No (McCaughey et al, 2016) | Yes  Face validity: cognitive phase testing to refine questionnaire with 10 participants with attention to interpretation and understading (Content validity) – Readibility through Gunning Fog Index | Not mentioned | N/A | Voluntary due to online survey and social media advertisement | Recruitment and selections biases (mitigated by diverse backgrounds campaigning)  Gender bias towards male respondents | Not mentioned | No/No/Yes |
| Critchley C, Nicol D, Bruce G, Walshe J, Treleaven T, Tuch B (2019)  [70] | Yes | Yes  Construct validity:  - Convergent validity (2 or 4 items to assess same attitude)  - Hypothesis-testing: Predicting attitudes through a multilevel model approach for each application, cell type and demographic variables influence and individual difference factors | Not mentioned | 6-9% with cooperation rate 13.8-15.4% (CATI) | Random sampling: telephone  Quota sampling: Online panel stratified by gender | Positivity bias due to the less negative choice (germ cells vs embryo) – Response bias  Contrast bias due to susceptibility to cognitive bias caused by a single factor | Not mentioned | No, but possibility of privacy statement/No/Yes |
| McConnachie E, Hotzel MJ, Robbins JA, Shriver A, Weary DM, von Keyserlingk MAG (2019)  [68] | Yes and No (Knowledge from Hallman et al, 2004) | Yes  Content validity: Conceptualization of the questionnaire  Construct validity:  - Correlations between ordinal and continuous scales; Comparison of Likert scales  - Hypothesis-testing driven by attitudes relationship with demographic and social variables | Not mentioned | 434 from 598 initially recruited  Survey completion time: 4.9 min±2.3 | Convenient sampling and not representative 🡪 M-turk | Self-selection bias was avoided by not mentioning survey nature  Social desirability bias was minimal due to question on “Most Americans” consume  Prior information might have had a biased attitude to GM animals  Authors work all in the same subject which might have influenced manuscript tone and research direction | Not mentioned | Yes/Yes (0.6$)/Yes |
| Yunes MC, Teixeira DL, von Keyserlingk MAG, Hotzel MJ (2019)  [69] | Yes and No (Knowledge based on previous surveys) | Yes  Content validity: Conceptualization of the study by several authors  Validation by 2 authors (Face validity)  Survey reviewed and refined after first 30 participants (Face validity)  Construct validity: Hypothesis-testing with multinomial logistic regression (Spearman) using predictor and dependent variable (Atittude)  Reclassification of Likert scale | Not mentioned | 570 from initial 677 recruited | Voluntary and not representative (only 3 southern states) | Self-selection bias reduced by questioning without revealing survey nature  Selection bias: over-representation of demographics and non-representative of Brazilian population (university education) | Not mentioned | Yes/No/Yes |
| Kohl PA, Brossard D, Scheufele DA, Xenos MA (2019)  [84] | No (Question item from previous survey on gene editing applications) | Yes  Construct validity:  - Convergent validity (Measure in averaged 2 items – Risk, Benefit, Moral, beliefs)  - Hypothesis-testing: individual level factors would predict views | Yes  Internal consistency item measures  Chronbach’s alpha (> 2 items) and Pearson’s (2 items) | 41.7% | Quota sampling: Respondents drawn from a panel based on demographics  Representativeness ensured | Familiarity with other genetic engineering applications 🡪 spill over into evaluation of less familiar applications  No examples shown | Respondents drawn from YouGov platform were weighed to the sampling frame based on propensity scores from Census Bureau’s 2010 | No/Yes (paid envelopes)Yes (study approval from Wisconsin Univ) |
| Lull RB, Akin H, Hallman WK, Brossard D, Jamieson KH (2019)  [66] | Yes and No (Adapted from other studies) | Yes  Construct:  Convergent validity: Observed and latent variables and Discriminant validity: correlation between all latent factors and AVE (Fornell-Larcker criterion)  Hypothesis-testing: Concepts or measures as likely influencing factors were evaluated towards attitudes to GE mosquitoes  Previously validated survey questions  Criterion validity: Concurrent - Model tested against alternative models | Yes  Composite reliabilities indicates internal consistency | Not mentioned | Random digit dialing (independent company) | Not mentioned | Not mentioned | No/No/No |

Highlighted in yellow: Doubts and confirmation to be done
