# Supplementary material for: What do people think about genetic engineering? A systematic review of questionnaire surveys before and after the introduction of CRISPR
Source: Front Genome Ed. 2023 Dec 19;5:1284547. doi: 10.3389/fgeed.2023.1284547 (PMC10773783; doi:10.3389/fgeed.2023.1284547)
Supplement: Supplementary file 8 [file Table4.DOCX]

| **Authors (years)** | **Country** | | **Topic** | **Scale** | **Questions** | **Answers** |
| --- | --- | --- | --- | --- | --- | --- |
| OTA (1987)  [34] | US | | **Awareness, knowledge and atittudes** towards genetic engineering in:  - **humans** (somatic and germline)  - **human cells**,  - **farm animals**  - **animal cells**  **- cross-breeding animals**  **Awareness and attitudes towards**  Human gene therapy and enhancement in humans (germline and somatic) | (IF ‘VERY GROUP 1.  very good, adequate or poor?  4-point scale  • A lot, fair amount, Relatively little, Almost nothing (4);  Dichotomous   - Better/Worse - Yes/No, Not Sure - Morally wrong/Not morally wrong, Not sure;   6-point scale   - A lot better, Somewhat better, Somewhat worse, Much worse, Not sure, No effect (6); - Very likely, Somewhat likely, Somewhat unlikely, Very unlikely, Not sure (6);   10 point-Likert Scale   - From Totally unacceptable (1) to Totally acceptable (10);   5-point scale   - Strongly approve, Somewhat approve, Somewhat disapprove, Strongly disapprove, Not sure (5); - From Very unwilling to Very willing (5) - Both, Only affecting offspring, Offspring, Neither, Not sure (5); - Qualitative | - *If you had to rate your own basic understanding of science and technology*, would you say it is …  - *How much have you heard or read about genetic engineering*?  *- What is meant by genetic engineering?”*  - *where would you rank genetic manipulation of …?*  *… human cells*  *… animal cells*  *… bacteria cells*  *… plant cells*  - *On balance, do you feel that changing the genetic makeup of human cells is morally wrong, or not?*  - *How do you feel about scientists changing the makeup of human cells to…*  *... stop children from inheriting fatal genetic disease*  *… cure fatal genetic diseases*  *… stop children from inheriting nonfatal birth defects*  *… reduce the risk of developing fatal disease later in life*  *… inherited intelligence in children*  *… improve physical characteristics in children*  How willing would you undergo genetic therapy if…  *… you were Iikely to get a serious or fatal genetic disease later in life,*  … *you had a child with a usually fatal genetic disease,*  - *Suppose someone had a genetic defect that would cause usually fatal diseases in them and would likely be inherited by their children. Do you think that doctors should be allowed to correct only the gene affecting the disease in the patient, only the gene that would carry the disease to future generations, both genes, or neither gene?*  Q15a.*Have you heard* about biological techniques, such as cross-fertilizing plants or *cross-breeding_ animals to produce hybrids?*  Q15b*.Do you believe that* creating hybrid plants and *animals by cross-breeding* is morally wrong, or not?  Q15C. If the new plant or *animal produced by cross-breedinq can reproduce itself*, *how likely do you think this is to pose a danger to the environment* -- very likely, somewhat likely, somewhat unlikely or very unlikely?  - *If there was no direct risk to humans, how much would you approve…?*  *… New treatments for cancer*  *… New vaccines*  *… Resistant crops*  *… Frost resistant crops*  *… More productive farm animals*  *… Larger game fish*  - *Have you heard about any potential dangers from genetically engineered products?*  - *What potential dangers have you heard of?*  *- How likely do you think it is that genetically engineered products will represent a serious danger to people or the environment?*  Q22. From what you have heard and read, how likely *do you think it is that the use of genetically engineered organisms in the environment will represent a …?*  *… Difficulty in controlling spread*  *… Health hazards and side effects*  *… Mutations*  *… Environmental contamination*  *… Unforeseen consequences*  *… New diseases*  *… Cancer*  *… Antibiotic resistance diseases*  *… Side effects*  *… Dangers to people and animals who consume it* | \| **Awareness** \| *Almost nothing* \| *Relatively little* \| *A fair amount* \| *A lot* \| \| --- \| --- \| --- \| --- \| --- \| \| *Genetic engineering* \| 24 \| 39 \| 35 \| 6 \|   - The majority of US respondents (39%) heard or read relatively little and similar percentage (35%) a fair amount about genetic engineering  - From the ones that heard/read a fair amount about genetic engineering, 17% have a poor understanding of Science   \| **Genetic engineering meaning** \| *How many (%)* \| \| --- \| --- \| \| *Altering/Manipulating genes* \| 20 \| \| *Producing improved/superior organisms* \| 7 \| \| *Crossbreeding and producing hybrids* \| 6 \| \| *Producing cures for genetic diseases or defects* \| 6 \| \| *Producing desired or particular characteristics* \| 5 \| \| *Producing new organisms or forms of life* \| 4 \| \| *Producing a super race or perfect people* \| 4 \|  \| **Acceptability (0-6)** \| *Mean* \| \| --- \| --- \| \| *Human cells* \| 4.5 \| \| *Animal cells* \| 5.3 \| \| *Bacteria cells* \| 5.6 \| \| *Plant cells* \| 6.6 \|   - 56% can provide meaningful explanation but not explanation genetic engineering  - Plant cells manipulation is the most acceptable by US public whereas human cells are the least   \| **Morality** \| *Not wrong* \| *Wrong* \| *Not sure* \| \| --- \| --- \| --- \| --- \| \| *Changing human cells makeup* \| 52 \| 42 \| 6 \|   - The majority say it’s not morally wrong to change makeup of human cells  - Education increases support for genetic manipulation of human cells  - The higher the awareness about genetic engineering, the higher the support for changing human cells makeup   \| **Changing human cells makeup to…** \| *Approve* \| *Disapprove* \| \| --- \| --- \| --- \| \| *… stop children from inheriting fatal genetic disease* \| 84 \| 15 \| \| *… cure fatal genetic diseases* \| 83 \| 14 \| \| *… stop children from inheriting nonfatal birth defects* \| 77 \|  \| \| *… reduce the risk of developing fatal disease later in life* \| 77 \|  \| \| *… inherited intelligence in children* \| 44 \| 53 \| \| *… improve physical characteristics in children* \| 44 \| 54 \|   - Big majority of people approve changing human cells genetically in for health reasons despite being diseases being or not fatal  - Approval is given by majorities that think it is wrong to do it as well as from majorities that think is not morally wrong  - If the purpose is to save children, it is approved by majority of the ones who think is morally wrong  - Therapeutic reasons are much more approved than eugenic reasons despite the latter having high approval and preoccupation about morality and utility of it   \| **Undergo genetic therapy to…** \| *Willing* \| \| --- \| --- \| \| *… correct a genetic proclivity to fatal disease* \| 78 \| \| *… a child in need if it had a fatal disease* \| 86 \|   - Almost all US respondents approve genetic therapy to save lives   \| **In case of fatal disease, doctors should be allowed to alter…** \| *How many agree (%)* \| \| --- \| --- \| \| *… gene of disease and not gene carrying disease to future generation* \| 8 \| \| *… both genes* \| 62 \| \| *… no gene* \| 11 \|   - Distinction between somatic and germline seems to not exist  - People think doctors should be more restricted to gene therapy than germline applications   \| **Genetic engineering to produce…** \| *Approve* \| \| --- \| --- \| \| *… New treatments for cancer* \| 96 \| \| *… New vaccines* \| 91 \| \| *… Resistant crops* \| 87 \| \| *… Frost resistant crops* \| 87 \| \| *… More productive farm animals* \| 74 \| \| *… Larger game fish* \| 66 \|   - A clear majority of US respondents approve all genetic engineering applications  - Rate of social utility-crop survival increases approval compared to recreational one   \| **Potential dangers heard of genetically engineered products** \| *How many? (%)* \| \| --- \| --- \| \| *Difficulty in controlling spread* \| 16 \| \| *Health hazards and side effects* \| 12 \| \| *Mutations* \| 10 \| \| *Environmental contamination* \| 7 \| \| *Unforeseen consequences* \| 7 \| \| *New diseases* \| 6 \| \| *Cancer* \| 6 \| \| *Antibiotic resistance diseases* \| 3 \| \| *Side effects* \| 3 \| \| *Dangers to people and animals who consume it* \| 3 \|   - Controlling spread of GE products, health hazards and mutations are the most potential dangers heard by US people  - 61% say they think a serious danger from GE products is likely from the ones that think the current growth rate of such products is being too fast  - 57% say production of birth defects in humans is mostly seen as somewhat likely by US citizens when using GMOs in the environment  - Evoked by a minority of the public but still seen as somewhat likely as well are GMOs that will mutate into deadly disease (46%) and lead to animal extinction (45%)  - Very likely that birth defects may happen as a consequence of GMOs release in the environment and animal extinction are 18% and 11%, respectively |
| Comission of the European Communities Directorate-General Science, Research and Development  (Eurobarometer 35.1) (1991)  [37] | EC12 | | **Objective and subjective knowledge** of biotechnology/genetic engineering  **Attitudes** to genetic engineering for:   - Human beings - Farm animals - Animals to develop life-saving drugs and/or study human diseases | Dichotomous 🡪 Numerical scale from 0-7  • Yes/No + D/K🡪 0-7;  5-point scale 🡪 Numerical scales: From +2 to -2 (means)  • From Strongly agree to  Strongly disagree 🡪 Means between +2 and -2  12-point scale   - From Very simple (1) to Very complicated (12) | - In your opinion, *which of these are linked to biotechnology & genetic engineering and which are not*  *…Research on early detection and treatment of cancer*  *… Changing hereditary characteristics within an organism to alter that organism’s characteristics*  *… Producing new kinds of organisms using hereditary information from other species*  *… Improving traditional methods of cross-breeding plants or animals*  *… Making use of living microorganisms, for example for plant protection (bio-pesticides)*  *… Food processing such as using yeast for the production of bread or beer*  *… Treating hereditary human diseases by modifying the tissue involved*  - How *did you find the topics we have talked about over the last few minutes: rather simple or rather complicated?*  - To what extent you agree or disagree with each of the following statements concerning …  … genetic *engineering to farm animals, to change them in quicker or more precise ways than traditional breeding programmes, in order to make them more useful: for example, make them resistant to diseases, or grow faster, or produce more or better quality meat or milk.*  *… genetic engineering to animals to develop life-saving drugs, or to study human diseases. Animal protection is guaranteed by law and some people say it is morally wrong to apply biotechnology/genetic engineering to animals.*  *…* genetic engineering to human beings, or *to their cells and tissues, for various purposes such as detecting, or curing diseases, and characteristics we might have inherited from our parents and research on human beings*, medicines and vaccines. | \| **Objective knowledge (0-7)** \| *Mean* \| \| --- \| --- \| \| *Biotechnology* \| 4.16 \| \| *Genetic engineering* \| 4.02 \| \| *Subjective knowledge (0-10)* \| 5.19 \|   - Not a very significant difference between these terms  - A statistically significant divergence is however masked due to what is favored by people in each of these  - The highest level of knowledge is only achieved by 24% in biotechnology sub-sample and 19% in genetic engineering one  - Educational level increases knowledge of biotechnology and genetic engineering  - The higher the objective knowledge the higher the optimism  - Subjective knowledge is below the median of the scale (5.19)   \| **To what extent do you agree with research on…** \| *Agree* \| *Disagree* \| *Mean* \| \| --- \| --- \| --- \| --- \| \| *Plants* \| 74 \| 18 \| 0.98 \| \| *Farm animals* \| 42 \| 49 \| -0.1 \| \| *Food to improve food and drink quality* \| 58 \| 32 \| 0.47 \| \| *Microorganisms to produce food products* \| 78 \| 12 \| 1.17 \| \| *Microorganisms for break down waste products* \| 87 \| 5 \| 1.57 \| \| *Animals to develop medicines and vaccines* \| 88 \| 4 \| 1.59 \| \| *Human beings to detect and cure diseases* \| 74 \| 16 \| 1.04 \|   - All applications are perceived as positive, worthwhile and to be encouraged except farm animals research  - The higher the objective knowledge, the higher the support  - The lowest the support, the highest the risk for every application |
| Macer (1992)  [35] | Japan | | **Perceptions** of genetic manipulation of human cells (**Awareness and knowledge**)  **Attitudes** to human gene therapy | Dichotomous,   - Acceptable/Unacceptable - Benefit/No benefit (Risk)   Trichotomous   - Not heard - Heard words - Heard and understand - Qualitative   5-point scale  • From very willing to very unwilling + D/K (5) | - *How much have you heard or read about manipulating genetic material in human cells?* - *Is genetic manipulation of human cells acceptable to you?* - *Could genetic manipulation of human cells provide benefits for Japan (NZ)?* - *Could genetic manipulation of human cells present serious risks or hazards in Japan (NZ)?* - *Which reasons lead you to think that genetic manipulation of human cells is…*   *… unacceptable*  *… benefic*  *… risky*   - *If tests showed that you were likely to get a serious or fatal genetic disease later in life, how willing would you be to undergo therapy to have those genes corrected?* - *If you had a child with a usually fatal genetic disease, how willing would you be to have the child undergo therapy to have those genes corrected?* | \| **Genetic manipulation of human cells** \| *How many? (%)* \| \| \| --- \| --- \| --- \| \| *Japan* \| *NZ* \| \| *Not heard* \| 15 \| 35 \| \| *Aware* \| 49 \| 40 \| \| *Understand* \| 36 \| 25 \| \| *Acceptable* \| 26 \| 43 \| \| *Benefits* \| 38 \| 48 \| \| *Risks* \| 83 \| 74 \|   - People from Japan are more aware and understand better what genetic manipulation of human cells is  - Benefit and acceptability is higher in NZ as well as risk is perceived to be lower   \| **Top reasons for unacceptability of genetic manipulation** \| *Japan* \| *NZ* \| \| --- \| --- \| --- \| \| *Interfering with nature* \| 17.3 \| 28 \| \| *Playing God* \| 14.9 \| - \| \| *Fear of unknown* \| 8.4 \| 8 \|      \| **Top reasons for benefits of genetic manipulation** \| *Japan* \| *NZ* \| \| --- \| --- \| --- \| \| *Cure or prevent genetic disease* \| 22.8 \| 22 \| \| *Disease control* \| 14.8 \| 31 \| \| *Humanity and whole world benefits* \| 14.1 \| 22 \|  \| **Top reasons for risks of genetic manipulation** \| *Japan* \| *NZ* \| \| --- \| --- \| --- \| \| *Fear of unknown* \| 11.6 \| 13 \| \| *Danger of human misuse* \| 10.1 \| 11 \| \| *Playing God, unnatural* \| 8.7 \| 8 \|   - Japan and New Zealand citizens see interfering with nature as the main reason to not accept genetic manipulation  - Fear of unknown also feature as the top reason evoked by more than 10% of citizens in these countries as reason for risk of genetic manipulation  - In terms of benefits, cure or prevention of genetic diseases and control of diseases are the two main evoked by Japan and New Zealand citizens, respectively  - Major reasons for benefit concern human health and world benefits  - Similar reasons for unacceptability and risks of genetic manipulation, namely the unknown consequences and Playing God act   \| **Undergo genetic therapy to…** \| *Very willing* \| *Somewhat willing* \| *Somewhat unwilling* \| *Very unwilling* \| \| --- \| --- \| --- \| --- \| --- \| \| *… correct a likely genetic fatal disease* \| 25.2 \| 29.1 \| 18.0 \| 11.7 \| \| *… a child in need if it had a fatal disease* \| 36.9 \| 29.3 \| 11.2 \| 7.0 \|   - Greater acceptance in using gene therapy for children than for adults  - In general, respondents were more willing to use gene therapy in these situations  - There was ***no significant correlation for responses about gene therapy and level of awareness of genetic manipulation***, education, sex, age, etc |
| Comission of the European Community Directorate-General Science, Research and Development Unit XII/E/1  (Eurobarometer 39.1)  (1993)  [38] | EC12 | | **Objective and subjective knowledge** of biotechnology/genetic engineering  **Attitudes** to genetic engineering for:  - Human beings  - Farm animals  - Animals to develop life-saving drugs and/or study human diseases | Dichotomous 🡪 Numerical scale from 0-6 🡪  • True/False + D/K🡪 0-6;   - T/F + D/K 🡪 0-12   5-point scale Numerical scales: From +2 to -2 (means)  •From Definitely agree to  Definitely disagree 🡪 Means between +2 and -2  10-point scale   - From Very simple (1) to Very complicated (10) | - Here are some statements. For each of them, please tell me *whether you think it is true or false*.  1.There are bacteria which live from waste water  Most bacteria are harmful to human beings  2.The cloning of living things produces exactly identical offspring  3.Children look like their parents because they have the same red blood cells  4.It is possible to modify bacteria genetically so that they will produce useful substances  5.It is possible to find out whether a child will have Down's Syndrome (i.e. will be a "mongol"), within the first few months of pregnancy  6.Viruses can be contaminated by bacteria  7.Yeast for brewing beer consists of living organisms  8.It is possible to change the hereditary characteristics of plants, enabling them to develop their own defence against certain insects  9.Biotechnology/genetic engineering makes it possible to increase the milk production of cows  10.There are test tube babies who were developed entirely outside the mother's body  11. Genes of all living things on earth are made up of different combinations of only 4 or 5 chemical building blocks  - "How did you find the topics we have talked about over the last few minutes: *rather simple or rather complicated?*  *- To what extent you agree or disagree with each of the following statements concerning* …  … genetic *engineering to farm animals, to change them in quicker or more precise ways than traditional breeding programmes, in order to make them more useful: for example, make them resistant to diseases, or grow faster, or produce more or better quality meat or milk.*  *… genetic engineering for the development of new medicines and vaccines to improve human health (e.g. human insulin)*  *…* genetic engineering to human beings, or *to their cells and tissues, for various purposes such as detecting, or curing diseases, and characteristics we might have inherited from our parents and research on human beings*, medicines and vaccines.  - How much do you agree that …?  *… There should be clear ethical rules indicating when biotechnology/genetic engineering may not in any way be applied to human beings*  *… There should be clear ethical rules indicating when biotechnology/genetic engineering may not in any way be applied to animals*  *… There should be clear ethical rules indicating when biotechnology/genetic engineering may not in any way be applied to plants*  *… Only traditional breeding methods should be used, rather than changing the hereditary characteristics of plants or animals through biotechnology/genetic engineering*  *… One should look for a balance between animal welfare and human welfare*  *… If we do not protect the natural environment, human beings will not be able to survive in the future*  *… Traditional breeding methods can be as effective as biotechnology/genetic engineering, in changing hereditary characteristics of plants and animals*  *… Applying biotechnology/genetic engineering to animals is morally acceptable, provided that the animals' welfare is safeguarded.*  *… It is acceptable for the development of life-saving drugs, even at the cost of some animal suffering.*  *… Public authorities should examine this application of biotechnology/genetic engineering case by case before deciding whether to allow it.*  *… Applying biotechnology/genetic engineering to animals is morally unacceptable and should be banned by public law.* | \| **Knowledge** \| *Mean* \| \| --- \| --- \| \| *Elementary (0-6)* \| 4.1/6 \| \| *Thorough (0-6)* \| 1.97/6 \| \| *Subjective (0-10)* \| 5.5/10 \| \| *Objective (0-12)* \| 6.53-6.73/12 \|   - Pessimists have a similar objective knowledge to optimists  - Elementary knowledge is much higher than thorough knowledge  - Subjective knowledge is lower than objective knowledge   \| **To what extent do you agree with research on…** \| *Mean (1991)* \| *Mean (1993)* \| \| --- \| --- \| --- \| \| *Plants* \| 0.98 \| 0.82 \| \| *Farm animals* \| -0.1 \| -0.06 \| \| *Food to improve food and drink quality* \| 0.47 \| 0.4 \| \| *Microorganisms to produce food products* \| 1.17 \| 1.12 \| \| *Microorganisms for break down waste products* \| 1.57 \| 1.45 \| \| *Developing medicines and vaccines* \| 1.59 \| 1.47 \| \| *Human beings to detect and cure diseases* \| 1.04 \| 0.93 \|   - Similar support exists for every application between 1991 and 1993 although it has decreased in 2 years  - Despite a slight increase, genetic engineering in farm animals is still the least accepted  - Global support of applications is close to 1 (-2 to +2) and therefore is worthwhile and should be encouraged  - Genetic engineering in human beings is supported averagely compared with new medicines and vaccines production  - Global support is positively influenced by objective knowledge  - Similarly to 1991, risk perception is the inverse of support for these applications  - A big majority (51-67%) say that these applications may involve risks to human health and environment   \| **How much do you agree that…** \| *Mean*  *(-2 to +2)* \| \| --- \| --- \| \| *… There should be clear ethical rules indicating when biotechnology/genetic engineering may not in any way be applied to human beings* \| 1.68 \| \| *… There should be clear ethical rules indicating when biotechnology/genetic engineering may not in any way be applied to animals* \| 1.52 \| \| *… There should be clear ethical rules indicating when biotechnology/genetic engineering may not in any way be applied to plants* \| 1.07 \| \| *… Only traditional breeding methods should be used, rather than changing the hereditary characteristics of plants or animals through biotechnology/genetic engineering* \| 0.73 \| \| *… One should look for a balance between animal welfare and human welfare* \| 1.57 \| \| *… If we do not protect the natural environment, human beings will not be able to survive in the future* \| 1.64 \| \| *… Traditional breeding methods can be as effective as biotechnology/genetic engineering, in changing hereditary characteristics of plants and animals* \| 0.71 \| \| *… Applying biotechnology/genetic engineering to animals is morally acceptable, provided that the animals' welfare is safeguarded.* \| 31 (100) \| \| *… It is acceptable for the development of life-saving drugs, even at the cost of some animal suffering.* \| 13 (100) \| \| *… Public authorities should examine this application of biotechnology/genetic engineering case by case before deciding whether to allow it.* \| 28 (100) \| \| *… Applying biotechnology/genetic engineering to animals is morally unacceptable and should be banned by public law.* \| 20 (100) \|   - 20% think applying genetic engineering to animals is morally unacceptable compared to 31% who think the opposite  - Only 13% see the cost of animal suffering on development of life-saving drugs  - Around a third (28%) say public authorities should examine this on a case-by-case  - More than average (0.73) say that only traditional breeding methods should be used and not changing hereditary characteristics and similarly (0.71) say both methods are as effective  - A big majority say that a balance between animal and human welfare should be seek (1.58) and that ethical guidelines should define human genetic engineering (1.68)  - A large majority (1.64) also says that the environment should be protected in order for humans to live there |
| Macer DRJ, Akiyama S, Alora AT, Asada Y, Azariah J, Azariah H, et al, (1995)  [36] | NZ, AU, J, IN, IS, RU, TH | | **Awareness** of gene therapy, genetic engineering and enhancement  **Attitudes towards gene therapy** in:  - somatic and germline settings  - enhancement | 3-point scale   - Not heard of it, Heard of it, Could explain it to a friend (3)   4-point scale   - From very willing to very unwilling + D/K (4) - Qualitative | - If tests showed that *you were likely to get a serious or fatal genetic disease later in life, how willing* would you be to undergo therapy to have those genes corrected *before symptoms appear*?  - If you *had a child with a usually fatal genetic disease, how willing* would you be to have the child undergo therapy to have those genes corrected?  - "Can you tell me *how much you have heard or read* about each of these subjects?"  How willing would you be in using gene therapy if it was to…  *a. cure a usually fatal disease, such as cancer*  *b. reduce the risk of developing a fatal disease later in life*  *c. prevent children from inheriting a usually fatal disease*  *d. prevent children from inheriting a non-fatal disease, such as diabetes*  *e. improve physical characteristics that children would inherit*  *f. improve the intelligence level that children would inherit*  *g. make people more ethical*  *h. as an AIDS vaccine* | \| **Willingness to undergo gene therapy if you were likely to get a fatal disease later in life** \| \| \| \| \| \| \| \| \| \| \| \| --- \| --- \| --- \| --- \| --- \| --- \| --- \| --- \| --- \| --- \| --- \| \|  \| *NZ* \| *A* \| *J* \| *J (1991)* \| *IN* \| *TH* \| *RU* \| *IS* \| *US 92* \| *US 86* \| \| *Very willing* \| 47 \| 50 \| 42 \| 25 \| 61 \| 35 \| 23 \| 54 \| 30 \| 35 \| \| *Somewhat willing* \| 25 \| 27 \| 24 \| 29 \| 16 \| 20 \| 24 \| 16 \| 49 \| 43 \| \| *Somewhat unwilling* \| 9 \| 6 \| 15 \| 18 \| 5 \| 11 \| 6 \| 4 \| 9 \| 12 \| \| *Very unwilling* \| 4 \| 4 \| 6 \| 12 \| 4 \| 24 \| 13 \| 0 \| 9 \| 9 \| \| **Willingness to undergo gene therapy in a child if it had a usually fatal disease** \| \| \| \| \| \| \| \| \| \| \| \|  \| *NZ* \| *A* \| *J* \| *J (1991)* \| *IN* \| *TH* \| *RU* \| *IS* \| *US 92* \| *US 86* \| \| *Very willing* \| 60 \| 57 \| 53 \| 37 \| 73 \| 67 \| 35 \| 62 \| 52 \| 51 \| \| *Somewhat willing* \| 22 \| 25 \| 21 \| 29 \| 10 \| 16 \| 24 \| 18 \| 36 \| 35 \| \| *Somewhat unwilling* \| 3 \| 2 \| 10 \| 11 \| 3 \| 2 \| 5 \| 2 \| 5 \| 7 \| \| *Very unwilling* \| 2 \| 2 \| 1 \| 7 \| 3 \| 5 \| 6 \| 0 \| 4 \| 4 \|   **-** All publics from all countries are for the use of gene therapy both on a personal and their children level if a fatal disease is at stake  - India’s citizens are the most willing followed by Israel’s and Thailand’s public in both cases resembling US public in 86 and 92  - Japan’s, Australia’s and New Zealand’s citizens agree with it but in a lower level  - Australian and New Zealand respondents often mention reasons to save and increase life quality.  - Little concern about eugenics was evoked. The main reasons for disapproval were about health risk and unnaturalness  - “Improving genes” has been evoked by respondents in Thailand and India and by some in Russia and Japan despite these being negative in relation to eugenics  - Reasons to reject enhancement were also about unnaturalness, but also about Playing God, unpredictability and unnecessity  - Eugenics was mentioned when asked about benefits and risks of genetic engineering  - Quality of life and safety of medical intervention has been mentioned by was mentioned when talking about gene therapy   \| **Awareness to gene therapy** \| *NZ* \| *A* \| *J* \| *J (1991)* \| *IN* \| *TH* \| *RU* \| *IS* \| \| --- \| --- \| --- \| --- \| --- \| --- \| --- \| --- \| --- \| \| *Not heard of it* \| 26 \| 23 \| 23 \| - \| 27 \| 10 \| 22 \| 20 \| \| *Heard of it* \| 57 \| 50 \| 58 \| - \| 47 \| 70 \| 57 \| 48 \| \| *Could explain it to a friend* \| 17 \| 27 \| 19 \| - \| 26 \| 20 \| 21 \| 32 \|   - Gene therapy had a relatively average awareness among all respondents except Thailand that revealed higher awareness. Majority position themselves on the “heard of it”  - Genetic engineering was nevertheless the term more familiar among respondents   \| **Cure a usually fatal disease** \| *NZ* \| *A* \| *J* \| *IN* \| *TH* \| *RU* \| *IS* \| *US 86* \| *US 92* \| \| --- \| --- \| --- \| --- \| --- \| --- \| --- \| --- \| --- \| --- \| \| *Very willing* \| 58 \| 60 \| 42 \| 54 \| 78 \| 72 \| 58 \| 57 \| 48 \| \| *Somewhat willing* \| 30 \| 29 \| 41 \| 31 \| 18 \| 11 \| 30 \| 35 \| 30 \| \| *Somewhat unwilling* \| 4 \| 5 \| 3 \| 5 \| 1 \| 2 \| 10 \| 7 \| 5 \| \| *Very unwilling* \| 4 \| 3 \| 2 \| 4 \| 1 \| 7 \| 0 \| 7 \| 7 \| \| **Reduce the risk of developing a fatal disease later in life** \| *NZ* \| *A* \| *J* \| *IN* \| *TH* \| *RU* \| *IS* \| *US 86* \| *US 92* \| \| *Very willing* \| 42 \| 47 \| 35 \| 48 \| 50 \| 45 \| 50 \| 39 \| 41 \| \| *Somewhat willing* \| 36 \| 34 \| 40 \| 35 \| 32 \| 33 \| 34 \| 38 \| 37 \| \| *Somewhat unwilling* \| 9 \| 6 \| 5 \| 6 \| 12 \| 6 \| 14 \| 12 \| 11 \| \| *Very unwilling* \| 6 \| 5 \| 1 \| 4 \| 3 \| 7 \| 0 \| 9 \| 8 \| \| **Prevent children from inheriting a fatal disease** \| *NZ* \| *A* \| *J* \| *IN* \| *TH* \| *RU* \| *IS* \| *US 86* \| *US 92* \| \| *Very willing* \| 59 \| 63 \| 37 \| 63 \| 75 \| 68 \| 52 \| 51 \| 52 \| \| *Somewhat willing* \| 26 \| 24 \| 43 \| 24 \| 21 \| 17 \| 30 \| 33 \| 32 \| \| *Somewhat unwilling* \| 5 \| 5 \| 3 \| 6 \| 2 \| 5 \| 16 \| 8 \| 6 \| \| *Very unwilling* \| 5 \| 2 \| 1 \| 2 \| 1 \| 5 \| 0 \| 7 \| 7 \| \| **Prevent children from inheriting a non-fatal disease** \| *NZ* \| *A* \| *J* \| *IN* \| *TH* \| *RU* \| *IS* \| *US 86* \| *US 92* \| \| *Very willing* \| 43 \| 50 \| 25 \| 42 \| 63 \| 45 \| 40 \| 41 \| 28 \| \| *Somewhat willing* \| 34 \| 29 \| 37 \| 31 \| 28 \| 26 \| 30 \| 36 \| 38 \| \| *Somewhat unwilling* \| 11 \| 8 \| 15 \| 10 \| 6 \| 10 \| 20 \| 12 \| 18 \| \| *Very unwilling* \| 7 \| 5 \| 2 \| 8 \| 2 \| 11 \| 4 \| 9 \| 14 \| \| **Improve the physical characteristics that children would inherit** \| *NZ* \| *A* \| *J* \| *IN* \| *TH* \| *RU* \| *IS* \| *US 86* \| *US 92* \| \| *Very willing* \| 10 \| 15 \| 12 \| 36 \| 54 \| 16 \| 10 \| 16 \| 16 \| \| *Somewhat willing* \| 14 \| 13 \| 16 \| 27 \| 29 \| 20 \| 12 \| 28 \| 27 \| \| *Somewhat unwilling* \| 17 \| 19 \| 35 \| 11 \| 11 \| 12 \| 34 \| 22 \| 21 \| \| *Very unwilling* \| 47 \| 44 \| 16 \| 15 \| 3 \| 38 \| 42 \| 31 \| 33 \| \| **Improve intelligence level that children would inherit** \| *NZ* \| *A* \| *J* \| *IN* \| *TH* \| *RU* \| *IS* \| *US 86* \| *US 92* \| \| *Very willing* \| 11 \| 15 \| 13 \| 41 \| 48 \| 18 \| 6 \| 18 \| 17 \| \| *Somewhat willing* \| 13 \| 12 \| 13 \| 29 \| 26 \| 17 \| 16 \| 26 \| 25 \| \| *Somewhat unwilling* \| 21 \| 21 \| 35 \| 10 \| 16 \| 11 \| 32 \| 22 \| 20 \| \| *Very unwilling* \| 46 \| 41 \| 19 \| 13 \| 6 \| 38 \| 38 \| 31 \| 35 \| \| **Make people more ethical** \| *NZ* \| *A* \| *J* \| *IN* \| *TH* \| *RU* \| *IS* \| *US 86* \| *US 92* \| \| *Very willing* \| 14 \| 18 \| 14 \| 31 \| 68 \| 19 \| 10 \| - \| - \| \| *Somewhat willing* \| 13 \| 16 \| 10 \| 25 \| 18 \| 12 \| 22 \| - \| - \| \| *Somewhat unwilling* \| 12 \| 10 \| 32 \| 12 \| 11 \| 7 \| 24 \| - \| - \| \| *Very unwilling* \| 43 \| 34 \| 21 \| 16 \| 6 \| 44 \| 32 \| - \| - \| \| **As an AIDS vaccine** \| *NZ* \| *A* \| *J* \| *IN* \| *TH* \| *RU* \| *IS* \| *US 86* \| *US 92* \| \| *Very willing* \| 49 \| 52 \| 33 \| 49 \| 75 \| 74 \| 48 \| - \| - \| \| *Somewhat willing* \| 24 \| 28 \| 36 \| 22 \| 17 \| 11 \| 28 \| - \| - \| \| *Somewhat unwilling* \| 6 \| 8 \| 4 \| 6 \| 3 \| 2 \| 18 \| - \| - \| \| *Very unwilling* \| 7 \| 4 \| 0.3 \| 8 \| 2 \| 6 \| 2 \| - \| - \|   - Gene therapy to cure disease, reduce risk of developing a fatal disease later in life and preventing children from inheriting a fatal and non-fatal disease have very high support from all publics without exceptions  - As for improvement of physical and intelligence characteristics, only Thais and Indians support it and highly while US are somewhat willing in the majority. The remaining are against it  - India’s and Thailand’s citizens are the ones agreeing with this to make people more ethical  - little distinction made between inheritable and non-heritable gene therapy and thus between somatic and germline interventions |
| Marteau T, Michie S, Drake H, Bobrow M, (1995)  [50] | UK | | **Attitudes** to gene manipulation | Dichotomous  • Yes/No | - *(A) Gene manipulation to achieve eight one desirable characteristics in their children (table 1).* This question was taken from the Daily Telegraph survey.5 | \|  \| % agree \| \| \| --- \| --- \| --- \| \| **Implanting or changing genes for…** \| *1993* \| *1994* \| \| *… appearance/behavior of a child* \| 2 \| 5 \| \| *… alter aggressive behavior* \| 5 \| 18 \| \| *… alcoholism* \| 5 \| 18 \| \| *… intelligence* \| 5 \| 11 \| \| *… specific skills* \| 2 \| 7 \| \| *… homossexuality* \| 4 \| 10 \|   - All different applications experienced an increase in people agreeing with selection of genes from 1993 to 1994  - Alteration of aggressive behavior and alcoholism were the characteristics mostly approved for selection of genes and the ones that saw a higher rise in 1994. |
| Ng MAC, Takeda C, Watanabe T, Macer D (2000)  [43] | Japan | | **Awareness** of genetic engineering applications  **Attitudes** to biotechnology/genetic engineering in animals for food and medical applications  **Attitudes** to gene therapy in somatic and germline settings | 3-point scale   - From Will improve to Make Worse + D/K + N/A   Qualitative  Dichotomous  • Approve/Disapprove + D/K  4-point scale  • From Definitely agree to Definitely disagree  • From Very Willing to Very Unwilling | - "*What comes to mind* when you think about modern *biotechnology in a broad sense, that is, including genetic engineering?,”*  1. *Have you heard of this application?* 2. *How useful* do you find this application is for society? 3. *How risky* do you think this application is for society? 4. *How morally acceptable* do you think this application is? 5. All in all this *application should encouraged?*  - Q8. *If there was no direct risk to humans and only very remote risks to the environment, would you approve or disapprove of the environmental use of genetically engineered organisms designed to produce*...?   b) *Healthier meat*  f) *Cows which produce more milk*   - Q9. If tests showed that *you were likely to get a serious or fatal genetic disease later in life, how willing would you be to undergo therapy to have those genes corrected before symptoms appear*? - Q10. How do you feel about scientists changing the genetic makeup of human cells to…?   a. *Cure a usually fatal disease,*  such as cancer  b. *Reduce the risk of developing a fatal disease earlier in life*  c. *Prevent children from*  *inheriting a usually fatal disease*  d. *Prevent children from*  *inheriting a non-fatal disease,*  such as diabetes  e. *Improve the physical*  *characteristics that children*  *would inherit*  f. *Improve the intelligence* level that children would inherit  *g. Make people more ethical*  *h. As an AIDS vaccine* | \|  \| **GM mice for cancer research** \| \| \| \| **GM pigs for human transplant** \| \| \| \| \| --- \| --- \| --- \| --- \| --- \| --- \| --- \| --- \| --- \| \| Variables \| *1997* \| \| *2000* \| \| *1997* \| \| *2000* \| \| \| *a) Awareness* \| 62.6 \| \| 80.6 \| \| 42.6 \| \| 66.8 \| \| \| **+ agree/ ++ totally agree** \| **++** \| **+** \| **++** \| **+** \| **++** \| **+** \| **++** \| **+** \| \| *b) Useful* \| 27,4 \| 48,3 \| 28 \| 43.4 \| 16,2 \| 35,8 \| 13.7 \| 27.7 \| \| *c) Risky* \| 16,2 \| 29,7 \| 16.5 \| 22.2 \| 26,1 \| 32,8 \| 25.9 \| 23.4 \| \| *d) Acceptable* \| 10,4 \| 32,6 \| 11.1 \| 27.9 \| 5,5 \| 18,2 \| 7.3 \| 16.4 \| \| *e) Encouraged* \| 22,9 \| 41,5 \| 16.5 \| 31.5 \| 14 \| 34,2 \| 11 \| 18.3 \|   - Awareness about both applications grew markedly among Japan public from 1997 to 2000  - On the contrary, usefulness and support decreased slightly while risk and moral acceptance didn’t suffer much alteration   \|  \| **Healthier meat** \| \| **Cows which produce more milk** \| \| \| --- \| --- \| --- \| --- \| --- \| \| Approval \| *1993* \| *2000* \| *1993* \| *2000* \| \| *Yes* \| 57 \| 51.6 \| 44 \| 42.1 \|   - As for healthier meat, a decrease in approval has been experienced among respondents but still reaching the majority of Japan citizens  - Cows to produce more milk also suffered a decrease in approval and kept within the minority of the public as something to approve genetic engineering  - Unnaturalness concerns as well as unnecessity of these interventions and lack of predictability were evoked by Japan public   \| **Cure a usually fatal disease** \| *1993* \| *2000* \| \| --- \| --- \| --- \| \| *Very willing* \| 42 \| 38.3 \| \| *Somewhat willing* \| 31 \| 34.4 \| \| *Somewhat unwilling* \| 3 \| 13.1 \| \| *Very unwilling* \| 2 \| 4.6 \| \| **Reduce the risk of developing a fatal disease later in life** \| *1993* \| *2000* \| \| *Very willing* \| 35 \| 28.4 \| \| *Somewhat willing* \| 40 \| 34.8 \| \| *Somewhat unwilling* \| 5 \| 16.7 \| \| *Very unwilling* \| 1 \| 6 \| \| **Prevent children from inheriting a fatal disease** \| 1993 \| 2000 \| \| *Very willing* \| 37 \| 29.8 \| \| *Somewhat willing* \| 43 \| 36.2 \| \| *Somewhat unwilling* \| 3 \| 16.3 \| \| *Very unwilling* \| 1 \| 5.7 \| \| **Prevent children from inheriting a non-fatal disease** \| 1993 \| 2000 \| \| *Very willing* \| 25 \| 20.3 \| \| *Somewhat willing* \| 37 \| 31.7 \| \| *Somewhat unwilling* \| 15 \| 23.8 \| \| *Very unwilling* \| 2 \| 7.1 \| \| **Improve the physical characteristics that children would inherit** \| 1993 \| 2000 \| \| *Very willing* \| 12 \| 10.7 \| \| *Somewhat willing* \| 16 \| 12.5 \| \| *Somewhat unwilling* \| 35 \| 43.4 \| \| *Very unwilling* \| 16 \| 19.9 \| \| **Improve intelligence level that children would inherit** \| *1993* \| *2000* \| \| *Very willing* \| 13 \| 9.6 \| \| *Somewhat willing* \| 13 \| 10.3 \| \| *Somewhat unwilling* \| 35 \| 45.2 \| \| *Very unwilling* \| 49 \| 22.4 \| \| **Make people more ethical** \| *1993* \| *2000* \| \| *Very willing* \| 14 \| 10.4 \| \| *Somewhat willing* \| 10 \| 8.2 \| \| *Somewhat unwilling* \| 32 \| 33.7 \| \| *Very unwilling* \| 21 \| 30.1 \| \| **As an AIDS vaccine** \| *1993* \| *2000* \| \| *Very willing* \| 33 \| 26.7 \| \| *Somewhat willing* \| 36 \| 37.7 \| \| *Somewhat unwilling* \| 4 \| 16.4 \| \| *Very unwilling* \| 0.3 \| 5.3 \|   - All applications of genetic engineering and gene therapy suffered a decrease in willingness from the public to use it and increased unwillingness to be used.  - Nevertheless, the majority of Japan public envisages curing of diseases, reduction of risk of developing diseases later in life and prevent children from inheriting fatal and non-fatal diseases as applications where they willingly would use gene therapy  - On the other end are characteristics to be improved linked to physical, intelligence and making people more ethical |
| Macer DRJ, Azariah J, Srinives P (2000)  [44] | NZ, AU, J, IN, IS, RU, TH | | **Attitudes** to genetic engineering in:  - farm animals  - cross-species  **Attitudes towards gene therapy** in:  - somatic and germline settings  - enhancement | 3-point scale  • Not heard of it, Heard of it, Could explain it to a friend (3)  4-point scale  • From strongly approve to strongly disapprove + D/K (4)   - Qualitative   Dichtomous   - Yes/No - Acceptable/Unacceptable + D/K | - Q11. *Would chicken made less fatty through biotechnology be acceptable or unacceptable if genes were added to the chicken from another type of animal?* - Q12. *Would such chicken be acceptable or unacceptable if the genes came from a human*? - Q31. If there was no direct risk to humans and only very remote risks to the environment, would you approve or disapprove of the environmental use of genetically engineered organisms designed to produce...?   *b. Healthier meat (e.g. less fat)*  *f. Cows which produce more milk*   - Q28. *How do you feel about scientists changing the genetic makeup of human cells* to:   a. *Cure a usually fatal disease, such as cancer*  b. *Reduce the risk of developing a fatal disease later in life*  c. *Prevent children from inheriting a usually fatal disease*  d. *Prevent children from inheriting a non-fatal disease*, such as  diabetes  e. *Improve the physical characteristics that children would inherit*  f. *Improve the intelligence level that children would inherit*  g. *Make people more ethical*  h. *As an AIDS vaccine* | \| **Cure a usually fatal disease** \| *NZ* \| *A* \| *J* \| *IN* \| *TH* \| *RU* \| *IS* \| *US 86* \| *US 92* \| \| --- \| --- \| --- \| --- \| --- \| --- \| --- \| --- \| --- \| --- \| \| *Strongly approve* \| 58 \| 60 \| 42 \| 54 \| 78 \| 72 \| 58 \| 57 \| 48 \| \| *Somewhat approve* \| 30 \| 29 \| 41 \| 31 \| 18 \| 11 \| 30 \| 35 \| 30 \| \| *Somewhat disapprove* \| 4 \| 5 \| 3 \| 5 \| 1 \| 2 \| 10 \| 7 \| 5 \| \| *Strongly disapprove* \| 4 \| 3 \| 2 \| 4 \| 1 \| 7 \| 0 \| 7 \| 7 \| \| **Reduce the risk of developing a fatal disease later in life** \| *NZ* \| *A* \| *J* \| *IN* \| *TH* \| *RU* \| *IS* \| *US 86* \| *US 92* \| \| *Strongly approve* \| 42 \| 47 \| 35 \| 48 \| 50 \| 45 \| 50 \| 39 \| 41 \| \| *Somewhat approve* \| 36 \| 34 \| 40 \| 35 \| 32 \| 33 \| 34 \| 38 \| 37 \| \| *Somewhat disapprove* \| 9 \| 6 \| 5 \| 6 \| 12 \| 6 \| 14 \| 12 \| 11 \| \| *Strongly disapprove* \| 6 \| 5 \| 1 \| 4 \| 3 \| 7 \| 0 \| 9 \| 8 \| \| **Prevent children from inheriting a fatal disease** \| *NZ* \| *A* \| *J* \| *IN* \| *TH* \| *RU* \| *IS* \| *US 86* \| *US 92* \| \| *Strongly approve* \| 59 \| 63 \| 37 \| 63 \| 75 \| 68 \| 52 \| 51 \| 52 \| \| *Somewhat approve* \| 26 \| 24 \| 43 \| 24 \| 21 \| 17 \| 30 \| 33 \| 32 \| \| *Somewhat disapprove* \| 5 \| 5 \| 3 \| 6 \| 2 \| 5 \| 16 \| 8 \| 6 \| \| *Strongly disapprove* \| 5 \| 2 \| 1 \| 2 \| 1 \| 5 \| 0 \| 7 \| 7 \| \| **Prevent children from inheriting a non-fatal disease** \| *NZ* \| *A* \| *J* \| *IN* \| *TH* \| *RU* \| *IS* \| *US 86* \| *US 92* \| \| *Strongly approve* \| 43 \| 50 \| 25 \| 42 \| 63 \| 45 \| 40 \| 41 \| 28 \| \| *Somewhat approve* \| 34 \| 29 \| 37 \| 31 \| 28 \| 26 \| 30 \| 36 \| 38 \| \| *Somewhat disapprove* \| 11 \| 8 \| 15 \| 10 \| 6 \| 10 \| 20 \| 12 \| 18 \| \| *Strongly disapprove* \| 7 \| 5 \| 2 \| 8 \| 2 \| 11 \| 4 \| 9 \| 14 \| \| **Improve the physical characteristics that children would inherit** \| *NZ* \| *A* \| *J* \| *IN* \| *TH* \| *RU* \| *IS* \| *US 86* \| *US 92* \| \| *Strongly approve* \| 10 \| 15 \| 12 \| 36 \| 54 \| 16 \| 10 \| 16 \| 16 \| \| *Somewhat approve* \| 14 \| 13 \| 16 \| 27 \| 29 \| 20 \| 12 \| 28 \| 27 \| \| *Somewhat disapprove* \| 17 \| 19 \| 35 \| 11 \| 11 \| 12 \| 34 \| 22 \| 21 \| \| *Strongly disapprove* \| 47 \| 44 \| 16 \| 15 \| 3 \| 38 \| 42 \| 31 \| 33 \| \| **Improve intelligence level that children would inherit** \| *NZ* \| *A* \| *J* \| *IN* \| *TH* \| *RU* \| *IS* \| *US 86* \| *US 92* \| \| *Strongly approve* \| 11 \| 15 \| 13 \| 41 \| 48 \| 18 \| 6 \| 18 \| 17 \| \| *Somewhat approve* \| 13 \| 12 \| 13 \| 29 \| 26 \| 17 \| 16 \| 26 \| 25 \| \| *Somewhat disapprove* \| 21 \| 21 \| 35 \| 10 \| 16 \| 11 \| 32 \| 22 \| 20 \| \| *Strongly disapprove* \| 46 \| 41 \| 19 \| 13 \| 6 \| 38 \| 38 \| 31 \| 35 \| \| **Make people more ethical** \| *NZ* \| *A* \| *J* \| *IN* \| *TH* \| *RU* \| *IS* \| *US 86* \| *US 92* \| \| *Strongly approve* \| 14 \| 18 \| 14 \| 31 \| 68 \| 19 \| 10 \| - \| - \| \| *Somewhat approve* \| 13 \| 16 \| 10 \| 25 \| 18 \| 12 \| 22 \| - \| - \| \| *Somewhat disapprove* \| 12 \| 10 \| 32 \| 12 \| 11 \| 7 \| 24 \| - \| - \| \| *Strongly disapprove* \| 43 \| 34 \| 21 \| 16 \| 6 \| 44 \| 32 \| - \| - \| \| **As an AIDS vaccine** \| *NZ* \| *A* \| *J* \| *IN* \| *TH* \| *RU* \| *IS* \| *US 86* \| *US 92* \| \| *Strongly approve* \| 49 \| 52 \| 33 \| 49 \| 75 \| 74 \| 48 \| - \| - \| \| *Somewhat approve* \| 24 \| 28 \| 36 \| 22 \| 17 \| 11 \| 28 \| - \| - \| \| *Somewhat disapprove* \| 6 \| 8 \| 4 \| 6 \| 3 \| 2 \| 18 \| - \| - \| \| *Strongly disapprove* \| 7 \| 4 \| 0.3 \| 8 \| 2 \| 6 \| 2 \| - \| - \|   - Same conclusions as Macer et al, 1995   \| **Approve (if there was no direct risk for humans)** \| *NZ* \| *A* \| *J* \| *J (1991)* \| *IN* \| *TH* \| *RU* \| *IS* \| \| --- \| --- \| --- \| --- \| --- \| --- \| --- \| --- \| --- \| \| *Healthier meat* \| 54 \| 60 \| 57 \| - \| 66 \| 84 \| 35 \| 44 \| \| *Cows which produce more milk* \| 36 \| 39 \| 44 \| - \| 75 \| 84 \| 23 \| 38 \|   - India’s and Thailand’s citizens are the ones approving more of having healthier meat and cows which produce more milk if no direct risks for humans would be known.  - Japan, Australia and New Zealand respondents share a similar approval still reaching the majority for genetic engineering to obtain healthier meat  - Russia and Israel publics in their majority don’t approve of this and even less for cows to produce more milk and here, New Zealand, Australia and Japan public follow this trend   \| **Approval** \| *NZ* \| *A* \| *J* \| *J (1991)* \| *IN* \| *TH* \| *RU* \| *IS* \| *US* \| \| --- \| --- \| --- \| --- \| --- \| --- \| --- \| --- \| --- \| --- \| \| *Plant-plant* \| 56 \| - \| 39 \| - \| 56 \| 82 \| - \| - \| 66 \| \| *Animal-plant* \| 19 \| - \| 11 \| - \| 29 \| 48 \| - \| - \| 39 \| \| *Animal-animal* \| 29 \| - \| 20 \| - \| 40 \| 68 \| - \| - \| 25 \| \| *Human-animal* \| 10 \| - \| 6 \| - \| 16 \| 29 \| - \| - \| 10 \|   - Thailand public is the most accepting of any genetic engineering technique despite a big decrease seen from plant-plant to human-animal (82 to 29%))  - This approval falls for all countries and animal-animal is even more acceptable than animal-plant  - Human-animal genetic transfer receives the lowest approval among all publics reaching 30% tops for Thais and lower than 10 in the remaining except India  - Awareness about genetic engineering is very high and comparable to biotechnology (Thais and Indians here excel)  - Japanese are the most aware about “biotechnology”  - As for benefits and worries, biotechnology is more worrying seen by Australians and New Zealanders when compared with other countries surveyed and also follows on similar results seen for genetic engineering. They mention unnaturalness and human misuse |
| UK Human Genetics Comission (2001)  [48] | UK | **Awareness** of genetics  **Knowledge** on inherited and environmental diseases  **Attitudes** to new genetic developments and research on human genetics | | Qualitative  6-point scale  • Strongly agree, tend to agree, Neither/Nor, Tend to disagree, Strongly disagree, D/K (6)  Dichotomous  • Agree/Disagree + D/K  5-point scale  • From Totally Inherited to Totally Environmental (5) | - *Q1. When I say ‘genetics’, what if, anything, springs to mind?* - *Q4 Please tell me to what extent you agree or disagree …. New genetic developments will bring cures for many diseases?* - *Q6 Please tell me to what extent you agree or disagree … Research on human genetics is tampering with nature and is therefore unethical?* - *Q7 New genetic developments will mean children who are healthier and free from inherited disabilities* - *Q12- Q30. I am now going to read out a list of characteristics which may come about because they are inherited (nature) or because of environmental factors (nurture) such as lifestyle, upbringing etc. or because of a combination of these..* | - GM food has been pointed as the immediate thing that springs to mind when participants heard “genetics”   \|  \| *Strongly agree* \| *Tend to agree* \| *Neither* \| *Tend to disagree* \| *Strongly disagree* \| *D/K* \| \| --- \| --- \| --- \| --- \| --- \| --- \| --- \| \| *New development will bring cure for many diseases* \| 35 \| 53 \| 5 \| 4 \| 1 \| 2 \|   - 88% people say genetic developments will bring cures for many diseases  - High knowledge on genetics is positively correlated with support for it (92% agree with it)  - 75% think such genetic developments will mean healthier children without inherited disabilities   \|  \| *Agree* \| *Disagree* \| \| --- \| --- \| --- \| \| *Human genetic research is tampering with nature* \| 33 \| 41 \|   - More than 66% support the use of genetic information with the purpose to correct defective genes for individuals and future generations  - Small minorities support the use of this information to choose physical and mental characteristics |
| Cook AJ, Fairweather JR, Satterfield T, Hunt LM (2004)  [46] | NZ | **Awareness** to biotechnology  **Attitudes** towards biotechnology and GMOs in:  - agriculture  - environment  - medical | | 5-point scale  • From Very Unacceptable (1) to Very Acceptable (5)  • Strongly Disagree (1) to Strongly Agree (5) | 1. When asked to think broadly about the kind of issues facing society today, some people mention the following items. Please indicate how *concerned or unconcerned you are about each of the following. (Biotechnology and GMOs in agriculture)*  2. The following are a number of *environmental, agricultural and medical examples of biotechnology*. Based on your current knowledge, please indicate your opinion about the acceptability or unacceptability of each example.  *- Inserting human genes into a cow to produce milk for the treatment of multiple sclerosis*  *- Preventing stomach cancer by modifying a person’s genetic code*  4. Some people said the following when we invited them *to talk about biotechnology*. Please indicate your level of agreement or disagreement with each of the following  Statements *(Several statements about biotechnology)*  6. Some people express concern about who *benefits from biotechnology*. Based on your current knowledge, please indicate your level of agreement or disagreement with each of the following statements about who benefits from biotechnology.  7. Some people we have interviewed mentioned the *following concerns about biotechnology*. Please indicate your level of agreement or disagreement with each of the following concerns about biotechnology.  8. Please provide *your view of biotechnology as a whole given that it means the use of living things to solve problems and make products*. Based on your current knowledge, please indicate your level of agreement or disagreement with each of the following statements | \| **Feelings about…** \| *Very concerned/concerned* \| *Familiar* \| *Acceptable* \| \| --- \| --- \| --- \| --- \| \| *Biotechnology* \| 51.6 \| 29.3 \| 45.6 \| \| *GMOs agriculture* \| 57.6 \| - \| - \|  \| **Applications** \| Agree (%) \| \| --- \| --- \| \| *Human genes in a cow to treat multiple sclerosis* \| 46.9 \| \| *Prevent stomach cancer by modifying person’s genetic code* \| 44.9 \|   - The acceptance for these applications reaches lower than half of the New Zealand public   \| **Statements about biotech and GM** \| Agreement (%) \| \| --- \| --- \| \| *Biotechnology to fix environmental problems* \| 51.8 \| \| *GM plants and animals have right to live and reproduce* \| 30.1 \| \| *GM is part of evolution process* \| 44.4 \| \| *Genetic makeup of animals and humans is identical* \| 33.9 \| \| *Plant and animals genetic mix-up is wrong* \| 53.4 \| \| *GMOs will mutate into something dangerous* \| 44.1 \| \| *Biotechnology commercialization presents a risk to public and environment* \| 52.9 \| \| *Biotechnology can be controlled* \| 23.5 \| \| *Biotechnology will result in irreversible harmful consequences* \| 20.8 \| \| *Biotechnology is unethical* \| 51.9 \| \| *Biotechnology is unnatural* \| 42 \|   - Biotechnology is seen as something that represents some danger to both public and the environment (52.9%) despite a similar percentage of people considering it unethical (51.9%) and capable of fixing environmental issues (51.8%) at the same time  - GM is seen as part of the evolution process (44.4%) by almost half of people but fewer consider them with the right to live and reproduce (30.1%)  - GMOs are believed by almost half as something that will mutate into something dangerous (44.1%). Biotechnology is not seen as so bad in that case (20.8%)  - Genetic mix-up of animals and plants is seen as wrong by a majority (53.4%)  - 42% say biotechnology is unnatural and 23.5% say it can be controlled. |
| Evans MDR, Kelley J, Zanjani ED (2005)  [45] | AU | **Knowledge** of genetics  **Attitudes** to germline gene therapy | | 5-point scale  • From Definitely yes to Definitely Not (points) (5)  4-point scale  • Serious defect, minor defect, personality defect, eugenics (4)  Dichotomous   - Yes/No | 7. Another possibility *is ‘germ line therapy’ in which the genes associated with a serious defect would be changed so that:*  >*he would never get the disorder himself,*  >*he would not pass on the defective genes to his children* – instead, his new genes would also eventually be passed on to all of his descendants, changing the human race. That possibility worries many people.  a. If a *screening test finds a serious defect which would kill the child* in a few years, should the *parents be allowed to have the child’s genetic makeup* modified in this way?  b. If the genetic defect was only a *minor one, like a cleft palate*?  c. What if the *child is likely to be aggressive and violent*?  d. If the parents wanted a *good-looking child*? | \| **Germline gene therapy (GLGT) and abortion (A) …** \| *Definitely be allowed* \| \| *Should probably be allowed* \| \| *Neutral* \| \| *Should probably not be allowed* \| \| *Definitely not be allowed* \| \| \| --- \| --- \| --- \| --- \| --- \| --- \| --- \| --- \| --- \| --- \| --- \| \|  \| **GLGT** \| **A** \| **GLGT** \| **A** \| **GLGT** \| **A** \| **GLGT** \| **A** \| **GLGT** \| **A** \| \| *… for a serious defect* \| 16 \| 35 \| 24 \| 37 \| 23 \| 14 \| 20 \| 8 \| 16 \| 6 \| \| *… for a minor physical defect* \| 9 \| 5 \| 19 \| 12 \| 23 \| 19 \| 27 \| 39 \| 21 \| 25 \| \| *… for aggression and violence* \| 8 \| 7 \| 16 \| 15 \| 24 \| 32 \| 29 \| 29 \| 23 \| 17 \| \| *… for cosmetic enhancement* \| 3 \| 2 \| 6 \| 3 \| 18 \| 7 \| 30 \| 31 \| 43 \| 57 \|   - For serious defect, abortion is preferred versus GLGT and the opposite is true for minor physical defects  - For aggression and violence, both methods are comparable in terms of acceptance  - Cosmetic enhancement slightly less rejected when performing GLGT rather than abortion |
| Sturgis P, Cooper H, Fife-Schaw C (2005)  [49] | UK | **Knowledge** on general scientific knowledge (BSA) and genetics (WT)  **Attitudes** to gene therapy in general (WT) and in specific situations for:  - germline and somatic (BSA and WT)  - enhancement (BSA and WT) | | (BSA)  4-point scale 🡪 Dichotomous  *Dichotomous +1 means pro-science/biotech and 0 means against  • From Definitely Allowed to Definitely Not allowed (4) 🡪 +1/0  5-point scale 🡪 7-point scale (BSAS)  • From Definitely True to Definitely not true 🡪 Sum of all (7)  (WT)  3-point 🡪 10-  point scale  • True, False, D/K 🡪 Sum of all (10)  5-point scale 🡪 Dichotomous  • From agree strongly to Disagree strongly + D/K 🡪 +1/0  4-point scale 🡪 Dichotomous  (WT)  • From Definitely Allowed to Definitely not allowed 🡪 +1/0 | (BSA)   - Wordings and coding of items in measure of *general scientific knowledge*   *1. Antibiotics can kill bacteria but not viruses (true).*  *2. Human beings developed from earlier species of animals (true).*  *3. All man-made chemicals can cause cancer if you eat enough of them (false).*  *4. If someone is exposed to any amount of radioactivity, they are certain to die as a result (false).*  *5. The greenhouse effect is caused by a hole in the earth’s atmosphere (false).*  *6. Every time we use coal or oil or gas, we contribute to the greenhouse effect (true).*  Gene therapy  - Suppose it was discovered that a person’s genes could be changed. *Do you think that this should be allowed or not allowed to. . .?*  *...make a person taller or shorter*  *…make a person more intelligent*  *…make a person straight, rather than gay or lesbian*  *…reduce a person’s chances of getting heart disease*  *…make a person of average weight, rather than very overweight*  *… determine the sex of an unborn baby*  *… to give someone a full head of hair, rather than being bald*  *...to stop someone having schizophrenia*  *… to make them less aggressive or violent*  (WT)   - Wordings and coding of items in measure of *genetic knowledge*   *1. Identical twins have the same genes (true)*  *2. There are test tube babies who grew entirely outside the mother’s body (false)*  *3. Genes of all living things on earth are made up of different combinations of only 4 or 5 chemical building blocks (true).*  *4. Down’s Syndrome is an inherited disease (true)*  *5. Children look like their parents because they share the same type of red blood cells (false)*  *6. Half your genes come from your mother and half from your father (true)*  *7. Whether a couple have a girl or a boy depends on the woman’s genes (false)*  *8. We have around 150,000 different chromosomes which contain our genes (false)*  *9. Most cells in our body contain a copy of all our genes (true).*   - Wordings and coding of attitude items   *.[Agree] genetic treatments for illness will do a lot to reduce human suffering*  *.[Disagree] changing a person’s genes is too risky, whatever the benefits might be*  *.[Disagree] it is better to try to cure illness without changing people’s genes*  *.[Agree] that in the end, research into human genes will do more to help us than to harm us*  *.[Disagree] it would be better if we did not know how to change people’s genes at all*  *.[Agree] people worry too much about the risk of changing human genes*  *.[Disagree] we should never interfere with people’s gene*  .[Disagree] scientists should not look for genetic cures because the world will become too overpopulated  *.[Disagree] changing genes should be forbidden as it is tampering with nature*  .[Agree that it] should be allowed to test new genetic treatments on children  Specific attitudes to gene therapy  - I’d like you to think of [someone in their 20s who has serious heart disease/a man in his 20s who is bald and feels very embarrassed about it/someone in their 20s born with cystic fibrosis].  *Do you think it should be allowed if…*  *… These new genes would not be passed onto any children they might later have.(items 2a, 2d, 2g)*  …  *The new genes were passed onto their future children [to give them less chance of getting serious heart disease in their 20s?/so that they would not go bald in their 20s/so that they would not have cystic fibrosis]. (items 2b, 2e, 2h)*  *… a [person’s/man’s] genes could be changed before they were born—by treatment while still in their mother’s womb—[to give them less chance of getting serious heart disease in their 20s/to stop him going bald in his 20s/so they would not have cystic fibrosis]. The new genes would not be passed onto any children they later have. (items 2c, 2f, 2i)* | \| **Gene therapy to…** \| *% of agree (BSAS)* \| *Shift in opinion (when fully informed)* \| \| --- \| --- \| --- \| \| *… make someone taller or shorter* \| 25 \| +8 \| \| *… make someone more intelligent* \| 21 \| +3 \| \| *… make someone straight rather than gay or lesbian* \| 19 \| +1 \| \| *… reduce changes of heart disease* \| 67 \| +6 \| \| *… make someone of average height* \| 40 \| +4 \| \| *… determine sex of unborn baby* \| 15 \| 0 \| \| *… prevent baldness* \| 23 \| +3 \| \| *… stop schizophrenia* \| 68 \| +1 \| \| *… make someone less aggressive* \| 58 \| -7 \|  \| **Gene therapy to…** \| *% of agree (WT)* \| *Shift in opinion (when fully informed)* \| \| --- \| --- \| --- \| \| *… allow for somatic gene therapy for heart disease* \| 82 \| +5* \| \| *… allow germ therapy for heart disease* \| 64 \| -2 \| \| *… allow in-utero therapy for heart disease* \| 49 \| +10 \| \| *… allow somatic gene therapy for cystic fibrosis* \| 91 \| +5 \| \| *… allow germ therapy for cystic fibrosis* \| 80 \| +5 \| \| *… allow in-utero therapy for cystic fibrosis* \| 62 \| +8 \| \| *… allow somatic gene therapy for baldness* \| 64 \| 0 \| \| *… allow germ therapy for baldness* \| 41 \| -1 \| \| *… allow in-utero therapy for baldness* \| 20 \| -6 \|   - The only significant shift in opinion in terms of attitudes to gene therapy was seen for “allow for somatic gene therapy for heart disease”  - All applications except making someone less aggressive and prevention of baldness by germline and in-utero therapy experienced a positive theoretical influence of scientific knowledge (the more knowledge the more acceptable)  - All the others seem to show that fully informed public is not more prone to agree or disagree with gene therapy regarding any other applications and circumstances  - Gene therapy is seen as very much approved by the public in UK to allow for somatic gene therapy for any heart disease and cystic fibrosis and as widely approved by the majority to stop mental illnesses like schizophrenia and reduce chances of heart disease too.  - Making someone less aggressive also receives acceptance by the majority for the use of gene therapy  - Other physical and psychological characteristics however are seen as not acceptable by the UK public in its majority  - Germline therapy for diseases is seen as acceptable by a significant fraction of the public except to prevent baldness  - In-utero therapy is not very much accepted by the public to prevent baldness (only 20%) and just on average if the purpose is for diseases, in particular cystic fibrosis  - The application most rejected is the determination of the sex of unborn baby   \| **General atittudes** \| *% of (WT)* \| *Shift in opinion (when fully informed)* \| \| --- \| --- \| --- \| \| *Agree genetic treatments will reduce human suffering* \| 76 \| +8* \| \| *Disagree changing genes is too risky whatever benefits* \| 27 \| +14* \| \| *Disagree better to cure illness without changing genes* \| 11 \| +7 \| \| *Agree research into human genes will do more to help us than harm us* \| 13 \| +1 \| \| *Disagree better if not know how to change genes* \| 58 \| +19* \| \| *Agree people worry too much about risk of changing genes* \| 41 \| +7* \| \| *Disagree should never interfere with genes* \| 52 \| +20* \| \| *Disagree scientists should not look for genetic cures as world will be overpopulated* \| 68 \| 12* \| \| *Disagree changing genes should be forbidden as tampering with nature* \| 60 \| +26* \| \| *Agree with allowing new genetic treatments on children* \| 14 \| 11* \|   - In all attitudes a shift in opinion is seen when scientific knowledge in genetics is higher (the higher the knowledge in genetics the more likely to agree with these ideas)   \| **How many were correct?** \| BSA survey (%) \| WT survey (%) \| \| --- \| --- \| --- \| \| *0 answers* \| 32 \| 2 \| \| *1 answer* \| 25 \| 4 \| \| *2 answers* \| 19 \| 10 \| \| *3 answers* \| 13 \| 15 \| \| *4 answers* \| 6 \| 18 \| \| *5 answers* \| 3 \| 20 \| \| *6 answers* \| 2 \| 15 \| \| *7 answers* \| - \| 10 \| \| *8 answers* \| - \| 4 \| \| *9 answers* \| - \| 2 \|   - UK public failed most of the answers to the knowledge items in BSA survey (55% answered correctly 1 at the tops) about general scientific knowledge  - At the WT the distribution was normal with people having an average knowledge towards genetics |
| European Comission Directorate-General for Research.    (Eurobarometer 64.3)  (2005)  [41] | EU25 | **Knowledge** on biotechnology and genetic engineering  **Awareness** of gene therapy  **Attitudes** towards gene therapy in general | | 5-point scale  • From Totally agree to totally disagree  4-point scale   - From I approve of if… to I do not approve of - Totally inherited, totally spontaneous, both, depends   Dichotomous  • Yes/No  • True/False + D/K | - For each of the following statements, please tell me *whether you think it is true or false.*   1. It is possible to find out in the first few months of pregnancy whether a child will have Down's syndrome  2. The cloning of living things produces exactly identical offspring.  3. Yeast for brewing beer consists of living organisms.  4. More than half of the human genes are identical to those with chimpanzees  5. By eating a genetically modified fruit, a person's genes could also become modified.  6. Embryonic stem cells have the potential to develop into normal humans  7. Genetically modified animals are always bigger than ordinary ones.  8. Ordinary tomatoes do not contain genes, while genetically modified tomatoes do.  9. Human cells and human genes function differently from those in animals and plants  10. It is not possible to transfer animal genes into plants.   - There are differing views about whether people inherit particular characteristics or whether they acquire them from the conditions in which they live*. Please tell me whether you think each of the following characteristics is mainly inherited or mainly the result of living conditions.* - *Have you ever heard of gene therapy* which involves treating diseases by directly intervening with the genes themselves? - For each of the *following issues regarding gene therapy, please tell me if you agree or disagree with it. (risky, useful, morally acceptable, encouraged)* - Overall, which of the following *best describes your views about gene therapy?* | \|  \| % and scale (-1.5 – 1.5) \| \| \| --- \| --- \| --- \| \|  \| *Gene therapy* \| *GM foods* \| \| *Familiar* \| 45 \| 80 \| \| *Morally acceptable* \| ~0.35* \| ~-0.2* \| \| *Useful* \| ~0.4* \| ~-0.2* \| \| *Risky* \| ~0.15* \| ~0.3* \| \| *Encouraged* \| ~0.3* \| ~-0.45* \| \| *Support* \| 50 \| 27 \| \| *Outright supporters* \| 44 \| 25 \| \| *Risk tolerant supporters* \| 36 \| 17 \| \| *Opponents* \| 20 \| 58 \|   *All values are approximated  - Gene therapy is considered mostly as useful for society and morally acceptable in its majority being perceived as risk-tolerable but to be encouraged nevertheless  - People are much more aware of GM foods than gene therapy but the support is much lower (27% vs 50%)  - GM foods are perceived as risky and negatively perceived as useful, acceptable or to encouraged  - In that sense, most of people are opponents to GM foods and gene therapy receives the biggest fraction of outright supporters despite not reaching half of the people still due to the one-third of risk-tolerant supporters representation  - Those who are more aware of gene therapy express more positive views about it and the contrary is seen for GM foods  - Majority of Europeans have heard about biotechnology and 71% would read or watch programmes about this subject. Still, 40% (the largest group) are unengaged  - The more active and attentive the European citizen is the more concerned he/she is about biotechnology  - Scientific delegation principle is mentioned mostly by attentive and active citizens on the matter of biotechnology who are also more optimistic about it  - New EU10 countries belonging now to EU25 trust more on their actors and institutions and are as most supportive of applications on medical, agricultural and industrial biotechnologies as the old EU15 countries   \|  \| **% correct** \| \| \| \| \| --- \| --- \| --- \| --- \| --- \| \| **Knowledge statements** \| *1996* \| *1999* \| *2002* \| *2005* \| \| 1. It is possible to find out in the first few months of pregnancy whether a child will have Down's syndrome \| 81 \| 79 \| 79 \| 79 \| \| 2. The cloning of living things produces exactly identical offspring. \| 46 \| 64 \| 66 \| 68 \| \| 3. Yeast for brewing beer consists of living organisms. \| 68 \| 66 \| 63 \| 65 \| \| 4. More than half of the human genes are identical to those with chimpanzees \| 51 \| 48 \| 52 \| 62 \| \| 5. By eating a genetically modified fruit, a person's genes could also become modified. \| 48 \| 42 \| 49 \| 54 \| \| 6. Embryonic stem cells have the potential to develop into normal humans \| n/a \| n/a \| n/a \| 51 \| \| 7. Genetically modified animals are always bigger than ordinary ones. \| 36 \| 34 \| 38 \| 45 \| \| 8. Ordinary tomatoes do not contain genes, while genetically modified tomatoes do. \| 35 \| 35 \| 36 \| 41 \| \| 9. Human cells and human genes function differently from those in animals and plants \| n/a \| n/a \| n/a \| 34 \| \| 10. It is not possible to transfer animal genes into plants. \| 27 \| 26 \| 26 \| 31 \|   - Knowledge about genetics has increased over 10 years of questioning as it can be seen by the increase in correct answers on almost all questions asked (only 1 doesn’t reflect it) |
| Sato H, Akabayashi A, Kai I (2006)  [87] | Japan | **Awareness** of gene therapy  **Opinions on** gene therapy | | Time-point scale | - The questionnaire asked for the period of first issue attention *(when did you first hear of the issue on organ transplant and on gene therapy?), and for the period of opinion formation (when did you arrive at the opinions you have now on these issues?).*   *- before 1991 (when the MHW created an expert panel);*  *- around 1993 (when the MHW announced its guideline);*  *- 1994 (when the MESC publicized its guideline);*  *- around 1995 (when the first gene therapy was conducted at Hokkaido University);*  *- around 1997 (when the first therapy was announced as successful); and know for the first time by the survey.*  *- 2002 (when guidelines were revised)*  *- 2005* | \|  \| *Public awareness* \| *Opinion formation* \| \| --- \| --- \| --- \| \| *1991 guideline comm* \| 9.3 \| n.a. \| \| *1993 MHW guideline* \| 8.7 \| 6.0 \| \| *1994 MOEd guideline* \| 3.1 \| n.a. \| \| *1995 first (ADA) therapy* \| 21.3 \| 11.2 \| \| *1997 first therapy success* \| 24.7 \| 26.8 \| \| *2002 guideline revised* \| n.a. \| 44.0 \| \| *2005* \| 32.8 \| 56 \|   - Public awareness were very low until the first gene therapy has been conducted under the official guidelines  - Opinion formation increased greatly after the first gene therapy success and again after revision of the guidelines  - Public awareness increase seems to be related with opinion formation of the public besides people remaining very undecided at this time (56% formed an opinion) |
| Barnett J, Cooper H, Senior V (2007)  [85] | UK | **Awareness** of genes and genetics  **Attitudes** to gene therapy | | 4-point scale  • From Definitely not Allowed (1) to Definitely Allowed (4)  5-point scale   - From a ‘a great deal’ (1) to ‘not at all’ (5) | - *Four items probed whether or not gene therapy should be allowed for the following purposes*   *1: to lessen aggression or violence;*  *2: alter sexuality;*  *3: reduce chances of getting breast cancer;*  *4: determine the sex of an unborn baby.*  We assessed public attentiveness to issues concerning genes and genetics by combining *four items that asked respondents the extent to which they had heard or read about such issues, talked about them, or thought about them in the past few months.*  - a great deal  - a small amount  - not at all  Which items influence allowing gene therapy  *- Belief in public efficacy*  *- Attentiveness*  *- Education*  *- Trust in government*  *- Trust in those in charge* | \|  \| *Interest* \| *Heard/Read about* \| *Talked about* \| *Thought about* \| \| --- \| --- \| --- \| --- \| --- \| \| *A great deal* \| 24 \| 36 \| 14 \| 21 \| \| *A small amount* \| 25 \| 30 \| 27 \| 26 \| \| *Not at all* \| 51 \| 33 \| 59 \| 53 \|   - The majority of people have no interest at all in genes and genetics, don’t talk about it and don’t think about it  - The more divided is the awareness in terms of having heard or read about it  - The least that people do is talking about genes and genetics   \|  \| *Allow gene therapy (significant)* \| \| --- \| --- \| \| *Belief in public efficacy* \| -0.58 \| \| *Attentiveness (high)* \| -0.136 \| \| *Education (higher)* \| -0.603 \| \| *Trust in government rules* \| 0.761 \| \| *Trust in those in charge* \| 0.319 \|   - Interestingly, the higher the belief in public involvement, the attentiveness (awareness and interest) on genetics and the levels of education, the lower the endorsement for gene therapy  - On the other hand, trust in government rules and the ones in charge reveals a favoring to allow for gene therapy |
| European Comission Directorate-General for Research  (Eurobarometer 73.1) (2010)  [42] | EU27 | **Attitudes** to gene therapy and putting human genes in animals | | 4-point scale  • You fully approve and do not think that special laws are necessary, You approve as long as this is regulated by strict laws, You do not approve except under very special circumstances, You do not approve under any circumstances   - From totally agree to totally disagree | *Would you say that …?*  *… if scientists can put human genes into animals that will produce organs and tissues for transplant into humans, such as pigs for transplants or to replace pancreatic cells to cure diabetes*  … if *scientists also work on gene therapy which involves treating inherited diseases by intervening directly in the human genes themselves.*  *… if regenerative medicine is not only about developing cures for people who are ill. It is also looking into ways of enhancing the performance of healthy people, for example to improve concentration or to increase memory*  QB10a Now I would like to know whether you agree or disagree with each of the following issues regarding regenerative medicine.  *5. Mixing animal and human genes is*  *unacceptable even if it helps*  *medical research for human health*  *- Overall how strongly would you say you feel about issue concerning biotechnology?* | \|  \| *Fully approve* \| *Approve if strict laws to regulate* \| *Do not approve unless special circumstances* \| *Do not approve under any circumstances* \| *D/K* \| \| --- \| --- \| --- \| --- \| --- \| --- \| \| *Human enhancement* \| 11 \| 44 \| 20 \| 17 \| 7 \| \| *Xenotransplantation* \| 11 \| 46 \| 18 \| 17 \| 7 \| \| *Gene therapy* \| 11 \| 52 \| 18 \| 11 \| 8 \|   - Gene therapy is approved by almost two-thirds of EU27 citizens  - Similarly, these percentage is seen for both xenotransplantation and human enhancement  - These applications share nonetheless the same type of approval: the majority approves it if there are strict laws to regulate it   \|  \| *Fully approve* \| *Approve if strict laws to regulate* \| *Do not approve unless special circumstances* \| *Do not approve under any circumstances* \| *D/K* \| \| --- \| --- \| --- \| --- \| --- \| --- \| \| *Gene therapy 2010* \| 11 \| 52 \| 18 \| 11 \| 8 \| \| *Gene therapy 2005* \| 18 \| 36 \| 20 \| 8 \| 18 \|   - Gene therapy approval increased in 5 years with the percentage of people approving of it raising particularly in the case if there are strict laws to regulate it.  - It actually seems that these people became more decided with time (from 18 to 8): reflected on percentage of fully approve that dropped and the ones not approving it also increasing slightly   \| **Application** \| *Totally agree* \| *Tend to agree* \| *Tend to disagree* \| *Totally disagree* \| *D/K* \| \| --- \| --- \| --- \| --- \| --- \| --- \| \| *Mixing animal and human genes is unacceptable* \| 27 \| 26 \| 25 \| 11 \| 11 \|   - A lower fraction of EU27 citizens think that mixing animal and human genes is acceptable  - The remaining respondents mostly agree that such application is unacceptable with only a quarter tending to disagree that this is unacceptable  - Almost half of people (45%) feels somewhat strongly about issues concerning biotechnology  - Research on regenerative medicine is seen as it should be stopped if there are risks for future generations: 58% think like this  - Similarly, EU27 don’t support regenerative medicine developments if only benefits rich people: 74% and are undecided if it only benefits few people: 43% vs 45% |
