# Supplementary material for: What do people think about genetic engineering? A systematic review of questionnaire surveys before and after the introduction of CRISPR
Source: Front Genome Ed. 2023 Dec 19;5:1284547. doi: 10.3389/fgeed.2023.1284547 (PMC10773783; doi:10.3389/fgeed.2023.1284547)
Supplement: Supplementary file 9 [file Table6.DOCX]

| **Authors (years)** | **Country** | **Topic** | **Scale** | **Questions** | **Answers** |
| --- | --- | --- | --- | --- | --- |
| Chikhazhe TL (2015)  [67] | NZ | **Familiarity** with GM technology  **Attitudes** to Genetic modification:  - Health (Diseases),  - GM medicine  - Animals  - milk product  - Transgenic and Cisgenic Genetic modification | 5-point Likert scale   - 1- Never heard of it, 5-very familiar   3-point scale   - Totally support of, support in some circumstances, Totally oppose it + D/K   5-point scale   - 1= Strongly agree to 5= Strongly disagree | On a scale 1-5, please rate *your familiarity with genetic modification.*  In terms of the use *of GM products for food production, I …*  In terms of the use *of GM products for medical applications, I ….*  *(agree)*  How much acceptable is to…?  *…. genetically modify animals (e.g. cows, sheep) for human benefit.*  *… genetically modify humans in order to cure or eradicate genetic diseases.*  *…. genetically modify humans in order to enhance human capabilities (i.e. physical and mental attributes or abilities).*  Does GM…?  *… poses a significant risk to the environment*  *… poses a significant risk to the health and safety of humans*  *… poses a significant risk to the health and safety of animals*  How do you feel towards…  *… eating food from GM animals.*  *… GM medicines are safety for humans to use.*  *… using medicines developed using GM technology*  What do you think about…?  *…. If you suffered from a disease that can be treated by GM milk in terms of buying this product.*  *… Cisgenic animals acceptance.* | \| **Familiarity (%)** \| *Never heard* \| *Rings a bell* \| *Just heard* \| *Familiar* \| *Very familiar* \| \| --- \| --- \| --- \| --- \| --- \| --- \| \| *GM* \| 8,5 \| 17,3 \| 24,9 \| 41,4 \| 7,9 \|   - Majority is familiar with genetic modification   \| **Support (%)** \| *Totally support* \| *Some circumstances* \| *Totally oppose* \| *D/K + Unsure* \| \| --- \| --- \| --- \| --- \| --- \| \| *GM food* \| 5 \| 47 \| 27 \| 20 \| \| *GM medicine* \| 21 \| 45 \| 16 \| 18 \|   - Majority support under some circumstances GM food and GM medicine   \| **Moral acceptance** \| *Strongly agree* \| *Agree* \| *Neutral* \| *Disagree* \| *Strongly disagree* \| *D/K* \| \| --- \| --- \| --- \| --- \| --- \| --- \| --- \| \| *GM animals for human benefit* \| 2,3 \| 11 \| 22,1 \| 24,1 \| 26,3 \| 14,2 \| \| *GM humans to cure diseases* \| 4,2 \| 13,6 \| 27,6 \| 15,6 \| 21,2 \| 17,6 \| \| *GM humans for enhancement* \| 1,7 \| 5,7 \| 22,4 \| 23,8 \| 31,4 \| 15 \|   - Moral acceptance is majorly neutral or disagreeing (strongly as well) for in GM for human purposes either for GM animals, GM humans for diseases and enhancements   \| **Risk** \| *Strongly agree* \| *Agree* \| *Neutral* \| *Disagree* \| *Strongly disagree* \| *D/K* \| \| --- \| --- \| --- \| --- \| --- \| --- \| --- \| \| *Environment* \| 14,7 \| 23,2 \| 26,1 \| 9,3 \| 2 \| 24,6 \| \| *Human health and safety* \| 15,3 \| 26,9 \| 24,6 \| 8,5 \| 2,8 \| 21,8 \| \| *Animal health and safety* \| 15,9 \| 26,9 \| 24,4 \| 8,8 \| 2 \| 22,1 \|   - 41% see a risk for both human and animal health and safety; 37% see a risk for the environment  - 11% don’t see a risk in any of these situations   \| **GM products** \| *Strongly agree* \| *Agree* \| *Neutral* \| *Disagree* \| *Strongly disagree* \| *D/K* \| \| --- \| --- \| --- \| --- \| --- \| --- \| --- \| \| *Feel good eating GM animal food* \| 2,3 \| 8,2 \| 22,1 \| 26,9 \| 23,5 \| 17 \| \| *GM medicines are safe* \| 2 \| 13,3 \| 30,3 \| 9,1 \| 7,6 \| 37,7 \| \| *Feel good using medicines GM-derived* \| 2,8 \| 16,7 \| 32 \| 14,7 \| 10,8 \| 22,9 \|   - Only 10% would feel good about eating GM animal-derived food and 18% would feel good about using GM-derived medicines  - Cisgenics and GM for reduction in heart diseases in 10% is neutral  - Intention to purchase GM products has very lower agreement and a higher disagreement among responders |
| McCaughey T, Sanfilippo PG, Gooden GEC, Budden DM, Fan L, Fenwick E, et al (2016)  [72] | Global | **Awareness** to gene editing  **Attitudes** to Genetic editing:  - Somatic  children/adults  - Germline  Embryos  - Disease and non-disease | 5-point Likert scale   - Strongly Agree, Agree, Neutral, Disagree, Strongly Disagree, I don’t Know   Dichotomous   - Yes, No - Qualitative | *How much do you agree with the use of genetic editing of cells in ...*  *.... children or adults to cure a life threatening disease?*  *... children or adults to cure a debilitating disease?*  *... embryos to prevent a life threatening disease?*  *... embryos to prevent a debilitating disease?*  *... embryos to alter any non-disease characteristic?*  *How much do you agree with the use of genetic editing of cells in embryos to alter any non-disease characteristic - such as memory, eye colour or height?* This would mean that all subsequent generations would have the same genetic characteristics.  If you could …  *… safely genetically edit your embryo (…) to determine physical appearance* (eye colour; hair colour; skin colour)?  *… safely genetically edit your embryo (…) to determine intelligence?*  *… safely genetically edit your embryo (…) to determine strength or sporting ability?*  *What other non-health related traits would you edit?* [*Free text response]*  *How much do you agree with the use of genetically modified food?*  7 We are also interested in understanding the reasons for your answers.  Please describe the factors that have influenced your response or attitude towards human gene engineering. [*Free text response]* | \| **Gene editing agreement (%)** \| *Disagree* \| *Neutral* \| *Agree* \| \| --- \| --- \| --- \| --- \| \| *Children/adults to cure life-threatening diseases* \| 10 \| 31 \| 59 \| \| *Children/adults to cure a debilitating disease* \| 10 \| 30 \| 59 \| \| *Embryos to prevent life-threatening disease* \| 12 \| 26 \| 63 \| \| *Embryos to prevent a debilitating disease* \| 11 \| 25 \| 63 \| \| *Embryos to alter non-disease characteristic* \| 43 \| 30 \| 27 \|   **-** All applications with health purposes (cure/prevent diseases) are seen as reasons to agree with gene editing regardless of being children or adults  - Embryo editing is even more favoured than editing of children/adults due  - Non-health related purposes received the lowest support reaching 43% of disagreement among people.  - Of the 27% that agreed with it, pointed intelligence as the highest acceptance trait (68%) for it followed by physical strength and sporting ability (58.4%) and lastly, by appearance (51%) |
| STAT and Harvard T.H. Chan School of Public Health (2016)  [74] | US | **Attitudes** to changing genetic characteristics to unborn babies and gene therapy  **Familiarity** with changing genetic characteristics of unborn babies for health and non-health purposes  **Attitudes** to changing genetic characteristics of unborn babies for health and non-health purposes | Dichotomous   - Should be legal/Should be illegal   4-point scale   - Quite a lot to Nothing at all + D/K | *What does the public think about changing the genetic characteristics of unborn babies?*  *- to reduce risk of serious diseases*  *- to improve intellingence/physical attributes*  *- Does the public think the government should fund research on changing the genes of unborn babies?*  ~~-~~ *Do you think the FDA should or should not approve gene therapy treatments in US?*  - *Does the public think the government should fund research on gene therapy?*  *How much have you heard about changing of genetic characteristics in unborn babies?* | \| **Embryo editing (From the ones that heard)** \| *Legal* \| *Illegal* \| *Fund* \| *Not fund* \| \| --- \| --- \| --- \| --- \| --- \| \| *Reduce risk of developing serious diseases* \| 26 \| 65 \| 44 \| 51 \| \| *Improve intelligence or physical attributes* \| 11 \| 83 \| 14 \| 82 \|   - The most familiar respondents with embryo editing (41%) had a double likelihood in agreeing to gene modification in unborn babies to reduce risk of developing serious diseases from a legal point-of-view  - 59% agree that FDA should approve gene therapy in US against 30% who disagree with this view   \| How much have you heard/read \| Quite a lot \| Some \| Not much \| Nothing at all \| D/K or Refused \| \| --- \| --- \| --- \| --- \| --- \| --- \| \| Unborn babies \| 8 \| 23 \| 27 \| 42 \| * \|   - Majority of people inquired (42%) heard nothing at all about changing unborn babies’ genetics |
| Funk C, Kennedy B, Sciupac E  Pew Research Center (2016)  [76] | US | **Familiarity** with gene editing  **Atittude** to gene editing in germline settings | 4-point scale  Very, Somewhat, Not too much, Not at all (Worried/Enthusiastic)   - More acceptable, less acceptable, no diff - From Yes, I would definitely to No, I would definitely not + N/A   3-point scale   - A lot, a little, Not at all - A great deal to Not too much   Dichotomous   - Appropriate/Too far + N/A - Yes, likely/No, not likely + N/A - Acceptable/Unacceptable + Not sure - Qualitative | - How ENTHUSIASTIC are you, if at all, about this possibility for society as a whole?  - How WORRIED are you, if at all, about this possibility for society as a whole?  - Would you say MOST PEOPLE would want or would not want this gene-editing for their baby?  - *How much have you heard or read about gene editing of unborn babies?*  - If this gene-editing becomes available, giving HEALTHY babies a *much reduced risk of serious diseases and conditions*, *how much, if at all, do you think society as a whole would change?*  Do you think using *this gene-editing giving HEALTHY babies a much reduced risk of serious diseases and conditions is* …  … *Morally acceptable/Unacceptable*  *- Can you explain why?*  - Would this gene-editing giving HEALTHY babies a *much reduced risk of serious diseases and conditions be more acceptable, less acceptable, or would it make no difference* in each of these circumstances?  *- If people could choose which diseases and conditions are affected*  *- If the effects were permanent and could not be reversed*  *- If the effects were limited to that person and NOT passed on to future generations*  *- If it changed the genetic make-up of the whole population for the foreseeable future*  *- If it required testing on human embryos in order to develop these techniques*  - Gene editing to give healthy babies a much reduced risk of serious diseases and conditions would have  *… More benefits*  *… More downsides*  *… About equal benefits and downsides*  - If gene editing becomes available to give healthy babies a reduce risk of disease, would you…?  *… think that it will be used before we fully understand?*  *… think that will lead to inequality?*  *… lead to superiority sense of feel?*  *… lead to make people more confident?*  *… lead to more innovative and problem-solving people*  *… lead to people more productive?*  *… lead to people always EQUALLY HEALTHY as the average person today*  *… lead to people MUCH HEALTHIER than the average person today*  *… lead to people FAR HEALTHIER than any human known-to-date* | \| **Babies gene editing** \| *Very* \| *Somewhat* \| *Not too much* \| *Not at all* \| \| --- \| --- \| --- \| --- \| --- \| \| *Enthusiastic* \| 15 \| 34 \| 30 \| 19 \| \| *Worried* \| 22 \| 46 \| 23 \| 8 \|   -50% are enthusiastic about gene editing in unborn babies while almost 70% are worried about it  - 48% would want gene editing to help prevent diseases for their babies and 50% would not want it   \| **Gene editing** \| *A lot* \| *A little* \| *Not at all* \| \| --- \| --- \| --- \| --- \| \| *Awareness* \| 9 \| 48 \| 42 \| \|  \| *A great deal* \| *Some* \| *Not too much* \| \| *Change for society* \| 46 \| 35 \| 17 \|   - Who heard the least about gene editing enhancements would want it for themselves  -28% say gene editing babies is morally acceptable against 30% who say it’s morally unacceptable despite the majority being unsure (40%)   \| **Gene editing to give healthy babies lower reduced risk of disease would be… (acceptable)** \| *More* \| *Less* \| *No difference* \| \| --- \| --- \| --- \| --- \| \| *People could choose the diseases* \| 41 \| 17 \| 39 \| \| *If effects were permanent* \| 19 \| 37 \| 41 \| \| *If effects could not be passed to future generations* \| 34 \| 23 \| 40 \| \| *If it changed whole population genetic makeup* \| 17 \| 49 \| 31 \| \| *If it required testing on human embryos* \| 11 \| 54 \| 32 \| \| *(benefits)* \| 36 \| 28 \| 33 \|   - It’s more acceptable to use gene editing in unborn babies if people could choose the diseases to select for, if effects could not be passed to future generations and if it would bring benefits  - It’s less acceptable to use gene editing if effects were permanent, if it changed the whole population and if it required testing on human embryos  -The big portion of people says it wouldn’t make a difference  - 36% say gene editing to give healthy babies less risk of developing serious diseases and conditions would bring more benefits than downsides (28%) while 33% say this would bring about equal of both   \| **Gene editing to give healthy babies lower reduced risk of disease will…** \| *% US adults* \| \| --- \| --- \| \| *Be used before we fully understand* \| 73 \| \| *Increase inequality* \| 70 \| \| *Lead to superiority sense of feel* \| 53 \| \| *Make people more confident* \| 52 \| \| *Lead to new innovation and problem-solving* \| 45 \| \| *Make people more productive* \| 32 \| \| *Lead to people far healthier* \| 42 \| \| *Lead to people much healthier* \| 52 \| \| *Lead always to people equally healthy* \| 54 \|   - The majority feels that gene editing to lower reduce risk of disease in healthy babies will be used before we fully understand, increase inequality, lead to people more superior in their own sense, make people more confident, much healthier or as equally healthy as others |
| Cormick C, Mercer R (2017)  [71] | Australia | **Awareness** of biotechnology, genetic modification and gene editing  **Attitudes** to GM animals products | - 4-point scale:   Know enough and could explain it; Have heard of it but know little or nothing; Haven’t heard; D/K   - Ranking scale (4-point in 10):   10-7; 6-4; 3-0; D/K | Q4a. For the following list of technologies could you please say whether… you have not heard of it, OR you have heard of it but know very little about or nothing about it, OR you know enough about it that you could explain it to a friend?  … Biotechnology  … Genetic modification/GMOs  … Gene editing  Q7. Now we’d like you to think about food. please indicate how willing you would be to eat the following  … Products from GM animals  Q23. Please indicate your level of support for the following science and technology developments  … Genetically-modified therapeutics or medicines  … Gene editing | \| **How much have you heard about…?** \| *Know enough and could explain it* \| \| \| *Not heard of it* \| \| \| *Have heard but know very little or nothing* \| \| \| *D/K* \| \| \| \| --- \| --- \| --- \| --- \| --- \| --- \| --- \| --- \| --- \| --- \| --- \| --- \| --- \| \| Years \| **2012** \| **2015** \| **2017** \| **2012** \| **2015** \| **2017** \| **2012** \| **2015** \| **2017** \| **2012** \| **2015** \| **2017** \| \| *Biotechnology* \| 23 \| 19 \| 17 \| 61 \| 55 \| 60 \| 15 \| 21 \| 20 \| 1 \| 6 \| 3 \| \| *GM* \| 43 \| 33 \| 30 \| 45 \| 48 \| 51 \| 11 \| 15 \| 15 \| 1 \| 4 \| 3 \| \| *Gene editing* \| 17 \| \| \| 39 \| \| \| 39 \| \| \| 4 \| \| \|   - Genetic modification is the one that is better known and can be better explained by Australian citizens although this awareness decreased slightly along the years  - People kept their levels of awareness about biotechnology very similar along 5 year-time with the majority haven’t heard of it  - On the other hand, gene editing features as the one that people didn’t hear of it and if they heard, they don’t know nothing about it with 39% people in these situations   \| **How much would you be…?** \| *10-7* \| \| *6-4* \| \| *0-3* \| \| *D/K* \| \| \| --- \| --- \| --- \| --- \| --- \| --- \| --- \| --- \| --- \| \|  \| 2015 \| 2017 \| 2015 \| 2017 \| 2015 \| 2017 \| 2015 \| 2017 \| \| *Willing to eat products from GM animals* \| 28 \| 29 \| 27 \| 32 \| 37 \| 33 \| 9 \| 7 \| \| *Supporting of GM medicines* \| 46 \| 49 \| 27 \| 29 \| 14 \| 14 \| 14 \| 9 \| \| *Supporting of gene editing* \| 37 \| \| 32 \| \| 21 \| \| 10 \| \|   - People support gene editing in their majority with more than two-thirds (37%) with one-fifth not supporting it  - A similar percentage of people support GM medicines with a quarter not supporting of it  - As for the eating products derived from GM animals, Australian citizens are very divided in their willingness to consume them  - None of these 2 shows big differences in their evolution along time |
| Chen, C, Liang, Z (2017)  [82] | China | **Attitudes** to gene editing in adults and children – somatic  For disease and non-disease settings | Dichotomous  • Yes/No  5-point scale  • Strongly oppose, oppose, undecided, support, strongly support | Would you approve gene editing therapy …  - If your children were likely to develop fatal genetic diseases, you would like to use gene editing to modify their gene.  - If a genetic test told you that you were likely to develop severe or fatal diseases, you would like to use gene editing to modify the gene.  How much would you approve the following gene editing applications?  *Treat genetic heart disease*  *Extend lifespan*  *Modify the gene mutation which causes cancer*  *Reduce the genetic risks of dementia*  *HIV prevention*  *Reduce the genetic risks of thalassemia*  *Prevent high cholesterol*  *Military application*  *Improve intelligence*  *Improve exercise capacity*  *Change skin color* | \| **Gene editing therapy** \| *% approval* \| \| --- \| --- \| \| *Own children to develop fatal disease* \| ~62 \| \| *You get severe/fatal disease* \| ~65 \|   - Gene editing therapy is accepted by majority either if its for the individual or own children   \| **Gene editing applications** \| *% approval* \| \| --- \| --- \| \| *Genetic heart disease* \| ~80 \| \| *Extend lifespan* \| ~75 \| \| *Modifying mutation causes cancer* \| ~75 \| \| *Reduce risk of dementia* \| ~75 \| \| *HIV prevention* \| ~75 \| \| *Reduce risk thalassemia* \| ~70 \| \| *Prevent high cholesterol* \| ~35 \| \| *Military* \| ~25 \| \| *Intelligence* \| ~25 \| \| *Exercise* \| ~25 \| \| *Change skin color* \| ~10 \|   - The highest approval for gene editing is related with health applications, being the approval very low for non-health related ones |
| Gaskell G, Bard I, Allansdottir A, da Cunha RV, Eduard P, Hampel J, et al (2017)  [78] | A, DK, D, H, I, IT, NL, PT, E, UK, US | **Attitudes** to Prenatal (Germline) and adult (Somatic)  In therapy and enhancement | 11-point scale   - (from –5 for No, definitely not” to +5 for “Yes, definitely”) | In our study, four vignettes in an experimental design combined two contexts and two recipient categories (Supplementary Note, section 2).    - The contexts were *therapy (curing a disease) and enhancement (improving memory and learning capacity)*. The recipient categories were *adult and prenatal.*  *“Do you think he/they made a morally acceptable decision?”* and *“In his/their shoes would you make the same choice?”*  - Each respondent read one of the four vignettes (*adult therapy, prenatal therapy, adult enhancement or prenatal enhancement*) assigned at random and was then asked: | \| **Gene editing for…** \| *Score (average)* \| \| --- \| --- \| \| *Adult therapy* \| 8 \| \| *Prenatal therapy* \| 6 \| \| *Adult enhancement* \| 2 \| \| *Prenatal enhancement* \| 0 \|   *Definitely not - 0*  *Neutral - 5*  *Yes, definitely - 10*  - Support is consistently greater for treatment than for enhancement overall among the 11 countries  - A higher and consistent support can be seen for adult than prenatal intervention  - Prenatal therapy is supported among the majority of the countries   \| **Gene editing for…** \| *Positive comments* \| \| --- \| --- \| \| *Adult therapy* \| 75 \| \| *Prenatal therapy* \| 60 \| \| *Adult enhancement* \| 26 \| \| *Prenatal enhancement* \| 11 \|   - For adult therapy: 75% of comments were positive and related with health, life quality, curing of diseases and benefits better outweighing the risks  - For prenatal therapy: Support is 60% and similar comments to  - Adult enhancement: 26% of positive comments with the negative ones being related with unnecessity of it and the risks and unknown consequences it might bring  - Prenatal enhancement: 11% of positive comments with the negative ones touching the unnaturalness and messing with nature as well as the same as for adult enhancement |
| Scheufele DA, Xenos MA, Howell EL, Rose KM, Brossard D, Hardy BW (2017)  [75] | US | **Knowledge** on factual questions about genetics  **Attitudes** to Somatic and germline therapy  **Attitudes** to treatment and enhancement | 5-point scale   - Definitely true=1 to Definitely False=5 - The scale is from 1 ‘Not at all f risky/beneficial’ to 5 ‘Very risky/beneficial’.   Trichotomized  - 0= <3 answers correct; 1=4 or 5; 2= 6-9  7-point scale  • Strongly disagree=1 to Strongly agree=7  *collapsed to 3-point scale  • Completely unacceptable=1 to completely acceptable=7   - from 1 ‘Not at all likely’ to 7 ‘Certain’. | *“How acceptable do you think it is to use gene editing to…*  *… somatic therapy*  *… germline therapy*  *… somatic enhancement*  *… germline enhancement*  *“How likely do you think it is that human gene editing will…*  *1) “. . . lead to discrimination against those who are or are not genetically edited?*”  *2)* “. . *. give some people too much power to change the course of human development?*”  3) “. . .*remove stigmas around birth defects and genetic diseases?”* (and “*How much do you agree . . . human gene editing . . .”*  *4) “. . .messes with nature”*  5) “. . *.can easily be used for the wrong purposes”*  *“How beneficial/risky do you think human gene editing will be for society as a whole?”*  *“How likely to do you think it is that human gene editing will …*  *1) “. . .help fix human health and disease?”*  2)“. . *. lead to unintended human health problems?”*  *Factual knowledge items:*  “Over time, human DNA has picked up pieces of DNA from different species and viruses that naturally mixed in with human DNA.” TRUE  “Personal behavior and environmental factors cannot change human DNA.” FALSE  “Ordinary tomatoes do not carry genes, but genetically modified tomatoes do.”  FALSE  “Scientists have changed more than 30 genetic characteristics of commercially available plants with gene editing.” TRUE  “Genetically modified crops can be legally grown in all parts of Europe.” FALSE  “Genetically modified foods are currently sold in supermarkets.” TRUE  “To date, no scientists have started human gene editing trials.” FALSE  “Some U.S. universities are currently fighting in court over who owns the patent for the gene editing technology CRISPR-Cas9.” TRUE  “According to scientists, human beings developed from earlier species of animals.”  TRUE | \| **Gene editing** \| *Agree* \| *Neither* \| *Disagree* \| \| --- \| --- \| --- \| --- \| \| *Somatic therapy* \| 64 \| 17 \| 19 \| \| *Germline therapy* \| 65 \| 17 \| 18 \| \| *Somatic enhancement* \| 39 \| 26 \| 35 \| \| *Germline enhancement* \| 26 \| 23 \| 51 \|   *values are approximated  - Gene editing therapy (somatic or germline) had the highest agreement among participants  - Gene editing enhancement (somatic but specially germline) had a high disagreement among participants   \| **Rejecters who believe that gene editing …** \| *Somatic physical* \| *Somatic mental* \| *Germline physical* \| *Germline mental* \| \| --- \| --- \| --- \| --- \| --- \| \| *Messes with nature* \| 78 \| 79 \| 78 \| 80 \| \| *Leads to Discrimination* \| 45 \| 50 \| 43 \| 45 \| \| *Remove stigmas around disease* \| 20 \| 19 \| 22 \| 18 \| \| *Used for wrong purposes* \| 87 \| 85 \| 88 \| 88 \| \| *Will give people too much power* \| 60 \| 61 \| 60 \| 62 \|   - Rejecters say gene editing in any form will be used for wrong purposes.  - A big majority also thinks it messes with nature  - A low fraction doesn’t think it will remove stigmas around disease  - The highest indecision is on discrimination and that it will give people much power   \| **Accepter of therapy who believe that gene editing …** \| *Somatic physical* \| *Somatic mental* \| *Germline physical* \| *Germline mental* \| \| --- \| --- \| --- \| --- \| --- \| \| *Is risky for society* \| 29 \| 25 \| 28 \| 27 \| \| *Is beneficial for society* \| 32 \| 35 \| 33 \| 33 \| \| *Will fix human health* \| 60 \| 63 \| 62 \| 62 \| \| *Will lead to new health problems* \| 40 \| 38 \| 39 \| 38 \|   - Accepters have a consistent opinion in situations asked with majority only seen in gene editing therapy to fix human health  - Risks and benefits are not very much hypothesized by participants  - Leading to new health problems is very divided as a consequence  - The ones showing high knowledge (more than 6 questions correct) show higher levels of support as well (76% support for treatment; 41% support for enhancement)  - 33,9% low knowledge; 26,9% average knowledge; 39,2% high knowledge |
| Weisberg SM, Badgio D, Chatterjee A (2017)  [83] | US | **Attitudes** to genetic modification   - Edit, Engineer, Modify, Hack, Surgery | 7-point scale   - -3 = Absolutely Not to 3= Absolutely Yes | - “Study 1: We designed 10 vignettes by crossing two variables: *risk (explicitly mentioned or not) and metaphor (Modify/Neutral, Edit, Engineer, Hack, Surgery)*. Should we be actively researching these technologies?  - “Study 2: We used the *same metaphors from Study 1, but all participants now read vignettes that included the sentence about risks*. In *Study 1, in the Risk condition, the risks were explicitly mentioned at the end of the vignette*. In *Study 2, for half the participants, the risks were presented as the second sentence in the vignette, before the metaphor (Risk-before);* for *the other half, the risks were presented at the end, after the metaphor as in Study 1 (Risk-after)* | - Genetic modification research shouldn’t be so much pursued if risk is known beforehand  - Risk has an effect when is unknown by participants, increasing gene editing acceptance   \| **How much do you agree with gene editing** (-3 = Absolutely Not to 3= Absolutely yes) \| *Genetic modification research* \| *Effect of risk* \| \| --- \| --- \| --- \| \| *Study 1 (Risk after)* \| 1.65 \| 1.43 \| \| *Study 1 (No risk)* \| - \| **1.87** \| \| *Study 2 (Risk before or after)* \| **1.38** \| 1.52 \| |
| Wang J-H, Wang R, Lee JH, Iao TWU, Hu X, Wang Y-M, et al (2017)  [81] | China | **Familiarity** with gene therapy  **Attitudes** to gene therapy | 7-point scale   - From strongly disagree to strongly agree   5-Likert scale   - From Never heard of it to Knot it very well - Qualitative | - Have you ever heard about gene therapy in the last 5 years?   - Do you agree…  … that gene therapy will be helpful in addressing health needs of patients over the next few years?  … that gene therapy poses significant ethical issues in terms of altering the human genome*? *Human genome is the complete set of genetic material for human.  How much do you agree that people should be allowed …  … If it is possible to cure people with fatal diseases by gene therapy,  … If it is possible to cure people with debilitating diseases* such as Alzheimer’s dementia and Parkinson’s disease, by gene therapy,  *Debilitating disease: those with debilitating disease can no longer perform daily functions like eating or bathing. … If you have a child with a usually fatal genetic disease, such as Down Syndrome, sickle cell anemia, muscular dystrophy, willing to have child undergo gene therapy to have their genes corrected?  … If gene therapy is able to change a child’s inherited characteristics by changing the child’s genetic structure in the womb before they were born and you were making the decision, would consider doing so to improve his/her general physical health?  … If gene therapy is able to change parent’s genes in order to have a smarter or better-looking child and you were making the decision, would consider to do so?  … that there is a reasonable chance that gene therapy will become a common treatment modality over the next few years?  … that Chinese government ought to fund scientific research on developing new gene therapy treatment?  - that Chinese government ought to approve gene therapy treatments for use in China?  - What is your main concern in terms of gene therapy applied to humans? (multiple choices)   - Passing genetic changes to offspring; High cost; Adverse medical side effects; Privacy; Going against nature; Going against religious belief | \| **Familiarity** \| *High* \| *Moderate* \| *Low* \| \| --- \| --- \| --- \| --- \| \| *Gene therapy* \| 29,9 \| 30,6 \| 39,5 \| \| *GM food* \| 83,4 \| 13,4 \| 3,2 \|   - Respondents are more familiar with GM food than gene therapy   \| **Gene therapy** \| *Disagree* \| *Neutral* \| *Agree* \| \| --- \| --- \| --- \| --- \| \| *Helpful – future* \| 10,1 \| 32,6 \| 57,3 \| \| *Ethical issues* \| 22,5 \| 36,6 \| 40,8 \| \| *Adults (fatal diseases)* \| 6,9 \| 12,5 \| 80,6 \| \| *Adults (debilitating diseases)* \| 9,7 \| 13,5 \| 76,8 \| \| *Children (fatal diseases)* \| 6 \| 11,3 \| 82,7 \| \| *Germline genetic modification* \| 23,2 \| 20,7 \| 56,1 \| \| *Genetic enhancement* \| 35,7 \| 24,5 \| 39,8 \| \| *Common treatment – future* \| 19,9 \| 33,4 \| 46,7 \| \| *Funding government* \| 8,3 \| 31,3 \| 60,4 \| \| *Legal support government* \| 12,1 \| 39,9 \| 47,9 \|   - Respondents agree the most with gene therapy for health-related settings and disagree more for enhancement purposes and modification of germline  - The most disagreeing is around ethical issues on gene therapy and as a common treatment in the future   \| **Concern of gene therapy** \| *% of public* \| \| --- \| --- \| \| *Passing genetics changes to offspring* \| 55,3 \| \| *High cost* \| 61,9 \| \| *Adverse medical side effects* \| 72,0 \| \| *Privacy* \| 28,4 \| \| *Going against nature* \| 60,0 \| \| *Going against religious belief* \| 7,9 \|   - Adverse medical side effects is the concern most cited by the public (72%) followed by high cost and going against nature (61,9 and 60%) |
| Hendriks S, Giesbertz NAA, Bredenoord AL, Repping S (2018)  [79] | The Netherlands | **Willingness** to use genome modification  Somatic and germline  Disease and non-disease | Dichotomous  • Yes/No | *Would you use…*  *1) modified wheat for individuals with gluten intolerance* (Barro et al., 2016; Shewry and Tatham, 2016);  *2) somatic modification for individuals with neuromuscular diseases* (Tabebordbar et al., 2016);  *3) germline modification to prevent passing on a neuromuscular disease* (Long et al., 2014; Liang et al., 2015*);*  *4) germline modification to introduce resistance to HIV* (Samson et al., 1996; Kang et al., 2016); *or*  *5) germline modification to increase intelligence* (intelligence is in part genetically determined (Plomin and Spinath, 2004)).  *Why would you use or not?* | \| **GM application** \| *Yes* \| *No* \| \| --- \| --- \| --- \| \| *Wheat (gluten)* \| 73.5 \| 26.5 \| \| *Somatic cells (Neuromuscular disease)* \| 85.2 \| 14.8 \| \| *Embryo (Neuromuscular disease)* \| 65.9 \| 34.1 \| \| *Embryo (HIV resistance)* \| 30.2 \| 69.8 \| \| *Embryos (Increased Intelligence)* \| 16.1 \| 83.9 \|   - GM wheat and somatic cells genetic modification for neuromuscular disease were said to be used by a 73 and 85% of people  - GGM for neuromuscular disease was said to be used by 65%  - As for prevention of HIV or to increase intelligence, GM was said to not be used mostly  - 10% of participants considered modification unacceptable in all scenarios and 11% accepted it  - 43 reasons were depicted as in favor of genome modification, 45 as being against and other 26 as being conditional  - These reasons could be divided in 14 domains ranging from safety, life quality, effectiveness, clinical need or alternative, ecosystems and biodiversity, justice, costs, autonomy, dignity, rights and duties, regulation, etc. |
| Uchiyama M, Nagai A, Muto K (2018)  [86] | Japan | **Awareness** of germline genome editing  **Knowledge** on CRISPR-Cas9  **Attitudes** germline genome editing | Dichotomous   - Agree/Disagree - True/False + D/K   3 point-scale   - Understand what it means, Have heard of it, Never heard of it | - respondents were questioned about the *awareness, level of understanding, criteria, and risks of germline genome editing* | \| **Gene editing** \| *Understand what means* \| *Have heard* \| *Never heard* \| \| --- \| --- \| --- \| --- \| \| *Awareness* \| 6.6 \| 26.2 \| 67.2 \|   - More than two-thirds (67%) never heard of gene editing   \| **CRISPR-Cas9** \| *Correct* \| *Incorrect* \| *Not at all* \| \| --- \| --- \| --- \| --- \| \| *Knowledge* \| 7.85 \| 11.75 \| 80.45 \|   - From the ones that understood what gene editing means, 24% answered incorrectly to knowledge question about CRISPR-Cas9   \| **Gene editing** \| *Understand what means* \| *Heave heard* \| *Never heard* \| \| --- \| --- \| --- \| --- \| \| *Diseases shorten baby’s life* \| 52.6 \| 44 \| 36-6 \| \| *Diseases require long-term* \| 53.7 \| 43.5 \| 36 \| \| *Not appropriate any circumstances* \| 34.4 \| 42.9 \| 51.7 \| \| *Concern changing human genes* \| 78.1 \| 83.5 \| 78.8 \| \| *Concern changing genes unexpected effects* \| 89.6 \| 88.4 \| 83.3 \| \| *Concern changing genes in future generations* \| 89.1 \| 88.4 \| 83.4 \|   - What is consistent is that the highest the awareness, the higher the support for gene editing in all these conditions |
| Lakomý M, Bohlin G, Hlavová R, Macháčková H, Bergman M, Lindholm M  ORION (2018)  [80] | CZ, D, IT, E, SE, UK | **Awareness** of genome editing  **Attitudes** to applications of genome editing  In somatic and germline settings | Dichotomous   - Yes/No   Qualitative  5-point scale  • From Not at all concerned to Very concerned | - *Have you ever heard of the scientific technique that enables the modification (insertion, deletion or replacement) of sections of DNA in cells and living organisms*? This technique is called genome editing.  *Q16. For what purpose do you think genome editing should be used?* Should it be used…  *a) For organ transplantation*  *b) For prevention or cure of diseases*  *c) For prevention of disabilities*  *d) For changing non-life-limiting characteristics of human embryos* (for example eye colour or strengthening the immune system)  e) For improvement of plant production  *f) For improvement of livestock production*  *- Q17. Regarding genome editing, how concerned are you about any of the following?*  a) That sufficient regulation is not in place  b) The ethical implications regarding any use of this technology  c) That the technology could be misused  d) That the technology may come with unknown side-effects in human beings | \| **Public support for human gene editing regarding…** \| *Average* \| *Not aware* \| *Aware* \| *Very/Fairly interested* \| *Neutral* \| *Not very/Not interested* \| \| --- \| --- \| --- \| --- \| --- \| --- \| --- \| \| *Organ transplantation* \| 79-90 \| 77 \| 86 \| 85 \| 73 \| 55 \| \| *Prevention/Cure diseases* \| 86-96 \| 87 \| 93 \| 93 \| 83 \| 63 \| \| *Prevention disabilities* \| 75-93 \| 81 \| 86 \| 87 \| 75 \| 57 \| \| *Changing non-life limiting characteristics of embryos* \| 29-49 \| 35 \| 41 \| 41 \| 29 \| 22 \| \| *Improvement plant production* \| 49-67 \| 53 \| 60 \| 60 \| 44 \| 36 \| \| *Improvement of livestock production* \| 34-57 \| 48 \| 49 \| 51 \| 39 \| 33 \|   - Health applications are the ones with the highest support from the public  - Awareness increases support in all applications  - Interest for the topic also increases support for applications  - 55% heard of genome editing with 45% of Czechs and 74% of Swedes  - Higher educated people have higher awareness in all countries except Czechia  - Higher levels of confidence in life sciences means higher awareness and more support of genome editing   \| **Aware** \| *Not at all concerned* \| *Not very concerned* \| *Neutral* \| *Fairly concerned* \| *Very concerned* \| \| --- \| --- \| --- \| --- \| --- \| --- \| \| *Side-effects* \| 2 \| 6 \| 17 \| 39 \| 36 \| \| *Misuse* \| 2 \| 5 \| 13 \| 34 \| 46 \| \| *Ethical implications* \| 4 \| 8 \| 23 \| 37 \| 28 \| \| *Insufficient regulation* \| 2 \| 7 \| 22 \| 39 \| 30 \| \|  \|  \|  \|  \|  \|  \| \| **Not aware** \| *Not at all concerned* \| *Not very concerned* \| *Neutral* \| *Fairly concerned* \| *Very concerned* \| \| *Side-effects* \| 2 \| 5 \| 22 \| 37 \| 34 \| \| *Misuse* \| 2 \| 4 \| 21 \| 32 \| 41 \| \| *Ethical implications* \| 3 \| 8 \| 32 \| 33 \| 24 \| \| *Insufficient regulation* \| 2 \| 6 \| 31 \| 36 \| 25 \|   - Respondents are fairly or very concerned in their majority with all types of possible issues from gene editing  - Concern seems to increase slightly with the awareness although not significantly |
| Pew Research Center (2018) | US | **Awareness** of germline genome editing  **Knowledge** on Science  **Public atittudes** to germline genome editing | Dichotomous  • Taking medical technology too far/Appropriate use of medical technology  3-point scale  • From heard a lot to Hear nothing at all  • High, medium, low  4-point scale   - From Very likely to Not at all likely | - *1) How much, if anything, have you heard or read about gene editing that can be used to change a baby’s genetic characteristics?* - *2) Do you think each of the following is an appropriate use of medical technology or is it taking medical technology too far?* - *3) Thinking about what you have heard or read, how well do you think medical researchers understand the health risks and benefits of changing a baby’s genetic characteristics?* - *4) Thinking about society as a whole …*   *How likely, if at all, do you think each of the following would be to occur if gene editing to change a baby’s genetic characteristics becomes widely available?*   - *5) If gene editing to change a baby’s genetic characteristics required testing on human embryos in order to develop these techniques, do you think this would be …*   *Knowledge 9-item list:*  *KNOSCT22. Use of a control group to determine whether a new drug is effective*  *KNOSCT23. Carbon dioxide is made as a consequence of burning fossil fuels*  *KNOSCT27. The probability of an old-bridge collapsing after a period of time*  *KNOSCT28. Only bacterial infections can be treated effectively by antibiotic medications*  *KNOSCT29. The use of a control “sugar pill” in a new drug trial is to rule out a possible placebo effect*  *KNOSCT31. The health benefits occurring when most people in a population get a vaccine is called herd immunity*  *KNOSCT32. An apple, salmon, corn and a mosquito can all be genetically modified.*  *KNOSCT33. Humans and mice share 50% or more of the same genetic makeup*  *KNOSCT34. Nitrogen makes up most of the Earth’s atmosphere.* | \| **Changing baby’s genetic characteristics to…** \| *Heard nothing at all* \| *Heard a little* \| *Heard a lot* \| \| --- \| --- \| --- \| --- \| \| *Treat a serious disease that would have at birth* \| 64 \| 75 \| 71 \| \| *Reduce the risk of serious disease occurring at lifetime* \| 52 \| 63 \| 65 \| \| *Make baby more intelligent* \| 13 \| 19 \| 31 \| \| *Test gene editing on human embryos* \| 24 \| 36 \| 49 \|  \| **If it becomes widely available to change baby’s characteristics, gene editing …** \| *Very likely* \| *Fairly likely* \| *Not too likely* \| *Not at all likely* \| \| --- \| --- \| --- \| --- \| --- \| \| *Will increase inequality (only for wealthy)* \| 58 \| 29 \| 9 \| 4 \| \| *Will be used in morally unacceptable ways* \| 54 \| 32 \| 9 \| 3 \| \| *Will be used before we fully understand its health effects* \| 46 \| 38 \| 12 \| 3 \| \| *Will pave way for new medical advances for society benefit* \| 18 \| 42 \| 30 \| 8 \| \| *Will help people live longer and with better quality* \| 16 \| 48 \| 27 \| 8 \|  \| **If it becomes widely available to change baby’s characteristics, gene editing …** \| *Heard a lot* \| *Heard little* \| *Heard nothing at all* \| \| --- \| --- \| --- \| --- \| \| *Will increase inequality (only for wealthy)* \| 64 \| 59 \| 53 \| \| *Will be used in morally unacceptable ways* \| 65 \| 54 \| 52 \| \| *Will be used before we fully understand its health effects* \| 54 \| 46 \| 43 \| \| *Will pave way for new medical advances for society benefit* \| 36 \| 16 \| 16 \| \| *Will help people live longer and with better quality* \| 34 \| 13 \| 14 \|  \| **Changing baby’s genetic characteristics to…** \| *Taking technology too far* \| *Appropriate use of technology* \| *Low knowledge* \| *Medium knowledge* \| *High knowledge* \| \| --- \| --- \| --- \| --- \| --- \| --- \| \| *Treat a serious disease that would have at birth* \| 27 \| 72 \| 58 \| 72 \| 86 \| \| *Reduce the risk of serious disease occurring at lifetime* \| 38 \| 60 \| 49 \| 60 \| 71 \| \| *Make baby more intelligent* \| 80 \| 19 \| 21 \| 17 \| 24 \| \| *Test gene editing on human embryos* \| 24 \| 43 \| 26 \| 30 \| 50 \|   - 36% of US respondents say that medical researchers understand health effects of gene editing for babies agains other 62%  - Of the ones who heard a lot of gene editing for babies, 51% agree that these professionals understand the health effects compared to 30% who heard nothing at all  - Knowledge doesn’t have an impact on this view: 36% of high science knowledge vs 40% with low knowledge |

| Funk C and Heferon M  Pew Research Center (2018b)  [65] | US | **Knowledge** on Science  **Acceptance** of genetic intervention of animals:  - Food  - Medical  - Mosquitoes | Dichotomous   - Taking medical technology too far/Appropriate use of medical technology   3-point scale  • High, medium, low | *Genetic engineering can be used to change the genetic characteristics of animals*. Would it be taking technology too far or an appropriate use of technology if using genetic engineering in …  … *mosquitoes that would prevent them from reproducing in order to prevent the spread of some mosquito-borne diseases*  … *animals to grow organs or tissues that can be used for humans needing a transplant*  … *animals to increase their production of specific proteins that will lead to more nutritious meat*  … *a closely-related species by bringing back an animal that is currently extinct*  *… aquarium fish to change their appearance, causing them to glow*  How many that think genetic engineering in these applications is appropriate…  *… oppose animal research?*  *… favor animal research?*  What is the MAIN REASON you think that would be taking technology too far?  *- Genetic engineering of animals to increase their production of specific proteins that will lead to more nutritious meat*  *- Bringing back an animal that is currently extinct by genetically engineering a closely-related species*  *- Genetic engineering of mosquitoes that would prevent them from reproducing in order to prevent the spread of some mosquito-borne diseases*  *- Genetic engineering of animals to grow organs or tissues that can be used for humans needing a transplant*  *- Genetic engineering of aquarium fish to change their appearance, causing them to glo*w  Which reasons do you point to favor or reject any of these applications? | \| **Genetic engineering in …** \| *Taking technology too far* \| *Appropriate use of technology* \| *The ones that say genetic engineering is appropriate* \| \| \| --- \| --- \| --- \| --- \| --- \| \| *Oppose animal research* \| *Favor animal research* \| \| *Mosquitoes to prevent spread of disease* \| 29 \| 70 \| 66 \| 74 \| \| *Animals to grow organs for transplant* \| 41 \| 57 \| 47 \| 69 \| \| *Animals to produce more nutritious meat* \| 55 \| 43 \| 34 \| 55 \| \| *A closely related species to rescue extinct animals* \| 67 \| 32 \| 28 \| 37 \| \| *Aquarium fish to make them glow* \| 77 \| 21 \| 15 \| 29 \|   - Health-related applications are the ones seen as appropriate use of technology by respondents  - Rescuing extinct animals and making aquarium fish to glow is taking technology too far  - Animals to produce more nutritious meat is an undecided application  - In general, respondents favor animal research when they consider any of the applications as appropriate, despite with different levels of acceptance  - 52% that oppose use of animals in scientific research consider genetic engineering of animals as taking technology too far   \| **Support for genetic engineering in …** \| *Low knowledge* \| *Medium knowledge* \| *High knowledge* \| \| --- \| --- \| --- \| --- \| \| *Mosquitoes to prevent spread of disease* \| 66 \| 72 \| 74 \| \| *Animals to grow organs for transplant* \| 47 \| 55 \| 72 \| \| *Animals to produce more nutritious meat* \| 36 \| 40 \| 53 \| \| *A closely related species to rescue extinct animals* \| 26 \| 27 \| 47 \| \| *Aquarium fish to make them glow* \| 18 \| 17 \| 33 \|   - Support for any of the applications is followed by higher level of knowledge from respondents  Qualitative  - 30% of object GE of mosquitoes mention disrupting nature or interfering with God’s plan as reasons  - Reduction of fertility in mosquitoes is believed to cause impact on ecosystems by 24%  - Growing organs for transplants is seen by 21% of people as using animals for human benefit and by 16% as potential risks for human health  - Opponents to genetic engineering to produce more nutritious meat mention unknown risks (20%), messing with nature and God’s plan (19%)  - Opponents to bringing back extinct animals say there is a reason for their original extinction (18%), there is potential risks for other species (12%) and 14% don’t see a benefit to humans  - Changing appearance of aquarium fish is seen as unnecessary and waste of resources for society or humans by 48% |
| --- | --- | --- | --- | --- | --- | --- | --- | --- | --- | --- | --- | --- | --- | --- | --- | --- | --- | --- | --- | --- | --- | --- | --- | --- | --- | --- | --- | --- | --- | --- | --- | --- | --- | --- | --- | --- | --- | --- | --- | --- | --- | --- | --- | --- | --- | --- | --- | --- | --- | --- | --- | --- | --- | --- | --- | --- | --- | --- | --- | --- | --- |
| McCaughey T, Budden DM, Sanfilippo PG, Gooden GEC, Fan L, Fenwick E, et al (2019)  [73] | Global | **Awareness** of gene editing  **Attitudes** towards germline editing   - Non-health scenarios   **Opinions** on application of gene editing   - Somatic and germline | 5-point Likert scale 🡪 3-point Likert scale  • From strongly agree to strongly disagree + D/K 🡪 Agree, neutral, disagree  Qualitative   - Open-end answers   Dichotomous   - Yes/No | Have you ever heard of human genetic engineering or gene editing?  *How much do you agree with the use of genetic editing of cells in ...*  *.... children or adults to cure a life threatening disease?*  *... children or adults to cure a debilitating disease?*  *... embryos to prevent a life threatening disease?*  *... embryos to prevent a debilitating disease?*  *... embryos to alter any non-disease characteristic?*  *How much do you agree with the use of genetic editing of cells in embryos to alter any non-disease characteristic - such as memory, eye colour or height?* This would mean that all subsequent generations would have the same genetic characteristics.  … *If you could …*  *… safely genetically edit your embryo (…) to determine physical appearance* (eye colour; hair colour; skin colour)?  *… safely genetically edit your embryo (…) to determine intelligence?*  *… safely genetically edit your embryo (…) to determine strength or sporting ability?*  *- What other non-health related traits would you edit?* [*Free text response]*  *- How much do you agree with the use of genetically modified food?*  - We are also interested in understanding the reasons for your answers. Please describe the factors that have influenced your response or attitude towards human gene engineering. [*Free text response]*  The second section asked participants their *opinions on the application of gene editing including its use in somatic cells and embryos for life threatening and debilitating diseases*  Participants were also asked *their attitudes towards embryonic editing for non health-related purposes such as to alter physical appearance, intelligence or sporting ability*. | \| **Gene editing agreement (%)** \| *Disagree* \| *Neutral* \| *Agree* \| \| --- \| --- \| --- \| --- \| \| *Children/adults to cure life-threatening diseases* \| 10 \| 31 \| 59 \| \| *Children/adults to cure a debilitating disease* \| 10 \| 30 \| 59 \| \| *Embryos to prevent life-threatening disease* \| 12 \| 26 \| 63 \| \| *Embryos to prevent a debilitating disease* \| 11 \| 25 \| 63 \| \| *Embryos to alter non-disease characteristic* \| 43 \| 30 \| 27 \|   **-** All applications with health purposes (cure/prevent diseases) are seen as reasons to agree with gene editing regardless of being children or adults  - Embryo editing is even more favoured than editing of children/adults due  - Non-health related purposes received the lowest support reaching 43% of disagreement among people.  - Of the 27% that agreed with it, pointed intelligence as the highest acceptance trait (68%) for it followed by physical strength and sporting ability (58.4%) and lastly, by appearance (51%)  - Opponents to gene editing in somatic cells showed higher use of expressions about “Better understanding” and less frequent of “Religious Beliefs” nor “Natural Selection”  - Embryonic gene editing had over-representation of expressions like “Better Understanding” and not much about “Future generations” nor “Children”  - For non-health-related traits, proponents of gene editing also didn’t mention that much “Future generations” but did it with “Children”  - Higher knowledge of gene editing beforehand meant more discussion of “Children” and less of “Future generations” |
| Critchley C, Nicol D, Bruce G, Walshe J, Treleaven T, Tuch B (2019)  [70] | AU | **Public attitudes** to Embryo, germ and somatic cell editing:   - Health - Human research - Animal research - Enhancement - Animals for food   **Knowledge** on gene editing | 10-point scale   - 0= I know nothing about gene editing, 10= I know a great deal about gene editing   4-point scale  • From Strongly agree to Strongly disagree + Unsure | - *How would you rate your current knowledge on gene editing?* - *“For each of the following…*   *… Human embryos to improve health or prevent disease (e.g., cancer)*  *… Human embryos for reasons of producing a baby with certain genes (e.g., for hair colour, gender selection)*  *… Human embryos for research purposes only*  *… Human reproductive cells (egg or sperm) for research purposes only*  *Swinburne National Technology and Society Monitory 2017 – Survey script 3*  *… Human reproductive cells (egg or sperm) to improve health or prevent disease (e.g., cancer)*  *… Human reproductive cells (egg or sperm) for reasons of producing a baby with certain genes*  *… Animal embryos for research purposes only*  *… Animal reproductive cells (egg or sperm) for research purposes only*  *… Animal embryos for human purposes (e.g., improving the quality of beef)*  *… Animal reproductive cells (egg or sperm) for human purposes (e.g., improving the quality of beef)*  *… A human body cell (e.g., eye or heart cell) to improve health or prevent disease (e.g., blindness)*  *… A human body cell to change one’s appearance*  *… A human body cell for research purposes only*  *… An animal body cell to alter its appearance (e.g., coat patterns in cats or dogs)*  *… An animal body cell to alter its characteristics for human purposes (e.g., leaner beef in cows)*  *… An animal body cell for research purposes only*  *… how much do you agree with it …?* | - Low knowledge in 2 groups interviewed. The highest the knowledge the stronger the support  - Somatic cells were more supported than germ cells for human health and human research settings  - Hereditary concerns are more important for human health improvement and research on human cells and less for animal research, enhancement and animals for food applications   \| **Gene editing application** \| *Embryo* \| *Germ* \| *Somatic* \| \| --- \| --- \| --- \| --- \| \| *Improve health/Prevent disease* \| 3.07-2.97 \| 3.15-3.06 \| 3.21-3.15 \| \| *Research on human cells* \| 2.63-2.48 \| 2.82-2.80 \| 2.85-2.90 \| \| *Research on animal cells* \| 2.80-2.75 \| 2.85-2.81 \| 2.83-2.85 \| \| *Change appearance (Human cells)* \| 1.74-1.84 \| 2.01-2.12 \| 1.97-1.9 \| \| *Animals for food* \| 2.41-2.38 \| 2.41-2.4 \| 2.28-2.29 \|   - Animal research had no difference between somatic and germline  - Support was higher for germ than somatic cells in enhancement and animals for food applications  - Moral concern is seen in all applications except editing embryos in animals for food  - Moral concern is more “pervasive” than hereditary concern in applications of gene editing for humans |
| McConnachie E, Hotzel MJ, Robbins JA, Shriver A, Weary DM, von Keyserlingk MAG (2019)  [68] | US | **Awareness** to GM cows to be hornless  **Knowledge** on gene modification  **Public attitudes** to genetic modification of cattle   - To be hornless - Consume products - Risky/beneficial | 7-point Likert scale  • 1= Nothing at all to 7= A great deal  • 1= a very bad thing, 4= nor good or bad, 7= a very good thing  • 1=Not at all risky/beneficial to 1=Very risky/beneficial  •1= Strongly disagree; 7= Strongly agree | - *“How much have you heard or read about. . .” “horn removal in cattle”, “genetic modification”, and “genetically modifying cows to be hornless”)* - *Do you think genetically modifying cows to be hornless would be. . .”,?* - Would you *be willing to consume products from these modified animals?* - *How risky and beneficial do you perceive to be GM cattle to be hornless?* - *How willing to consume derived foods from these would you be?* - *How willing do you think the average American would be?* - *Knowledge about the process of gene modification was assessed using five items modified from Hallman et al. [24]. The number of correct responses (ranging from 0 to 6) was used to create a knowledge score.* - *What considerations about GM cows would you have?* - *How are your attitudes on GM cows about…?*   *… Animal welfare*  *… Uncertainty towards technology*  *… Worker well-being*  *… Moral considerations*  *…Trade-off perspective*  *… Seeks alternative*  *… Naturalness*  *… Opposition to GM*  *… Economics*  *… Consumption* | \|  \| *GM cows to be hornless* \| *Dehorning practice* \| \| --- \| --- \| --- \| \| *Heard/Read nothing* \| 80 \| 61 \| \| *How good* \| 66 \| - \| \| *Willingness to consume products* \| 66 \| - \|   - A large majority (80%) of people haven’t read or heard nothing about GM cows to be hornless  - Two-thirds say this is a good thing and two-thirds also say they are willing to consume products derived from hornless GM cows   \| **GM cattle to be hornless** \| 1 \| 2 \| 3 \| 4 \| 5 \| 6 \| 7 \| \| --- \| --- \| --- \| --- \| --- \| --- \| --- \| --- \| \| *Would be…* \| 8 \| 10 \| 5 \| 12 \| 20 \| 22 \| 28 \| \| *How Risky* \| 15 \| 18 \| 13 \| 20 \| 14 \| 12 \| 11 \| \| *Willing to consume derived foods* \| 9 \| 8 \| 5 \| 12 \| 17 \| 28 \| 23 \| \| *Average American willingness to consume derived foods* \| 2 \| 4 \| 7 \| 20 \| 27 \| 32 \| 13 \|   1 – Very bad/Not at all risky/Strongly disagree  7 – Very good/Very risky/Strongly agree  - GM cattle to be hornless was seen as somewhat risky in general  - Foods from GM cattle to be hornless were mostly agreed by consumers and they thought similarly for the average Americans  - Self-reported willingness to consume was correlated with willingness of participants and average American willingness to consume  - GM cattle to be hornless was perceived as having lower risks than lower benefits  - Perceived benefits were positively associated with positive attitudes and willingness to consume products derived  - Perceived risks were associated with negative attitudes and lower willingness to consume products derived   \| **“GM cows would be…” (question)** \| *Responses* \| *Positive* \| *Neutral* \| *Negative* \| \| --- \| --- \| --- \| --- \| --- \| \| *Animal welfare* \| 72.4 \| 85 \| 7 \| 8 \| \| *Uncertainty towards technology* \| 17.1 \| 45 \| 25 \| 30 \| \| *Worker well-being* \| 14.8 \| 85 \| 10 \| 5 \| \| *Moral considerations* \| 14.5 \| 35 \| 15 \| 50 \| \| *Trade-off perspective* \| 12 \| 65 \| 26 \| 9 \| \| *Seeks alternative* \| 11.5 \| 35 \| 5 \| 60 \| \| *Naturalness* \| 10.8 \| 10 \| 20 \| 70 \| \| *Opposition to gene modification* \| 9.2 \| 20 \| 10 \| 70 \| \| *Economics* \| 8.3 \| 90 \| 7 \| 3 \| \| *Consumption* \| 8.1 \| 38 \| 27 \| 35 \|   - Animal welfare was the question mostly raised by respondents about GM cows and the justification that got the most positive attitude along with worker well-being  - Economics was the one that got the more positive attitude. On the other hand, opposition to GM and naturalness got the more negative attitude  - The higher the knowledge the more positive attitudes and higher willingness to consume products from GM cows |
| Yunes MC, Teixeira DL, von Keyserlingk MAG, Hotzel MJ (2019)  [69] | Brazil | **Awareness** of common pig practices  **Knowledge** on genetics and biotechnology  **Acceptance** of gene editing for boar taint in pings:  - Reduce, prevent and eliminate | 5-point Likert scale 🡪 3-point (acceptable, indifferent, not acceptable)  • From only benefits to only damage  • From strongly agree to strongly disagree  • From totally unacceptable to totally acceptable  • From no risk/benefits to high risk/benefits  • From not acceptable to very acceptable   - From totally agree to totally disagree - From no risk/benefit to high risk/benefit   Dichotomous   - True, False + D/K | - Do you think Science and Technology will bring …  *… only benefits*  *… more benefits*  *… equal benefits and damage*  *… more damage*  *… only damage*  - How much do you agree that new technologies will bring benefits despite unknown consequences?  - It is acceptable    How acceptable do you think … (are)?  *... GM vegetables for higher nutrients*  *… GM microorganisms for food*  *… GM pigs to produce more meat*  *… Meat in vitro from pig stem cells*  Participants were asked to *complete a knowledge quiz on biotechnology* that had five previously validated questions  How acceptable are the following reasons to reduce boar taint in pigs?  *- Positive effects on animal welfare*  *- Potential risks of gene editing*  *(to humans, to animals, not specific)*  *- Perceived effects on product quality*  *- Insufficient information*  *- Dislike or opposition to GM*  *- Gene editing is unnatural* | \|  \| *Only benefits* \| *More benefits* \| *Equal benefits & damage* \| *More damage* \| *Only damage* \| \| --- \| --- \| --- \| --- \| --- \| --- \| \| *Science & Technology* \| 16 \| 41 \| 41 \| 2 \| 0 \|   - Science and Technology are seen as bringing equal benefits and damage   \|  \| *Totally agree* \| *Partly agree* \| *Partly disagree* \| *Totally disagree* \| \| --- \| --- \| --- \| --- \| --- \| \| *New tech benefits despite unknown consequences* \| 7 \| 48 \| 24 \| 19 \|   - New technologies are partly agreed by the majority of respondents as bringing benefits despite the unknown consequences   \| **Biotechnologies** \| *Acceptable* \| *Intermediate* \| *Not acceptable* \| \| --- \| --- \| --- \| --- \| \| *GM vegetables for higher nutrients* \| 43 \| 20 \| 37 \| \| *GM microorganisms for food* \| 31 \| 20 \| 44 \| \| *GM pigs to produce more meat* \| 28 \| 18 \| 54 \| \| *~~Meat from Pigs fed with GM diet~~* \| ~~28~~ \| ~~26~~ \| ~~52~~ \| \| *Meat in vitro from pig stem cells* \| 25 \| 20 \| 55 \|   - GM pigs to produce more meat is seen as not acceptable by more than half (54%) of respondents   \| **Knowledge quiz** \| *% correct* \| *% D/K* \| \| --- \| --- \| --- \| \| *By eating a GM food, a person’s genes could also become modified* \| 57 \| 33 \| \| *Pigs modified with genes from a fish would probably taste fishy* \| 43 \| 52 \| \| *Ordinary tomatoes do not contain genes, while genetically modified tomatoes do* \| 39 \| 36 \| \| *It is possible to transfer animal genes into plants* \| 14 \| 56 \|   - Knowledge was not related with gene editing acceptability to prevent boar taint in pigs   \| **Reasons of gene editing to reduce boar taint in pigs** \| *Total* \| *Acceptable* \| *Intermediate* \| *Not acceptable* \| \| --- \| --- \| --- \| --- \| --- \| \| *Positive effects on animal welfare* \| 45 \| 63 \| 19 \| 16 \| \| *Potential risks of gene editing…* \| 34 \| 26 \| 61 \| 25 \| \| *(To humans)* \| (13) \| (11) \| (23) \| (6) \| \| *(To animals)* \| (9) \| (6) \| (17) \| (11) \| \| *(Not specific)* \| (10) \| (10) \| (21) \| (8) \| \| *Perceived effects on product quality* \| 11 \| 10 \| 16 \| 11 \| \| *Insufficient info* \| 10 \| 5 \| 27 \| 8 \| \| *Dislike or opposition to GM* \| 8 \| 1 \| 2 \| 38 \| \| *Gene editing is unnatural* \| 5 \| 3 \| 2 \| 14 \|   - The major reason presented to reduce boar taint in pigs was positive effects on animal welfare and it was seen as acceptable by the majority  - Potential risks were seen as moderately acceptable by the majority  - The least recognized reason recognized to reduce boar taint in pigs was unnaturalness of gene editing  - The perception of benefits is positively correlated with its acceptance  - 39% perceived much/high risks, 26% intermediate risks and 35% low or no risk  - The perception of risks is negatively correlated with its acceptance  - Animal welfare was suggested as something to face positive effects with products coming from gene-edited animals  - Animal suffering and meat contamination was mentioned by some in regards to gene editing acceptance  - Other respondents see potential of gene editing in animals as not justifiable |
| Kohl PA, Brossard D, Scheufele DA, Xenos MA (2019)  [84] | US | **Attitudes** towards gene editing in wildlife:   - Moral acceptance - Risk/Benefit - Purpose:   - decrease or eliminate a population  - Improve survival of endangered species | 5-point scale 🡪 2-point scale  • 1=Not at all risky/beneficial, 5=Very risky/beneficial  7-point scale🡪3-point  • 1=Strongly agree, 7= Strongly disagree | *Will respondents …*  *… perceive the benefits as outweighing the risks (question 1)*,  *…perceive applications to decrease or eliminate environmentally problematic wildlife populations as less morally acceptable than applications to improve survival in endangered wildlife (question 2)*  …. *have more concern about applications to decrease or eliminate environmentally problematic wildlife populations being used for the wrong purposes than applications to improve survival in endangered wildlife (question 3)?»* | - More than 80% of people says gene editing in wildlife is at least somewhat risky for both humans and nature  - A majority despite lower says gene editing in wildlife is somewhat beneficial for both humans and nature   \| **Gene editing in wildlife** \| *Mostly or not at all* \| *At least somewhat* \| \| --- \| --- \| --- \| \| *Risky for humans* \| 15.6 \| 84.4 \| \| *Risky for nature* \| 12.8 \| 87.2 \| \| *Beneficial for humans* \| 37 \| 63 \| \| *Beneficial for nature* \| 45.5 \| 54.5 \|   - More than 70% think wildlife gene editing could be used for the wrong purposes  - Gene editing in wildlife means more concern about risks and moral acceptability and skepticism about benefits, being advised a cautious approach  - Around 71% of respondents says gene editing in wildlife messes with nature and close to 60% mentions humans playing God  - Gene editing in wildlife is seen with a higher risk perception than GM food   \| **Gene editing in wildlife** \| *Disagree* \| *Neither agree or disagree* \| *Agree* \| \| --- \| --- \| --- \| --- \| \| *Morally acceptable to decrease or eliminate* \| 38.5 \| 29.7 \| 31.8 \| \| *Morally acceptable to improve survival* \| 29.4 \| 33.3 \| 37.3 \|   - More people agree that gene editing in wildlife is morally acceptable to improve survival than to decrease or eliminate populations |
| Lull RB, Akin H, Hallman WK, Brossard D, Jamieson KH (2019)  [66] | US | **Knowledge** about Zika  **Approval** of genetically modified mosquitoes:  - Risk/Benefit perceptions  - GE food approval  - Zika concern | 5-point scale  • 1= Strongly disagree, 5= Strongly agree  • 1= Strongly disapprove, 5= Strongly approve | Releasing genetically modified mosquitoes to reduce the mosquito population…  … *threatens the natural order of things*  …. *is likely to have harmful consequences*  …. *could significantly reduce the spread of Zika virus*  How much would you approve releasing GM mosquitoes to reduce mosquito population  … in the area where you live that *were capable of carrying Zika virus*?  … in the area where you live that were *definitely carrying Zika virus*?  … if there were *people infected* with Zika virus in the area where you live? | - Negative association found between GE mosquito risk perception and approval  - Positive association found between GE mosquito benefit perception and approval  - Benefit perception is higher on GE mosquito approval than risk perception  - GE food approval has some influence on GE mosquito risk and benefit perceptions  - No association between Zika concern and GE mosquito approval  - Zika issue involvement could have some effects on GE mosquito approval despite very upstream in the model tested |
